# Quantitative Determination of Flexible Pharmacological Mechanisms Based On Topological Variation in Mice Anti-Ischemic Modular Networks

**DOI:** 10.1371/journal.pone.0158379

**Published:** 2016-07-06

**Authors:** Yin-ying Chen, Ya-nan Yu, Ying-ying Zhang, Bing Li, Jun Liu, Dong-feng Li, Ping Wu, Jie Wang, Zhong Wang, Yong-yan Wang

**Affiliations:** 1 Institute of Basic Research in Clinical Medicine, China Academy of Chinese Medical Sciences, Beijing, China; 2 Guang'anmen Hospital, China Academy of Chinese Medical Sciences, Beijing, China; 3 School of Mathematical Sciences, Peking University, Beijing, China; Semmelweis University, HUNGARY

## Abstract

Targeting modules or signalings may open a new path to understanding the complex pharmacological mechanisms of reversing disease processes. However, determining how to quantify the structural alteration of these signalings or modules in pharmacological networks poses a great challenge towards realizing rational drug use in clinical medicine. Here, we explore a novel approach for dynamic comparative and quantitative analysis of the topological structural variation of modules in molecular networks, proposing the concept of allosteric modules (*AMs*). Based on the ischemic brain of mice, we optimize module distribution in different compound-dependent modular networks by using the minimum entropy criterion and then calculate the variation in similarity values of *AM*s under various conditions using a novel method of SimiNEF. The diverse pharmacological dynamic stereo-scrolls of *AM*s with functional gradient alteration, which consist of five types of *AM*s, may robustly deconstruct modular networks under the same ischemic conditions. The concept of *AM*s can not only integrate the responsive mechanisms of different compounds based on topological cascading variation but also obtain valuable structural information about disease and pharmacological networks beyond pathway analysis. We thereby provide a new systemic quantitative strategy for rationally determining pharmacological mechanisms of altered modular networks based on topological variation.

## Introduction

The spatial structure of cell signaling systems [1] represents a promising pathway for developing a better approach to studying the complexity of diseases and unraveling the mechanisms of pharmacological networks. Moreover, modularity is ubiquitous in biological networks [2], and the exploration of modular structure has been proposed as a key factor for understanding the complexity of biological systems [3] and disease networks [4]. A cascade of network modules is used to define cancer progression, and modular structure plays a significant role in aiding the diagnosis, prevention, and therapeutic treatment of the disease [5]. Because multiple drugs act within the context of the regulatory networks in which drug targets and disease gene products function, module-designed studies are becoming increasingly important for revealing the relationships between drug actions and disease outcomes in network pharmacology [6]. Highly networked signaling hubs are often associated with disease, for example, the IKK-IkB-NFkB signaling module functions as a signaling hub for diverse inflammatory, immune, and developmental signals [7]. Modular pharmacology (MP) also suggests that the treatment of complex diseases requires a modular design to affect multiple targets [8]. To date, many methods or algorithms have been proposed for module identification [3].

From a network point of view, however, most intra-protein conformational changes may be dynamically transmitted across protein-protein interactions and signaling networks of the cell [9]. Allostery exerts conformational control over cellular pathways and networks to determine cell responses; and if allostery is not at play, neither signal propagation nor pathway switching will take place [10]. A previous study analyzed several methods for identifying allosteric pathways in intra-protein networks, including one method that employed the concept of modules and considered proteins as sets of modules [9]. Allosteric networks have been characterized using a community network analysis approach previously applied to investigate allostery in tRNA-protein complexes [11], protein dynamical network [12] and innovative therapies [13].

Because allosteric conformational change involves the relative movement of both internal and external modules [14], allosteric communication plays a crucial role in pharmacological cellular signaling processes. Positive and negative allosteric modulators of the type 5 metabotropic glutamate (mGlu5) receptor both have demonstrable therapeutic potential in neurological and psychiatric disorders [15]. Therefore, by fusing the regulatory principles of protein allostery (a special and limited part in cellular networks) and dynamic network information, we propose the concept of allosteric modules (*AM*s) [16]. Generally, the multi-potent functional changes in modular architecture are referred to as *AM*s. Allostery is an intrinsic property of many modules that is indispensable for molecular regulatory and feedback mechanisms. An *AM* may change its boundary in structural transformation based on different parametric variations (such as nodes and edges) [16], which can be used to reflect the dynamics of modular networks and quantitatively analyze allosteric variations to reveal detailed allosteric pharmacological events in cellular networks.

Our previous studies showed that baicalin (BA), cholic acid (CA), and jasminoidin (JA) could significantly reduce ischemic infarct volume [17,18]. Moreover, analysis of differentially expressed genes and signaling pathways indicated that the pharmacological mechanisms of these compounds showed both similarities and variations [17,19]. Based on these findings, we attempted to quantitatively determine the diversity of *AM*s in compound-related target networks fusing topological variation and functional alteration and to further reveal the comparative pharmacological mechanisms of different compound treatments toward cerebral ischemia according to the variability of allostery-of-function modules.

## Materials and Methods

### Animal model, compound treatment, microarray experiments and data preparation

The animal model, compound treatment, microarray experiment and data preparation method used in this study have been previously described [18,20]. Animal use protocols were reviewed and approved by the Ethics Review Committee for Animal Experimentation, China Academy of Chinese Medical Sciences. All animal experiments were conducted in accordance with the Prevention of Cruelty to Animals Act 1986 and the National Institute of Health guidelines on the care and use of laboratory animals for experimental procedures. All surgery was performed under anesthesia, and all efforts were made to minimize suffering.

Briefly, one hundred and ten adult male mice (3 months old, 38–48 g, Kunming strain, China) were purchased from the Experimental Animal Center of Peking University Health Science Center, and then randomly divided into five groups based on previous studies: sham, vehicle, BA, CA and JA, each consisting of 22 subjects. A cerebral ischemia–reperfusion mouse model was established based on methods described by Hara et al. [21] and Himori et al. [22]. Briefly, after being anesthetized with 2% pentobarbital (4 mg/kg, i.p.), the mice were subjected to middle cerebral artery occlusion, ligated with an intraluminal filament for 1.5 h and then reperfused for 24 h. In the sham- operated mice, the external carotid artery was surgically prepared for the insertion of the filament, but the filament was not inserted. Based on the infarction volume or behaviors of these mice [23], we could determine whether the operations were successful. Mice in the experimental groups were injected with 2 ml/kg body weight BA (5 mg/ml), CA (7 mg/ml) and JA (25 mg/ml) via the tail vein 2 h after surgical occlusion. Mice in the sham-operated and vehicle groups underwent identical procedures, but were injected with vehicle (2 ml/kg body weight; 0.9% NaCl) rather than experimental compounds. During the experimental procedure, blood pressure, blood gas, and glucose levels were monitored, rectal temperature was maintained at 37.0–37.5°C with a heating pad, the body temperature was maintained at 37°C with a thermostatically controlled infrared lamp, and brain temperature (monitored with a 29-gauge thermocouple in the right corpus striatum) was maintained at 36–37°C with a temperature-regulating lamp. Electroencephalogram monitoring was performed to ensure isoelectricity during ischemia.

After 24 h reperfusion, 13 mice from each group were anesthetized with chloral hydrate (400 mg/kg) and decapitated rapidly. The cerebrum was removed and cut into five slices. The slices were transferred to 4% 2, 3, 5-triphenyltetrazolium chloride solution and incubated for 30 min at 37°C in darkness and then transferred into a 10% formalin solution. The area of the infarct region was calculated using a Pathology Image Analysis System, and the ratio of the infarct volume to the total slice was also calculated. 9 mice from each group were sacrificed by rapid decapitation under deep anesthesia with chloral hydrate (400 mg/kg). Hippocampal RNA from different treatment groups was homogenized in TRIzol Reagent and extracted according to the single-step method [24]. RNA was further purified to remove genomic DNA contamination and concentrated using an RNeasy micro kit (Qiagen, Valencia, CA). RNA quality was assessed by determining the 26S/18S ratio using a Bioanalyzer microchip (Agilent, Palo Alto, CA). Microarrays were made from a collection of 16,463 mouse oligo chips provided by the Boao Biotech Company, Beijing.

All experimental data were uploaded to the ArrayTrack system [US Food and Drug Administration (FDA), USA]. Experimental analysis was based on the Minimum Information About a Microarray Experiment (MIAME) guidelines and the MicroArray Quality Control (MAQC) project. The results were submitted to the Array Express database. All microarray data were normalized by locally weighted linear regression (Lowess) to reduce the experimental variability [25] (smoothing factor: 0.2; robustness iterations: 3). A one-way ANOVA model and a significance analysis of microarrays (SAM) were used to compare the means of the altered genes between vehicle and sham, BA and vehicle, CA and vehicle, JA and vehicle groups. Genes with a P-value < 0.05 and a fold change >1.5 were selected for further analysis. After obtaining the P-values, Bonferroni correction was performed to select a list of significant genes for further analysis. In addition, an increase > 1.5-fold or a decrease < 0.5-fold of expression levels indicated up-regulation or down-regulation, respectively.

### Constructing target networks in different groups

We constructed global networks of different groups by integrating gene expression data and PPI information. A unique global mice gene and protein network was constructed by integrating protein interactions reported in the BIOGRID [26], INTACT [27], MINT [28], and NIA Mouse Protein-Protein Interaction Databases [29] and by deleting duplicated data and self-interactions.

Gene expression data were adapted from our previous study of gene expression profiles of the hippocampus of ischemic mice treated with baicalin (BA), cholic acid (CA) and jasminoidin (JA) [18,20]. Mean-centered normalization of expression data was performed. Genes with an expression value greater than one were defined to be significantly differentially expressed. These genes were then mapped to the interaction network, generating target networks for each group. The topological characteristics of related target networks were analyzed.

### Identifying functional modules in different groups

In related target networks of each group, functional modules were identified using Affinity propagation (AP) [30], the Markov Cluster algorithm (MCL) [31] and Molecular Complex Detection (MCODE) [32], respectively. For the AP algorithm, we sampled the Preference parameters from 0.1 to 1.0 in steps of 0.1. For MCL, the range of possible Inflation parameter values (1.5 to 5.0) was sampled uniformly with a step size of 0.5. For MCODE, we tried all possible combinations of the following parameters (Include Loops: false; Degree Cutoff: 3; Node Score Cutoff: 0.2; Haircut: true or false; Fluff: true or false; K-Core: 2; Max. Depth from Seed: 100, 5, 4, 3).

### Calculating minimal network entropy

After identifying functional modules by three different methods, the next task was to determine the relative optimal module identification results. In this study, we attempted to assess the module identification results by incorporating the notion of entropy. The entropy of a random variable quantifies the uncertainty or randomness of that variable [33]. Some researchers have provided definitions of the network structure entropy, which is based on node degree and indicates the homogeneity of node degree [34,35]. The importance of nodes is defined as follows:

$$I_{i}=k_{i}/\sum_{i=1}^{N}k_{i}$$ (1)

where *I*_*i*_ is the importance of node *i*, *N* is the number of nodes in the network, and *k*_*i*_ is the degree of node *i*. The network structure entropy is defined as follows:

$$E=-\sum_{i=1}^{N}I_{i}\text{ln}\;I_{i}$$ (2)

In scale-free networks, a large number of low-degree peripheral nodes are linked to a few high-degree hubs; these networks are considered to be “ordered” [34,35]. The minimum entropy value is *E*_min_,

$$E_{\text{min}}=-\frac{1}{2}\text{ln}\frac{1}{2}-\sum_{i=2}^{N}\frac{1}{2(N-1)}\text{ln}\frac{1}{2(N-1)}=\frac{\text{ln}\;4(N-1)}{2}$$ (3)

It is believed that the ultimate aim of module identification is to find a stable modular state, which should have minimum uncertainty. Because the number of modules in a given network is uncertain in advance, the only task that can be completed is to minimize the uncertainty. Because minimum entropy indicates minimum uncertainty, we proposed to evaluate the results of module identification based on the minimum entropy criterion. To assess the statistical significance of the minimum network entropy of each network, an ensemble of randomized networks was constructed by randomly reshuffling all the edges of the original network [36,37]. With this type of randomization, each node preserved the same number of links as in the original network.

### Enrichment analysis of gene ontology (GO) categories and KEGG pathways

Significantly over-represented GO biological processes (BP) in modules were detected by the DAVID 6.7 functional annotation tool (http://david.abcc.ncifcrf.gov/) [38] (GOTERM_BP_ALL). The analysis was conducted using a modified Fisher's exact test, and we selected all GO terms that were significant with a P-value <0.05 after correcting for multiple-term testing by Benjamini. Enrichment analysis of KEGG pathways in modules was performed using a hypergeometric test, as implemented on the KOBAS 2.0 web server (http://kobas.cbi.pku.edu.cn/) [39].

### Use of SimiNEF to calculate similarities of *AM*s

A method that integrated the similarities of nodes, edges and GO functions of modules (SimiNEF) was proposed and applied to compare the degree of overlap between *AM*s, focusing particularly on node allosteric modules (^N^*AMs*) and edge allosteric modules (^E^*AMs*) in any two groups. In SimiNEF, we used similarity *S*_*nef*_ to quantify the relative overlap between *AM*s *m*_*i*_ and *m*_*j*_, including the similarities of nodes (*S*_*n*_), edges (*S*_*e*_) and GO functions (*S*_*f*_) altogether. SimiNEF was based on Jaccard’s coefficient of similarity, which ranges from 0% (states have no nodes/edges/ GO functions in common) to 100% (states have identical nodes/edges/GO functions). The *S*_*n*_ (*m*_*i*_, *m*_*j*_), *S*_*e*_ (*m*_*i*_, *m*_*j*_) and *S*_*f*_ (*m*_*i*_, *m*_*j*_) are defined by Eqs 4, 5 and 6, respectively.

$$S_{n}\;(m_{i},m_{j})=\frac{\left|N(m_{i})\cap N(m_{j})\right|}{\left|N(m_{i})\cup N(m_{j})\right|}$$ (4)

$$S_{e}\;(m_{i},m_{j})=\frac{\left|E(m_{i})\cap E(m_{j})\right|}{\left|E(m_{i})\cup E(m_{j})\right|}$$ (5)

$$S_{f}\;(m_{i},m_{j})=\frac{\left|F(m_{i})\cap F(m_{j})\right|}{\left|F(m_{i})\cup F(m_{j})\right|}$$ (6)

where |*N*(*m*_*i*_) ∩ *N*(*m*_*j*_)|, |*E*(*m*_*i*_) ∩ *E*(*m*_*j*_)|, and |*F*(*m*_*i*_) ∩ *F*(*m*_*j*_)| are the numbers of overlapping nodes, edges, and GO functions in *m*_*i*_ and *m*_*j*_, and |*N*(*m*_*i*_) ∪ *N*(*m*_*j*_)|, |*E*(*m*_*i*_) ∪ *E*(*m*_*j*_)|, and |*F*(*m*_*i*_) ∪ *F*(*m*_*j*_)| are the numbers of nodes, edges, and GO functions in the union of *m*_*i*_ and *m*_*j*_ (Note: Here, GO functions refer to the GO biological processes mentioned above). If *S*_*n*_, *S*_*e*_, and *S*_*f*_ are all greater than a certain value *k* simultaneously, then we establish that *S*_*nef*_ is greater than *k*.

### Biological validation for *AM*s

Two *AM*s Mrm1-Guk1-Hrsp12 and Fos-Cebpg-Atf2 were selected to validate the relationship between their mRNA or protein expressions and cerebral ischemia, as well as the effects of different compound interventions on the expression of genes or proteins using RT-PCR and western blotting, respectively. The experiment, including animal model and compound treatment, was performed as described previously (see section 1 in Materials and Methods).

#### Real-time reverse transcription-polymerase chain reaction (RT-PCR)

Eight animals from each group were anesthetized with chloral hydrate (400 mg/kg). Euthanasia was performed by rapid decapitation under deep anesthesia. Total RNA was extracted using TRIzol Reagent (Invitrogen, Carlsbad, CA). cDNA was synthesized using a First Strand cDNA Synthesis Kit (Fermentas MBI) according to the manufacturer's instructions. Expressions of *Guk1*, *Hrsp12* and *Mrm1* were determined by real-time PCR, and the following primer sequences were used: *Guk1*, 5’-TATGGGACAAGCAAGGAAGC-3’ (forward) and 5’-GGCTTCATCCAGGTTGTCAT-3’ (reverse); *Hrsp12*, 5’-CCAAGCTGTGCTAGTGGACA-3’ (forward) and 5’-GCAGCCTTCAGAATCTCACC-3’ (reverse); *Mrm1*, 5’-CAATCTTGGGGCTGTGATG-3’ (forward) and 5’-TGGCCTTGCTGACTACTGG-3’ (reverse); GAPDH, 5’-CAAAGTTGTCATGGATGACC-3’ (forward) and 5’-CCATGGAGAAGGCTGGG-3’ (reverse). Real-time PCR was performed in a 7900HT Fast Real-Time PCR System (Applied Biosystems) using 2× SYBR Green PCR Master Mix (Applied Biosystems). The data were quantified using the standard curve method after normalizing with GAPDH gene expression.

#### Western blotting

Three animals were sacrificed 24 h after ischemia by rapid decapitation under deep anesthesia. Brain tissues were prepared for western blotting. Protein concentration was determined by the Bradford assay (Tiangen Biotech Co., Ltd., Beijing, China). Protein samples (50 μg per lane) were electrophoresed in 10% SDS-polyacrylamide gels and transferred to polyvinylidene fluoride (PVDF) membranes (Millipore, Billerica, MA, USA) at 60 V for 2 hours at 4°C in a transfer buffer containing 48 mmol L^-1^ Tris-base, 39 mmol L^-1^ glycine, and 20% methanol. The blots were blocked in fresh blocking buffer (Tris-buffered saline with 0.05% Tween 20 [TBS-T] plus 5% non-fat dry milk) for 1 h at room temperature. The blots were then incubated at 4°C overnight with anti-Atf2 antibody (sc-164978), anti-c-Fos antibody (sc-52), or anti-C/EBPγ antibody (sc-25769) (all at 1:1000 dilution; Santa Cruz Biotechnology, Inc., Santa Cruz, CA, USA) and anti-β-actin antibody (1: 10000, Santa Cruz Biotechnology). A secondary antibody conjugated with horseradish peroxidase (HRP, 1:5000, Bio-Rad) was used. Immunoblots were visualized on X-ray film by a chemiluminescence reaction (Pierce, Rockford, IL). Image analysis of the blots was performed on optical density-calibrated images using AlphaEase Stand Alone software (Alpha Innotech Corp., San Leandro, CA).

## Results

**Fig 1:**
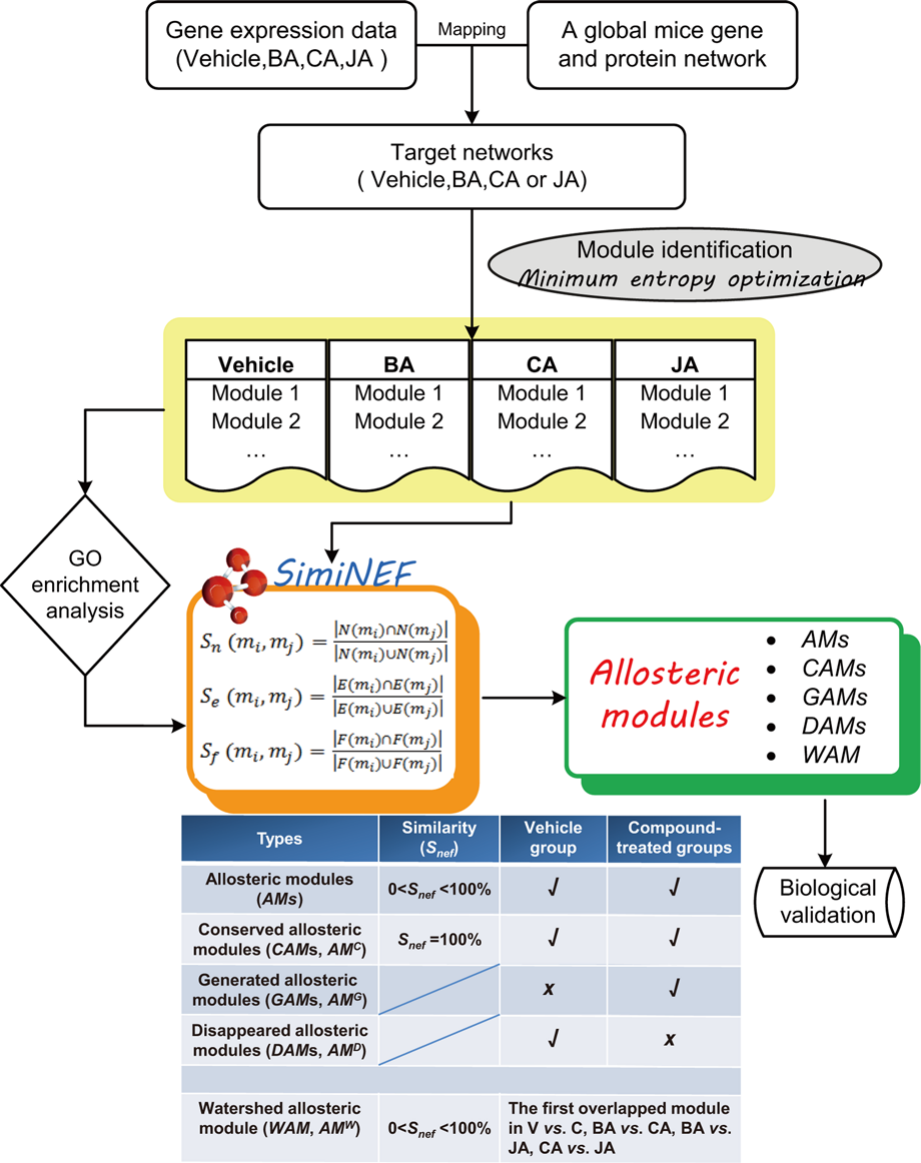
Flow diagram. Drug-target networks were constructed by integrating gene expression data and protein interaction data, and then functional modules were identified using the AP, MCL and MCODE algorithms, respectively. The results of module identification were then optimized based on the minimum entropy criterion. Enrichment analysis of GO biological processes and KEGG pathways was performed with the DAVID 6.7 software program. The similarity or overlap between modules was calculated using SimiNEF. Then, five different types of modular allostery were identified. We defined the five types of *AM*s as follows. (1) *AM*s. Most modules showed partial overlap (0<*S*_*nef*_ <100%) between vehicle-treated and compound-treated groups, as well as between various compound-treated groups, and thus they were referred to as *AM*s. (2) Conserved allosteric modules (*CAM*s, *AM*^*C*^). If the similarity between vehicle-treated group and a compound-treated group reached 100% (*S*_*nef*_ = 100%), these modules were referred to as *CAM*s. (3) Generated allosteric modules (*GAM*s, *AM*^*G*^). If a module was not found in the vehicle-treated group but could be identified in compound-treated groups, we defined it as a *GAM*. (4) Disappeared allosteric modules (*DAM*s, *AM*^*D*^). If a module was found in the vehicle-treated group but could not be identified in compound-treated groups, we defined it as a *DAM*. (5) Watershed allosteric module (*WAM*, *AM*^*W*^). 0<*S*_*nef*_ <100%, the first overlapped module between vehicle-treated and compound-treated groups, as well as between various compound-treated groups, was referred to as the *WAM*. V = Vehicle, C = Compound. ‘√’ or ‘×’ represents its appearance ‘yes’ or ‘no’ in the group, respectively. Finally, two modules could be validated using RT-PCR and Western blotting, respectively.

The protocols followed in conducting the experiment and data analysis are shown in Fig 1. In previous studies, we demonstrated that BA, JA and CA all exerted a significant pharmacological effect in reducing infarction volume and neurological scores [17,18,20].

### Comparing topological attributes of compound-dependent ischemic networks

**Fig 2:**
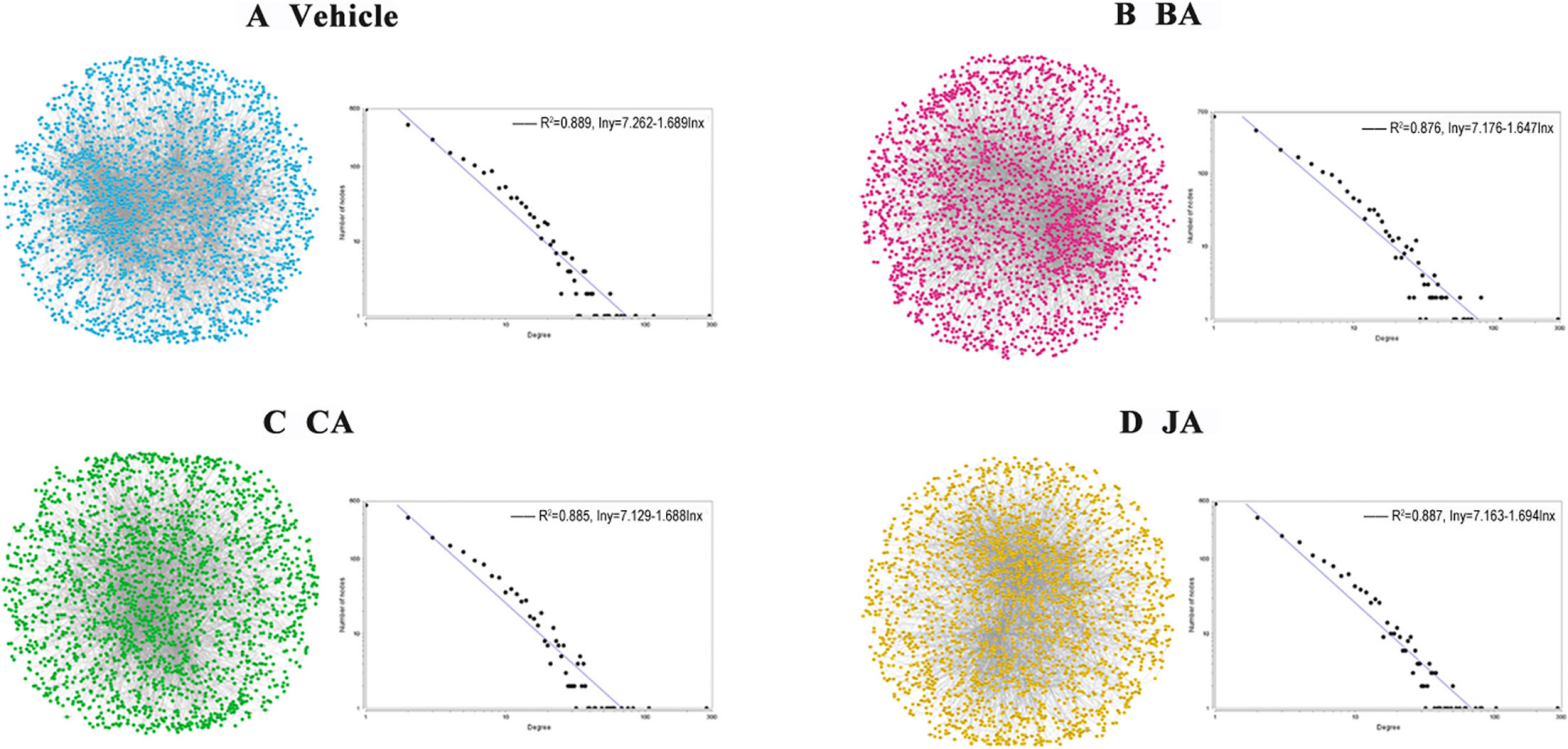
Global networks of vehicle, BA, CA, and JA groups. The degree distribution of the four networks can be approximated by a power law distribution, which appears as a straight line on a logarithmic plot.

We constructed a mice protein interaction network containing 65,850 edges by integrating multiple protein interaction databases. After the preprocessing of gene expression data, different microarray experimental data from ischemic mice were mapped to this network, and differential target networks of vehicle (vehicle vs. sham), BA (BA vs. vehicle), CA (CA vs. vehicle), and JA (JA vs. vehicle) groups were constructed (Fig 2). The topological attributes of these four networks were similar to each other, although there was a small difference in network size (S1 Table). Therefore, analysis of the entire networks might still not be sufficient to uncover the diverse pharmacological protective mechanisms among these compounds.

### Identifying functional modules from the entire ischemic networks

**Fig 3:**
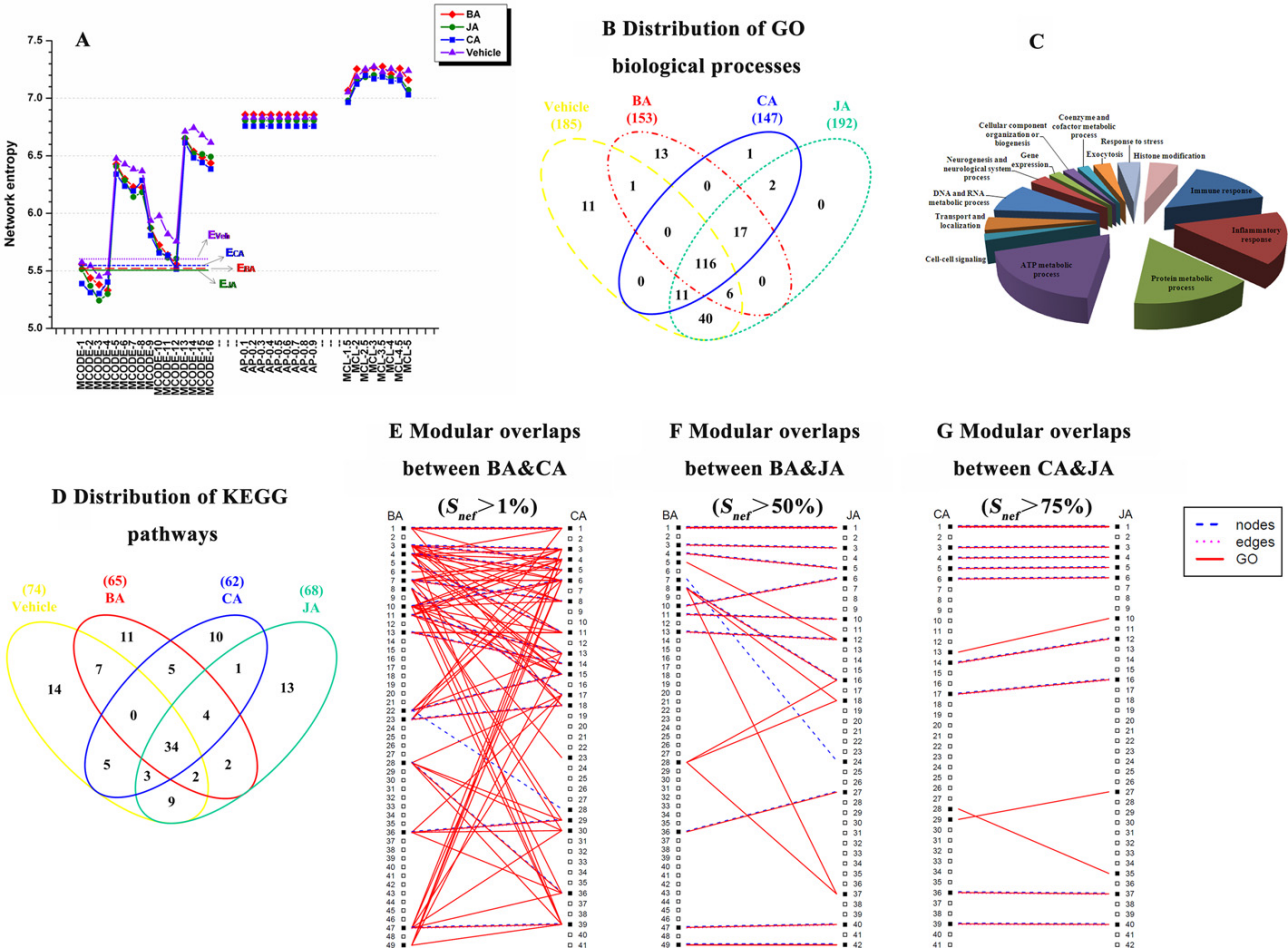
Changes in network structure entropy in different treatment groups. (**A**) The changes in network structure entropy under different parameter settings in MCODE, AP, and MCL. The red, green, blue, and violet lines denote the entropies in the BA, JA, CA and vehicle groups, respectively. E_Veh_ (violet dotted line), E_CA_ (blue short dashed line), E_BA_ (red long dashed line) and E_JA_ (green solid line) denote the mean minimal entropy in randomized networks corresponding to the vehicle, CA, BA and JA groups. (**B** and **D**) Distributions of overlapping and non-overlapping GO biological processes and KEGG pathways among groups. Only significant functions or pathways are shown in this figure based on a corrected P-value <0.05. (**C**) The 14 categories of 218 significantly enriched GO biological processes. (**E**-**G**) Examples of modular overlaps between BA and CA, BA and JA, CA and JA groups, respectively. Each column of numbers indicates the number of each module in each group. For example, if the overlap of nodes, edges or GO functions (any one of them) between two modules is greater than 1%, 50% or 75%, these two modules are connected (See also S1 Fig).

The results identified by AP, MCL, and MCODE are shown in S2–S4 Tables. Considering the effect of different parameters on the clustering results, we tested multiple parameter settings for each method. The results indicated that there were large differences in the number of modules, size, modularity and entropy among modules obtained from the three methods in each group (S2–S4 Tables, Fig 3A).

### Optimizing functional modules using MCODE approach

According to the minimum entropy criterion, results identified by MCODE, which had minimal network entropy, were selected for further analysis (Fig 3A). To evaluate the statistical significance of the minimum network entropy of each target network, we constructed an ensemble of 50 randomized networks with the same degree as the original networks in each group, and the same method was used to identify modules in randomized networks. The entropy of randomized networks was then calculated. The means of the minimum entropy in randomized networks corresponding to the vehicle, BA, CA, and JA groups were 5.60826, 5.51223, 5.52381, 5.50189, respectively (Fig 3A). The values of the minimum network entropy in the original networks were significantly lower than those in the randomized networks (*P*<0.05). After the optimization of the minimum entropy, 50, 49, 41, and 42 modules (nodes ≥3) were identified from relevant target networks using MCODE (S5 Table). The average sizes of those modules ranged from 5.327 to 6.171, and the entropy values of the four networks were similar after module optimization (S5 Table).

### Distribution of GO biological processes and KEGG pathways

GO functional enrichment analysis revealed 218 significantly enriched biological processes (S6 Table), which could be largely divided into 14 categories, including immune response and inflammatory response (Fig 3C). A total of 185, 153, 147, and 192 GO biological processes were enriched in the vehicle, BA, CA and JA groups, respectively (Fig 3B, S7 Table). Pathway analysis revealed 74, 65, 62, and 68 significantly enriched KEGG pathways in modules from the vehicle, BA, CA, and JA groups, respectively (Fig 3D, S8 Table). Based on modular analysis, we unexpectedly expanded traditional pathway analysis not only by discovering many unknown pathways in different groups but also by developing an enrichment strategy based on the variation of modular dynamics beyond known pathways.

### Associations between GO functions and topological structures of *AM*s

Within the same group, there were no significant linear correlations between the changes in the number of GO biological processes and nodes or edges in modules. We calculated the connecting percentage of each node (i.e., the degree of each node/the total degree of the module it belongs to) to indicate the importance of each node in the module. A significant linear correlation was observed between the connecting percentage of each node and the changes in the number of GO biological processes in the same module, and the correlation coefficient value was only 0.18. These results suggested that local topological variations, such as changes in nodes or edges, might not lead to sufficient functional alteration of the network.

### Similarity gradient among *AM*s using SimiNEF in different groups

**Fig 4:**
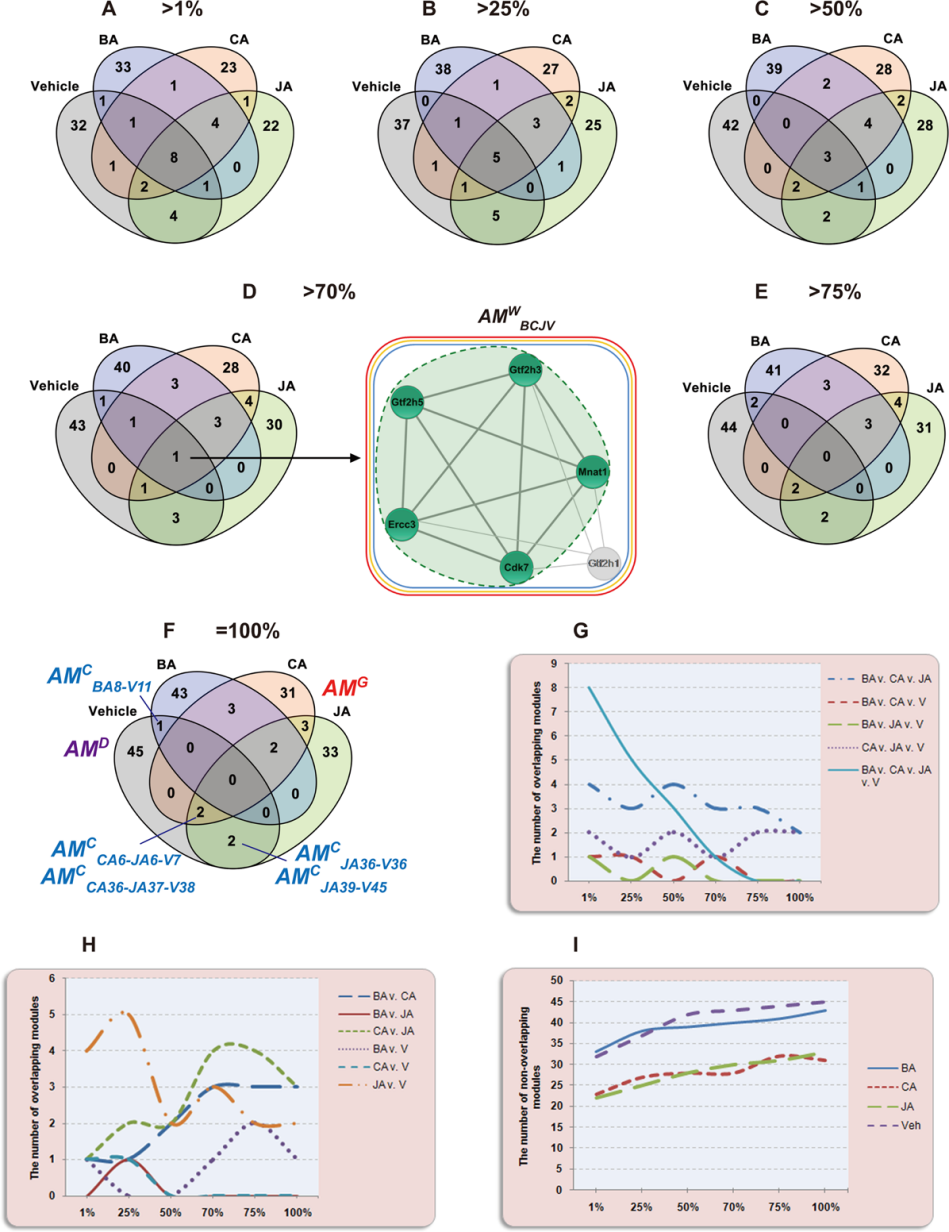
Different degrees of overlaps between modules of different treatment groups. (**A**-**F**) Six levels of similarity reflect the degree of overlaps between modules of different treatment groups, including *S*_*nef*_ >1%, >25%, >50%, >70%, >75%, and = 100%. (**D**) The *WAM* identified among the four groups. When *S*_*nef*_>70%, *AM*^*W*^_*BCJV*_ was first identified among the four groups. The green dashed area indicates the *AM* 10 in the BA group (*AM*_*BA10*_). The areas within the blue, orange and red squares represent *AM* 6 in the CA group (*AM*_*CA6*_), *AM* 6 in the JA group (*AM*_*JA6*_) and *AM* 7 in the vehicle group (*AM*_*V7*_), respectively. (**F**) Five *CAMs* were identified, namely *AM*^*C*^_*BA8*-*V11*_, *AM*^*C*^_*JA36*-*V36*_, *AM*^*C*^_*JA39*-*V45*_, *AM*^*C*^_*CA6*-*JA6*-*V7*_, and *AM*^*C*^_*CA36*-*JA37*-*V38*_. (**G** and **H**) The changing trends of the number of overlapping modules between groups. (**I**) The changing trends of the number of non-overlapping modules between groups.

By calculating the similarities between modules using SimiNEF, we found that there were different degrees of overlap between *AM*s of these groups (Fig 3E–3G, S1 Fig). We used similarity *S*_*nef*_>1%, >25%, >50%, >70%, >75%, and = 100% to define the different degrees of overlap between two *AM*s (Fig 4A–4F). Results showed that, from *S*_*nef*_>1% to *S*_*nef*_ = 100%, the numbers of overlapping modules among the vehicle, BA, CA and JA groups were 8, 5, 3, 1, 0, and 0, respectively, showing a gradually decreasing trend (Fig 4G), and the numbers of overlapping GO functions among the four groups were 192, 123, 83, 37, 0, and 0, respectively, also showing a decreasing trend. When *S*_*nef*_>75% and *S*_*nef*_ = 100%, there were no overlapping modules among the four groups (Fig 4E and 4F). With changes in similarity, the changing trends of the number of overlapping and non-overlapping modules between groups were as shown in Fig 4G–4I. Clearly, the number of non-overlapping *AM*s (special modules in each group, which we defined as new allosteric modules) gradually increased with an increase in similarity (from 1% to 100%) in all four groups (Fig 4I). Linear regression analysis showed significant associations between the number of non-overlapping *AM*s and the similarities of the vehicle (regression coefficient, 0.063; SE, 0.011; *P* = 0.005), BA (regression coefficient, 0.092; SE, 0.017; *P* = 0.006), CA (regression coefficient, 0.092; SE, 0.025; *P* = 0.02) and JA groups (regression coefficient, 0.081; SE, 0.005; *P*<0.001).

### Distribution of *AM*s, *CAM*s, *GAM*s, and *DAM*s

**Fig 5:**
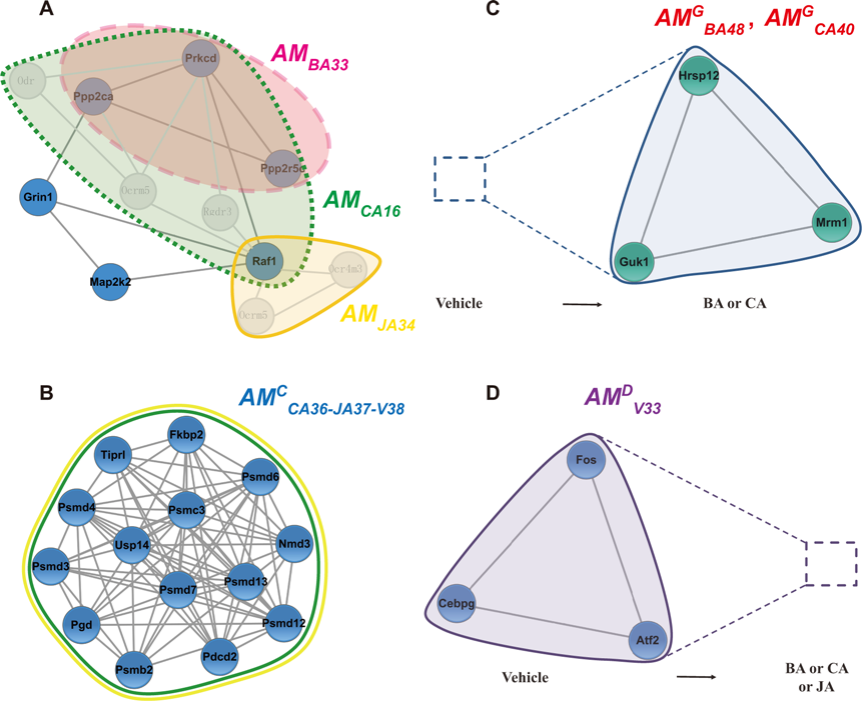
Examples of *AM*s, *CAM*s, *GAM*s, and *DAM*s. (**A**) *AM*_*BA33- CA16- JA34*_. The pink dashed area, green dotted area, area in the yellow solid line indicate *AM*_*BA33*_, *AM*_*CA16*_, *AM*_*JA34*_, respectively. Blue nodes and dark gray edges represent *AM*_*V21*_. (**B**) *AM*^*C*^_*CA36-JA37-V37*_. *AM*_*CA36*_ and *AM*_*JA37*_ (represented by green and yellow circles) were completely overlapped with *AM*_*V38*_. (**C**) *AM*^*G*^_*BA48-CA40*_. Green nodes and dark gray edges denote *AM*_*BA48*_ or *AM*_*CA40*_, and the blue dashed square indicates that the module did not appear in the vehicle group. (**D**) *AM*^*D*^_*V33*_. Blue nodes and dark gray edges denote *AM*_*V33*_, and the purple dashed square indicates that the *AM* did not appear in the BA, CA or JA group.

Compound interventions had different effects on modules in the modular networks based on ischemic mice, which resulted in topological variations or changes of compound-associated modules under different conditions. By calculating the relative overlap (i.e. similarity) between different states of the same module, we could quantitatively analyze topological structural variations of modules (based on the changes in nodes and edges primarily) in different states and explore the dynamics of allosteric modular networks. Therefore, by calculating the variation in similarity values of *AM*s under various conditions, five types of modular allostery (*AM*s, *CAM*s, *GAM*s, *DAM*s and *WAM*s) were identified in ischemic modular networks before and after compound intervention. We illustrated and defined *AM*s, *CAM*s, *GAM*s, *DAM*s and *WAM*s in Fig 1 in advance. As shown in Fig 5, we presented in detail several examples. (1) *AM*s. Most modules showed partial overlap (0<*S*_*nef*_ <100%) between various groups. In anti-ischemic modular networks, a given compound only attacked part of the disease-associated modules, namely, only parts of nodes in the disease-associated modules were affected by the compound. For example, BA acted on three nodes in *AM*_*V21*_ (Prkcd, Ppp2ca and Ppp2r5c), JA affected only one node (Raf1), and all four nodes were affected by CA (Fig 5A), indicating that the intervention of CA on *AM*_*V21*_ might be greater than that of BA or JA. (2) *CAM*s. Five *CAM*s were identified between vehicle vs. different treatment groups, e.g., *AM*^*C*^_*CA6 -JA6-V7*_ (Fig 4F), and *AM*^*C*^_*CA36-JA37-V38*_ (Fig 5B). In anti-ischemic modular networks, compounds affected the overall ischemia-associated modules, i.e., all nodes in the ischemia-associated module were affected by the compound. For instance, both CA and JA affected all nodes in *AM*^*C*^_*V38*_ (Fig 5B). *CAM*s were not significantly altered after compound intervention; therefore, these modules might not be particularly useful from a therapeutic standpoint. (3) *GAM*s. A *GAM* indicated that the module appeared after compound intervention (birth). When *S*_*nef*_ = 100%, a total of 115 *GAM*s were identified (Fig 4F). For example, *AM*^*G*^_*BA48*_ and *AM*^*G*^_*CA40*_ (Mrm1-Guk1-Hrsp12) were not found in the vehicle group but appeared in the BA and CA groups (Fig 5C); therefore, we assumed they were excitatory modules. (4) *DAM*s. A *DAM* indicated that the module in the vehicle group disappeared after compound intervention (death). When *S*_*nef*_ = 100%, 45 *DAM*s were found (Fig 4F). For example, *AM*^*D*^_*V33*_ (Fos-Cebpg-Atf2) was included in the vehicle group but disappeared in the BA, CA, or JA groups (Fig 5D); therefore, we assumed it was an inhibitory module.

### Distribution of *WAM*s and enriched KEGG pathways

When *S*_*nef*_>70%, the first overlapping module *AM*^*W*^_*BCJV*_ (including *AM*_*BA10*_, *AM*_*CA6*_, *AM*_*JA6*_ and *AM*_*V7*_) was identified among the vehicle, BA, CA and JA groups (Fig 4D).

According to the results of KEGG pathway enrichment analysis, three significantly enriched pathways (nucleotide excision repair, basal transcription factors, and viral carcinogenesis) were identified in *AM*^*W*^_*V7*_ (Table 1). The three pathways have been reported to be associated with BA in the literature, but the possible relationships between CA and nucleotide excision repair, JA and nucleotide excision repair, JA and basal transcription factors have not been reported (S9 Table). Additionally, *AM*^*W*^_*BC*_ (*S*_*nef*_>1%), *AM*^*W*^_*CJ*_ (*S*_*nef*_>1%), and *AM*^*W*^_*BJ*_ (*S*_*nef*_>25%) were identified between the BA and CA, CA and JA, BA and JA groups, respectively (Table 1). The *AM*s between the BA and JA groups held a higher similarity than those between the BA and CA or the JA and CA groups. All *WAM*s enriched two or three KEGG pathways, except *AM*^*W*^_*CJ*_. The *WAM*s might provide another approach to revealing complex pharmacological networks beyond pathways analysis.

**Table 1:** Distribution of the watershed allosteric modules and significantly enriched pathways.

Watershed allosteric modules	*S*_*nef*_	Enriched terms		Sample	Background	P-Value	Corrected	Genes IDs (Entrez Gene
		*AM* IDs	KEGG Pathways	number	number		P-Value	ID)
***AM***^***W***^_***BCJV***_ (BA vs. CA vs. JA vs. V)	>70%	*AM*_*BA10*_	Nucleotide excision repair	5	44	1.94E-11	4.10E-11	66467|209357|13872|17420|12572
			Basal transcription factors	5	47	2.74E-11	4.10E-11	66467|209357|13872|17420|12572
		*AM*_*CA6*_	Nucleotide excision repair	6	44	1.41E-13	3.22E-13	12572|13872|14884|209357|66467|17420
			Basal transcription factors	6	47	2.15E-13	3.22E-13	12572|13872|14884|209357|66467|17420
		*AM*_*JA6*_	Nucleotide excision repair	6	44	1.41E-13	3.22E-13	12572|13872|14884|209357|66467|17420
			Basal transcription factors	6	47	2.15E-13	3.22E-13	12572|13872|14884|209357|66467|17420
		*AM*_*V7*_	Nucleotide excision repair	6	45	4.15E-13	9.37E-13	12572|13872|14884|66467|209357|17420
			Basal transcription factors	6	46	4.68E-13	9.37E-13	12572|13872|14884|66467|209357|17420
			Viral carcinogenesis	2	236	0.01011	0.01348	209357|14884
***AM***^***W***^_***BC***_ (BA vs. CA)	>1%	*AM*_*BA23*_, *AM*_*CA18*_	Oxidative phosphorylation	3	142	0.00013	0.0003882	225887|226646|75406
			Parkinson's disease	3	143	0.00013	0.0003882	225887|226646|75406
			Alzheimer's disease	3	183	0.00026	0.0004351	225887|226646|75406
			Huntington's disease	3	189	0.00029	0.0004351	225887|226646|75406
			Sulfur relay system	1	10	0.00757	0.009089	69372
***AM***^***W***^_***CJ***_ (CA vs. JA)	>1%	*AM*_*CA28*_	—	—	—	—	—	—
		*AM*_*JA35*_	—	—	—	—	—	—
***AM***^***W***^_***BJ***_ (BA vs. JA)	>25%	*AM*_*BA49*_	RNA transport	4	172	1.11E-07	2.22E-07	54364|74097|117109|67676
			Ribosome biogenesis in eukaryotes	3	88	2.03E-06	2.03E-06	54364|74097|117109
		*AM*_*JA42*_	Ribosome biogenesis in eukaryotes	4	88	1.61E-08	3.23E-08	54364|74097|117109|66161
			RNA transport	4	172	2.21E-07	2.21E-07	54364|74097|117109|66161

**Notes:** *AM*^*W*^_*BCJV*_ denotes the watershed allosteric module identified among the BA, CA, JA and vehicle groups. *AM*^*W*^_*BC*_ denotes the watershed allosteric module identified between the BA and CA groups. *AM*^*W*^_*CJ*_ denotes the watershed allosteric module identified between the CA and JA groups. *AM*^*W*^_*BJ*_ denotes the watershed allosteric module identified between the BA and JA groups. The fourth column “Sample number” lists the number of input genes mapped to the particular pathway. The fifth column “Background number” lists the number of background genes mapped to the particular pathway.

“—” indicates that no KEGG pathway was enriched from the *AM*.

### Five types of *AM*s make up dynamic stereo-scroll

**Fig 6:**
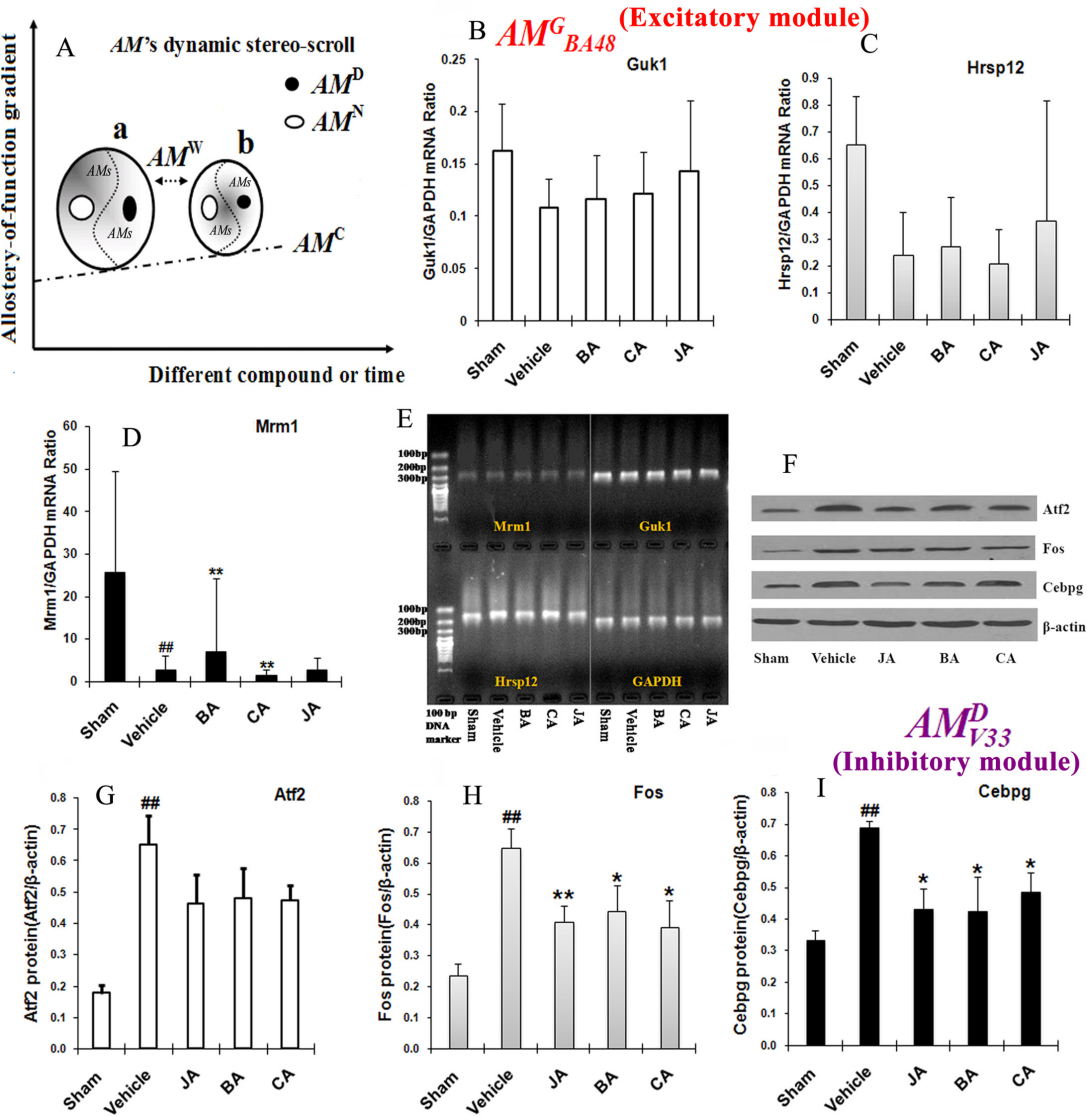
Schematic representation of *AM* dynamic stereo-scroll and biological verification for *AMGBA48* of Mrm1-Guk1-Hrsp12 and *AMDV33* of Fos-Cebpg-Atf2. (**A**) Five types of *AMs* can structure a multiple-dimensional map in diverse dynamic directions. (**B-D**) The mRNA levels of *Guk1*, *Hrsp12* and *Mrm1* among different treatment groups. ^#^*P*<0.05, ^##^*P*<0.01, compared with the sham group; ^*^P<0.05, ^**^P<0.01, compared with the vehicle group. (**E**) Representative RT-PCR bands of *Guk1*, *Hrsp12*, *Mrm1* and GAPDH. (**F**) Representative immunoblots of Atf2, Fos, Cebpg and β-actin. (**G-I**) The protein expression levels of Atf2, Fos, and Cebpg among different treatment groups. ^#^*P*<0.05, ^##^*P*<0.01, compared with the sham group; ^*^P<0.05, ^**^P<0.01, compared with the vehicle group.

To systemically reveal the complex interaction of *AM*s in modular networks, we must define, in detail, the contributions of diverse *AM*s under different treatments or at different time points. *AM*^*C*^ represents the baseline of *AMs* under different treatments or at different time points, whereas *AM*^*W*^ splits the different conditions into an allostery-of-function gradient. These two types of *AM*s provide evidence of the stability of pharmacological systems, which may be used to bridge different compounds in clinical medicine. Based on biological and pharmacological disturbances in inter-and intra-*AM*s, the numbers of *AM*^*G*^ and *AM*^*D*^ may respond to reach a dynamic balance from the pool of *AM*s. To summarize the relationships among these *AM*s, we proposed a scheme of *AM* dynamic stereo-scroll to integrate all *AM*s (Fig 6A).

### Validating functional alteration of *AM*^*G*^_*BA48*_ and *AM*^*D*^_*V33*_

We selected a *GAM* and a *DAM* from two ends of *AM*s to validate that topological variations were associated with ischemia and compound treatment. Although the mRNA levels of *Guk1* and *Hrsp12* were not significantly different among groups (*P*>0.05), the mRNA level of *Mrm1* in *AM*^*G*^_*BA48*_ was indeed significantly different among these treatment groups (Fig 6B–6E). Compared with the vehicle group, *Mrm1* mRNA was significantly up-regulated by BA and down-regulated by CA (*P*<0.01), while the associations between *Mrm1* and cerebral ischemia have not been previously reported.

Compared with the sham group, the protein expression levels of *Atf2*, *Fos*, and *Cebpg* were significantly up-regulated in the vehicle group (*P*<0.01) (Fig 6F–6I), which was also consistent with our previous findings [40,41]. *Fos* and *Cebpg* protein expression levels in *AM*^*D*^_*V33*_ were both significantly down-regulated by JA, BA and CA relative to the levels observed for the vehicle group (*P*<0.05).

## Discussion

We performed a comparative modular analysis of ischemic targeted networks based on different identification methods of *AM*s. After developing a novel similarity approach for analyzing allostery-of-function, we defined five types of *AM*s and established a dynamic stereo-scroll of different allosteric variations. This exploration offers a powerful strategy for reflecting the characteristic precision and robustness of allostery-mediated modular pattern transformation.

### SimiNEF is a novel tool for modular functional analysis

Modularity has become a fundamental concept for building disease network and drug-target networks [6]. Different modules may contribute different functions to outcome variations. Therefore, an important step of network-based approaches to disease is to identify the disease module for the pathophenotype of interest, which in turn can guide further experimental work and influence drug development [1]. Moreover, understanding *AM*s that contribute to pharmaceutics might provide novel signatures that can be used as endpoints to define disease processes or the effects of drugs under healthy or diseased conditions [6]. Although traditional pathways have served as conceptual frameworks in biological research [42], at the module layer, the synthetic biologist uses a diverse library of biological devices to assemble complex pathways that function like integrated circuits. However, the exact definition of a functional module still varies [6,43,44], and modular analysis is becoming a powerful approach for deconstrusting complex biological networks.

*AM*s display different degrees of flexibility [16]. Recently, several approaches to parameterizations and generative rules of different parameters such as path length, node, size, and modularity similarity as well as pertinent models have been developed according to a modules-within-modules perspective [45,46]. However, more fundamental tools are required to anticipate phenomena by quantitatively decomposing, reconstituting and optimizing modular structure from a topological perspective [47]. In this case, by fusing topological variation and functional alteration, SimiNEF is a powerful approach for modular functional analysis, which may help explore, in detail, the effect of modular network on the quality and stability of dynamic communities when different compounds are administered. In addition, we may also use known pathways to improve and predict unknown functions of modules [48].

### A panoramagram of *AM*s sufficiently reveals complex disease networks

The allosteric theory of signal transduction has been applied to signaling molecules as diverse as regulatory enzymes, nuclear receptors, and various classes of membrane receptors [49]. The concept of allosteric modulation in drug targeting has attracted considerable interest in recent years and may become a promising therapeutic principle [50]. Allostery appears to play a key unifying role by specifying the conformational barcode. Dysfunctional conformational barcodes in disease states can be (partially) restored to their “healthy” barcode ensemble states by allosteric drugs [2,51]. In this study, three different effective compounds acted on the same ischemic *AM* network. Modular overlaps might reveal the simultaneous involvement of nodes in multiple modules, which were determined by assigning proteins to multiple modules [52]. Not merely addressing topological overlaps (e.g., overlaps of nodes or edges), SimiNEF took into account the similarities of nodes, edges and GO functions of *AM*s altogether to reveal the fusing alteration of topological and functional similarities between *AM*s. Different effective compounds attacked diverse nodes in the same ischemic *AM*s network, and five types of modular allostery (*AM*s, *CAM*s, *GAM*s, and *DAM*s and *WAM*s) were identified, which reflected the structural and functional diversity of *AM*s before and after compound intervention. Because allosteric propagation can occur via large cellular assemblies over large distances [51] or within the protein matrix to eventually reach the substrate site [53], in our study most *AM*s were partially overlapped (0< *S*_*nef*_ <100%) and more *GAM*s were observed than *DAM*s, whereas only five *CAM*s were identified in the vehicle group. Thus, allostery could help explain how different compounds perturbed the ischemic modular network. For example, as shown in Fig 5A, *AM*_*BA33*_, *AM*_*CA16*_, and *AM*_*JA34*_ all affected *AM*_*V21*_, which was enriched for the function “phosphate metabolic process”; then, BA, CA, and JA all intervened in the function, which reflected the similarity of their pharmacological mechanisms. Specifically, however, BA, CA, and JA affected different genes or proteins in *AM*_*V21*_, e.g., the number of genes affected by CA was the sum of that of BA and JA, which reflected the diversity of their pharmacological mechanisms. Moreover, based on different levels of similarity, *WAM*s were identified among the vehicle, BA, CA and JA groups, as well as between any two of the compound-treated groups. Such a *WAM* contributed to reveal the demarcation point of common and diverse pharmacological mechanisms between different effective compounds.

Indirectly using specific inter-protein network pathways can affect the pharmacological target protein [9,51]. Thus, drugs do not target the actual disease-associated proteins but bind to their 3rd or 4th neighbors. The distance between drug targets and disease-associated proteins is regarded as a sign of palliative drug action [4,9]. In this study, we suppose that BA, CA or JA might indirectly and specifically affect pharmacological targets or key proteins by targeting “by-stander” proteins (e.g., Cebpg, *Mrm1*). Our findings indicate that Cebpg protein expression was down-regulated by JA, BA and CA. Although the association between Cebpg and CA has not been previously reported, it was demonstrated that chenodeoxycholic acid (CDCA), one of the primary bile acids, induced antioxidant and xenobiotic-metabolizing enzymes by activating C/EBPβthrough phosphorylation [54]. With the assurance of different allosteric modulators of diverse functions and dynamics [55,56], an allosteric modulated approach may be achieved from disease molecular insights into therapeutic perspectives [50]. Although this is the first time *AM*s have been analyzed, the allostery of targeted systems is anticipated to provide effective solutions to challenges that include variations in nodes (^N^*AM*), edges (^E^*AM*) and related functions. Our ability to reveal, in detail, the transformational information from disease network systems and to process information inside *AM*s is critical to advancing the topological alteration and functional complexity with which we can engineer, predict, and probe pharmacological systems. Thus, we developed a novel paradigm of assembling *AM*s that allows for the quantitative analysis of gradient mechanisms of targeted network variations.

## Supporting Information

S1 Fig The degree of overlap between modules of different groups. (PDF)

S1 Table Topological attributes of global networks in different groups. (DOCX)

S2 Table Affinity propagation (AP) results for all parameters tested. (DOCX)

S3 Table MCL results for all parameters tested. (DOCX)

S4 Table MCODE results for all parameters tested. (DOCX)

S5 Table Functional modules identified by MCODE in different groups. (DOCX)

S6 Table 218 significantly enriched GO biological processes. (DOCX)

S7 Table Overlapping and non-overlapping GO biological processes. (DOCX)

S8 Table Overlapping and non-overlapping KEGG pathways. (DOCX)

S9 Table Relationship between compounds and KEGG pathways in the watershed allosteric modules supported by previous literature. (DOCX)

## Abbreviations

BA: baicalin
CA: cholic acid
JA: jasminoidin
*AM*s: allosteric modules
WAM: watershed allosteric module
*CAM*s: conserved allosteric modules
*GAM*s: generated allosteric modules
*DAM*s: disappeared allosteric modules
