# Supplementary material for: Quantitative Determination of Flexible Pharmacological Mechanisms Based On Topological Variation in Mice Anti-Ischemic Modular Networks
Source: PLoS One. 2016 Jul 6;11(7):e0158379. doi: 10.1371/journal.pone.0158379 (PMC4934924; doi:10.1371/journal.pone.0158379)
Supplement: S1 Fig — (PDF) [file pone.0158379.s001.pdf]

S1 Fig. The degree of overlap between modules of different groups. Related to Fig 3. Modular overlaps between BA and CA, BA and JA, BA and vehicle, CA and JA, CA and vehicle, JA and vehicle groups. Each column of numbers indicates each number of modules in each group. For example, if the overlap of nodes, edges or GO functions (any one of them) between two modules is greater than 1%, 10%, 25%, 50%, 60%, 75%, 90%, 95% or 99%, these two modules are connected. The blue dashed line indicates that the overlap of nodes between two modules is greater than 1%, 10%, 25%, 50%, 60%, 75%, 90%, 95% or 99%. The pink dotted line indicates that the overlap of edges between two modules is greater than 1%, 10%, 25%, 50%, 60%, 75%, 90%, 95% or 99%. The red solid line indicates that the overlap of GO functions between two modules is greater than 1%, 10%, 25%, 50%, 60%, 75%, 90%, 95% or 99%.

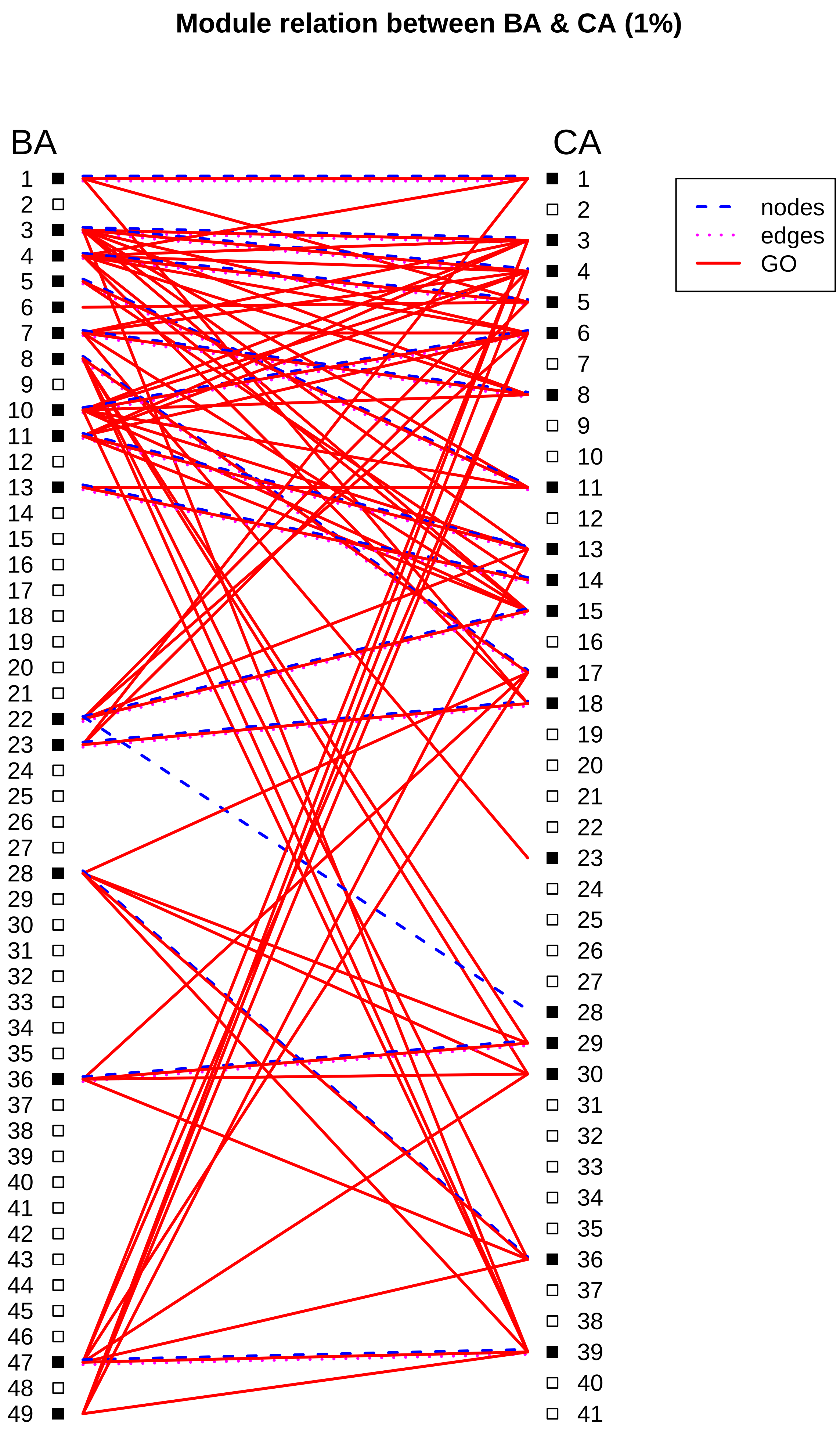
Module relation between BA & CA (1%)
BA
CA
nodes
edges
GO
1
2
3
4
5
6
7
8
9
10
11
12
13
14
15
16
17
18
19
20
21
22
23
24
25
26
27
28
29
30
31
32
33
34
35
36
37
38
39
40
41
42
43
44
45
46
47
48
49

Module relation between BA & JA (1%)

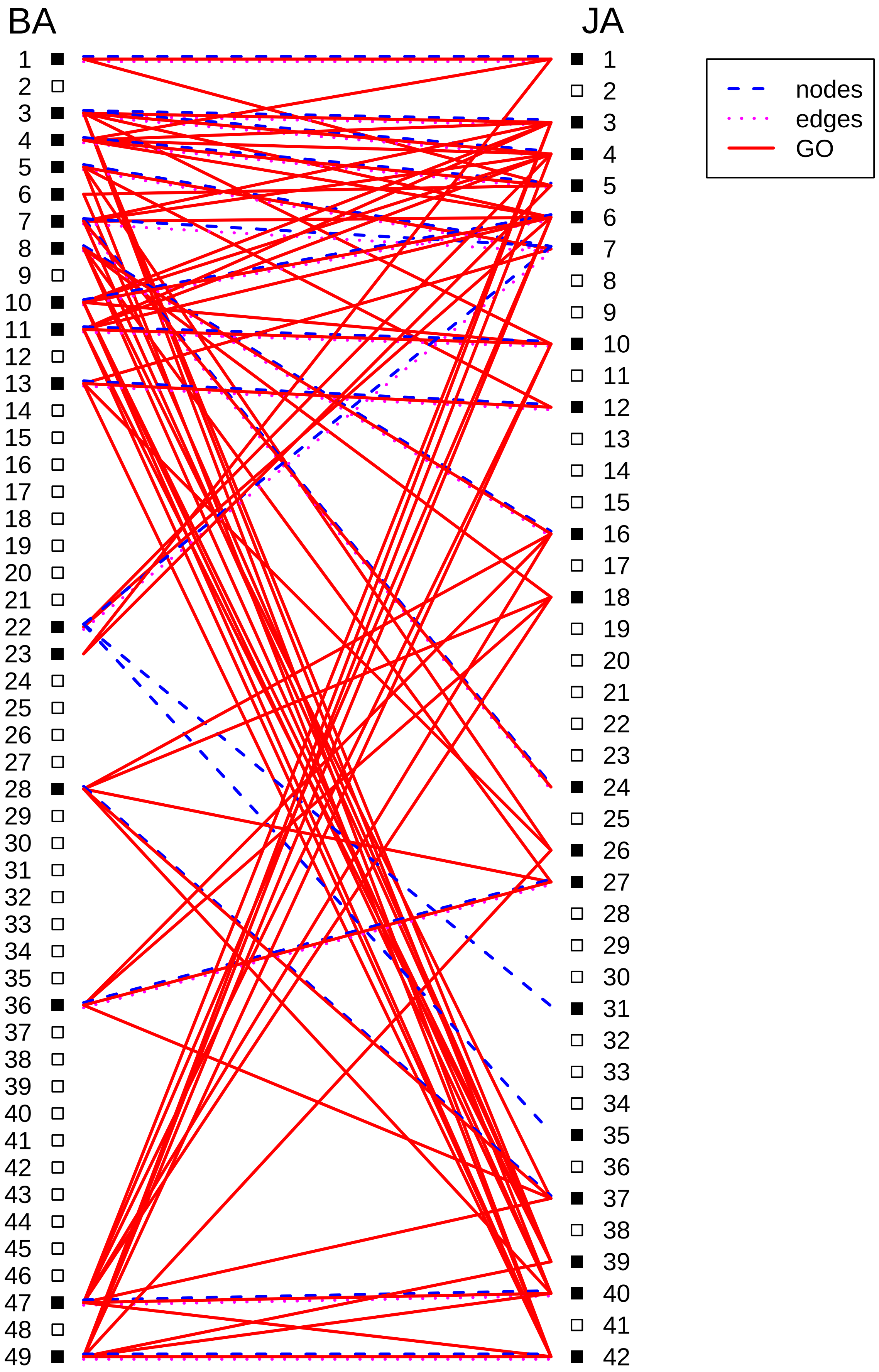

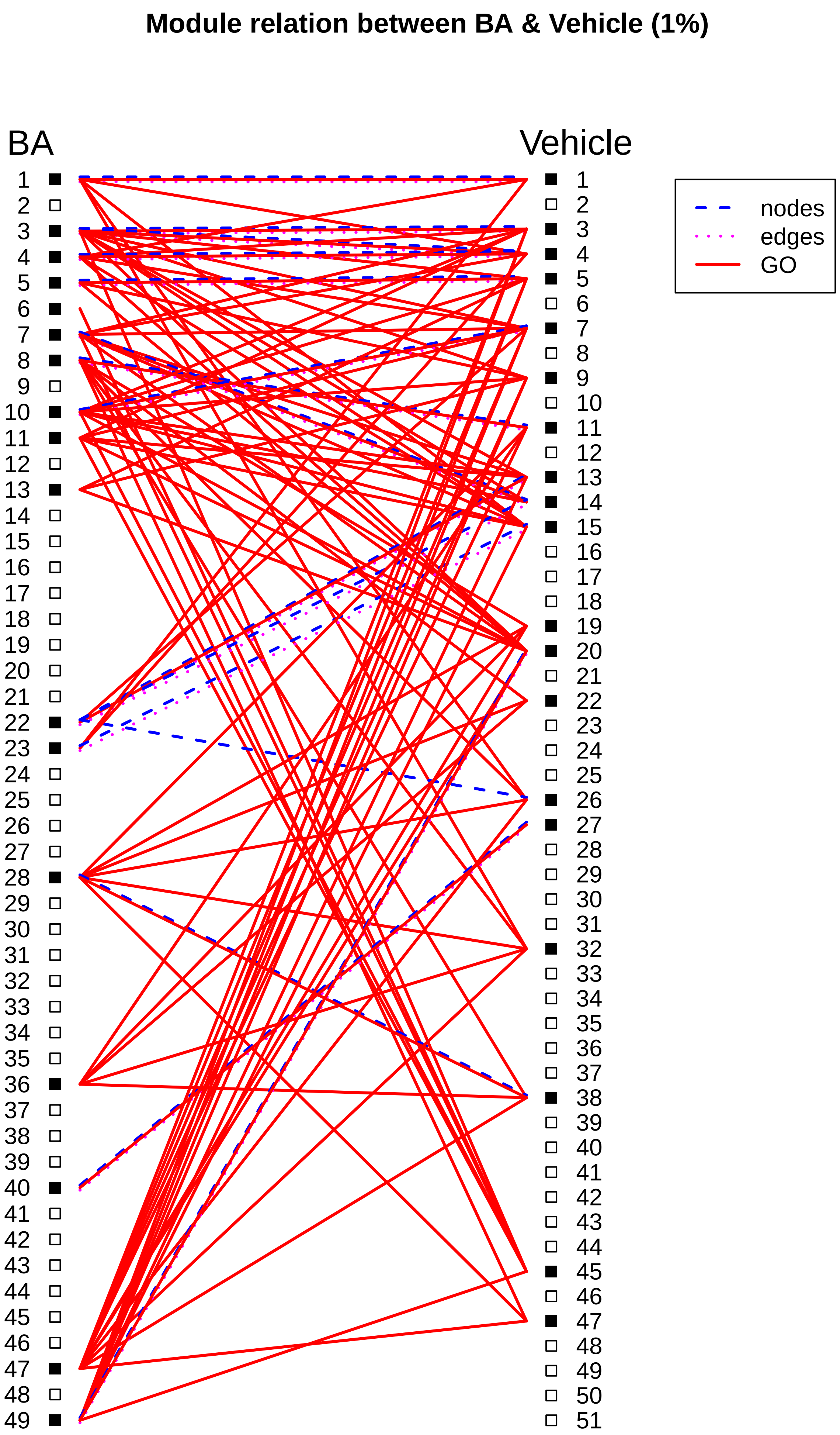
Module relation between BA & Vehicle (1%)
BA
Vehicle
nodes
edges
GO
1
2
3
4
5
6
7
8
9
10
11
12
13
14
15
16
17
18
19
20
21
22
23
24
25
26
27
28
29
30
31
32
33
34
35
36
37
38
39
40
41
42
43
44
45
46
47
48
49
1
2
3
4
5
6
7
8
9
10
11
12
13
14
15
16
17
18
19
20
21
22
23
24
25
26
27
28
29
30
31
32
33
34
35
36
37
38
39
40
41
42
43
44
45
46
47
48
49
50
51

Module relation between CA & JA (1%)

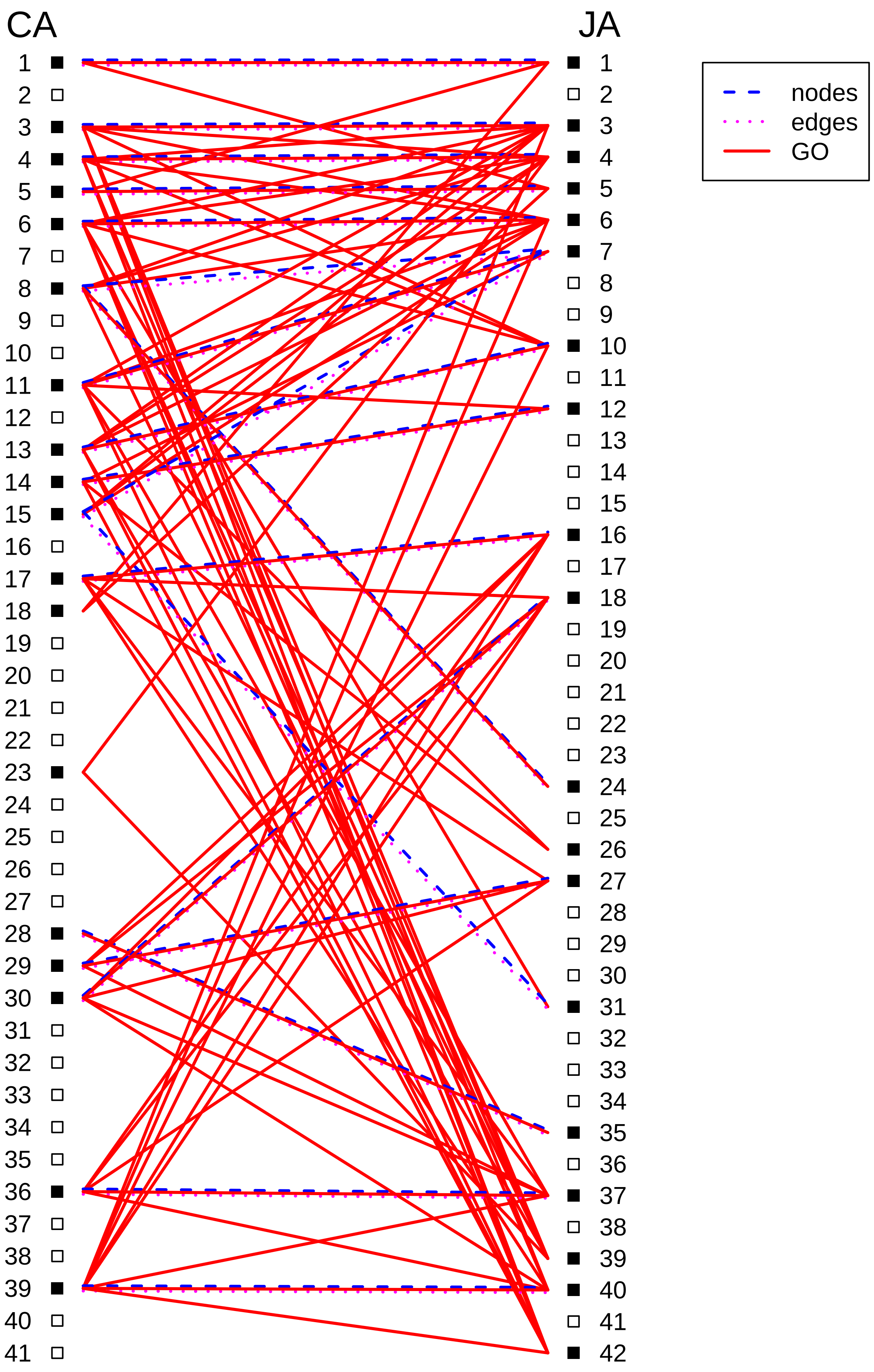

Module relation between CA & Vehicle (1%)

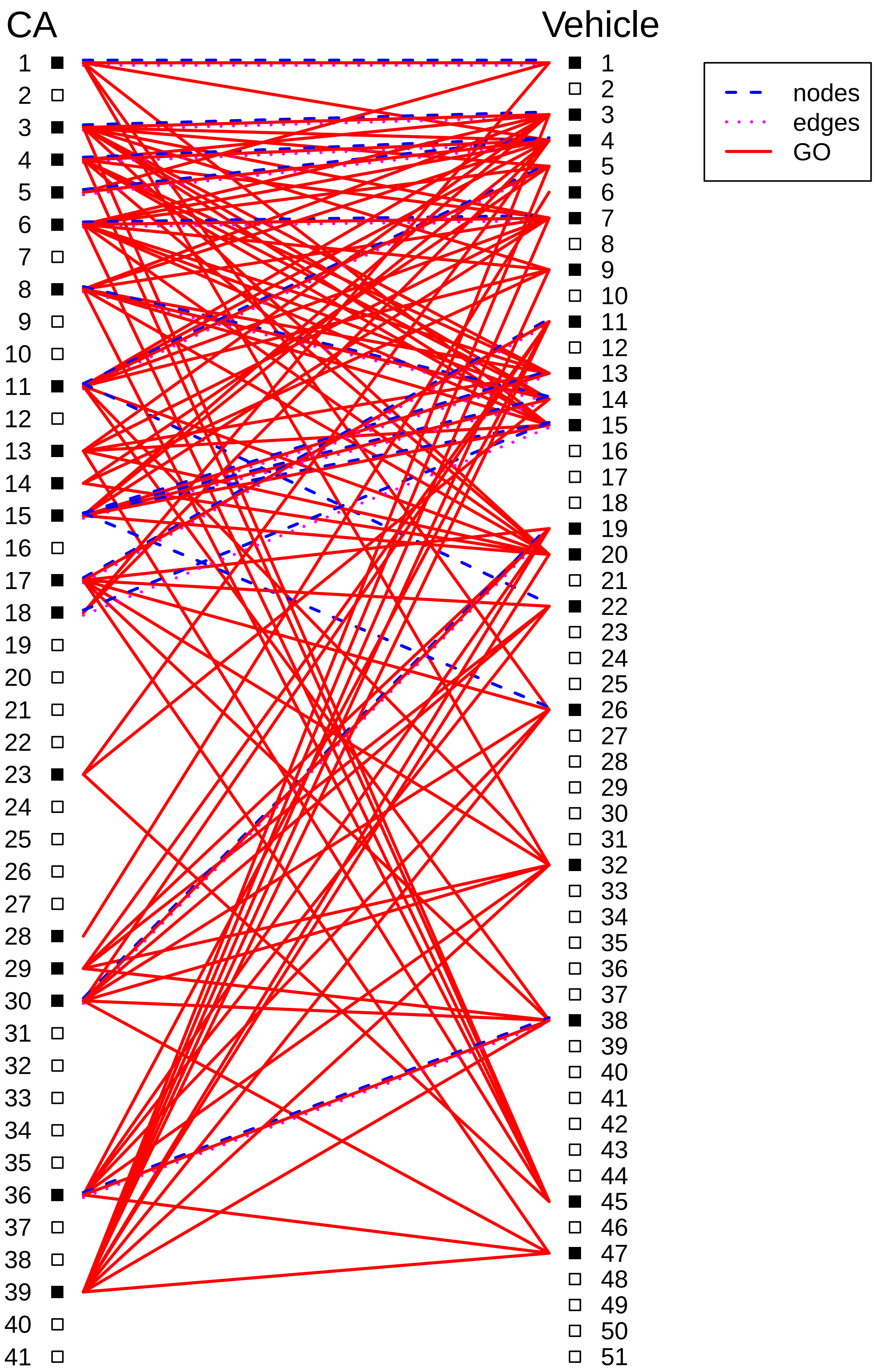

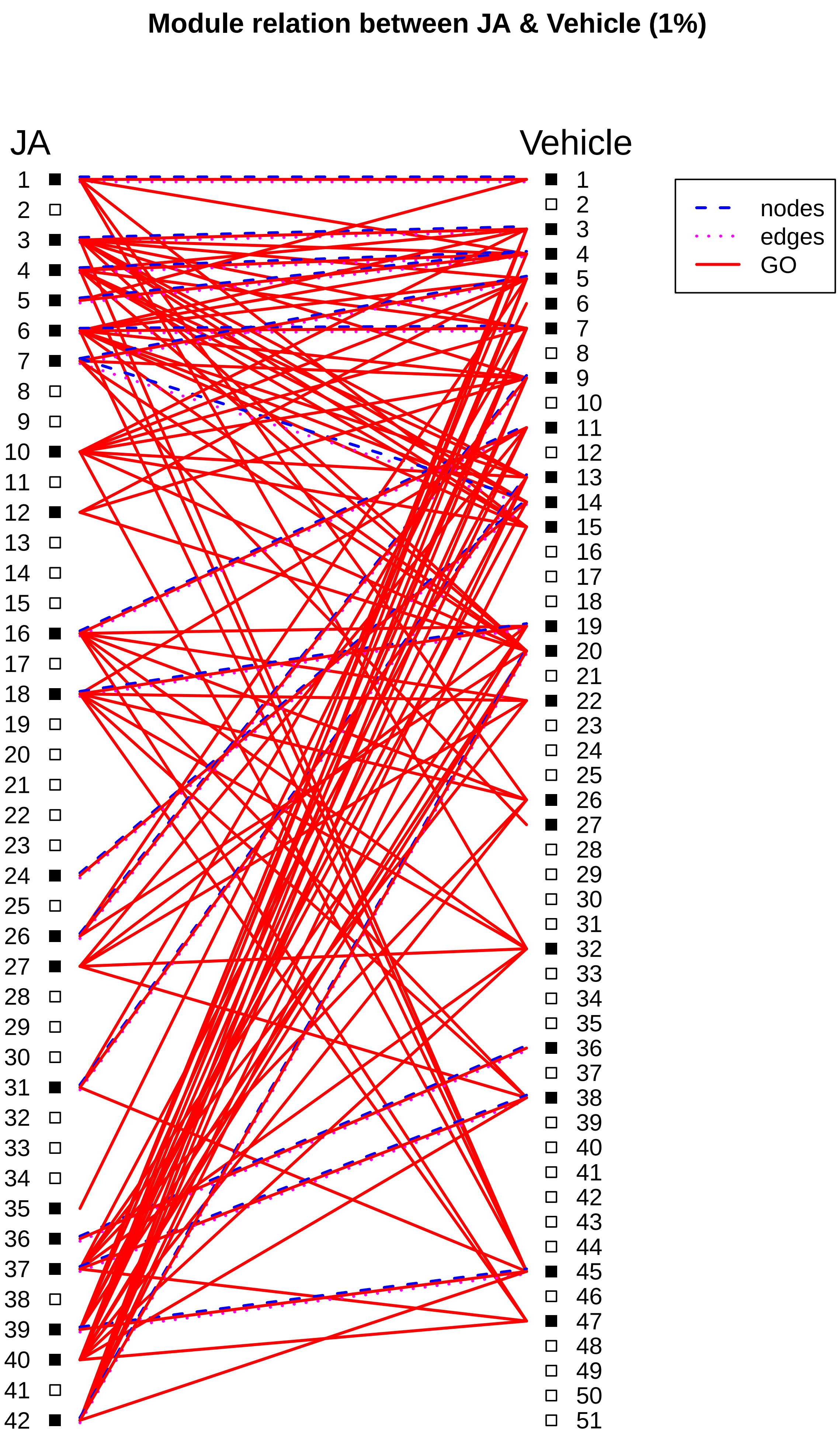
Module relation between JA & Vehicle (1%)
JA
Vehicle
nodes
edges
GO
1
2
3
4
5
6
7
8
9
10
11
12
13
14
15
16
17
18
19
20
21
22
23
24
25
26
27
28
29
30
31
32
33
34
35
36
37
38
39
40
41
42
43
44
45
46
47
48
49
50
51

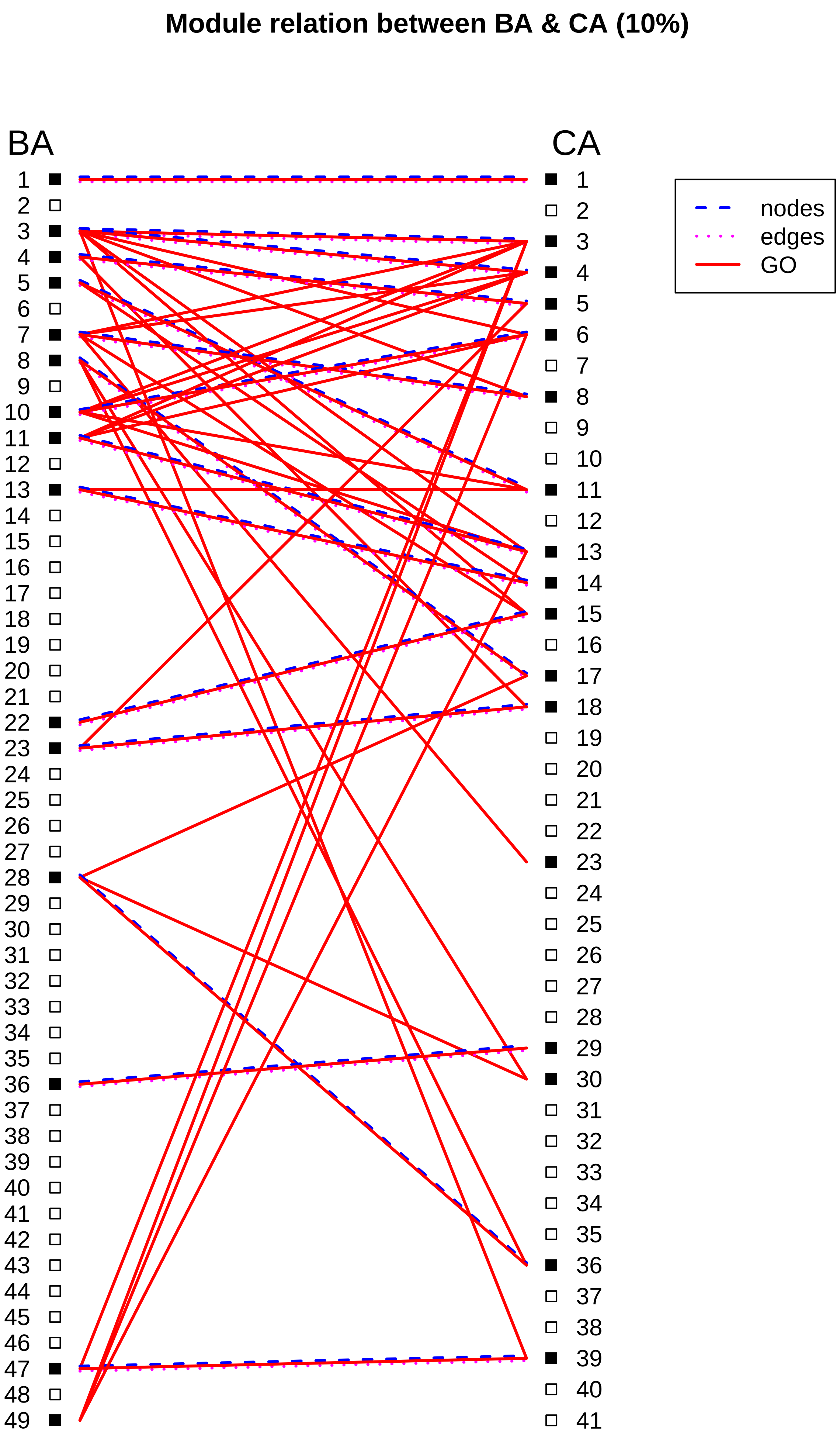
Module relation between BA & CA (10%)
BA
CA
1
2
3
4
5
6
7
8
9
10
11
12
13
14
15
16
17
18
19
20
21
22
23
24
25
26
27
28
29
30
31
32
33
34
35
36
37
38
39
40
41
42
43
44
45
46
47
48
49
nodes
edges
GO

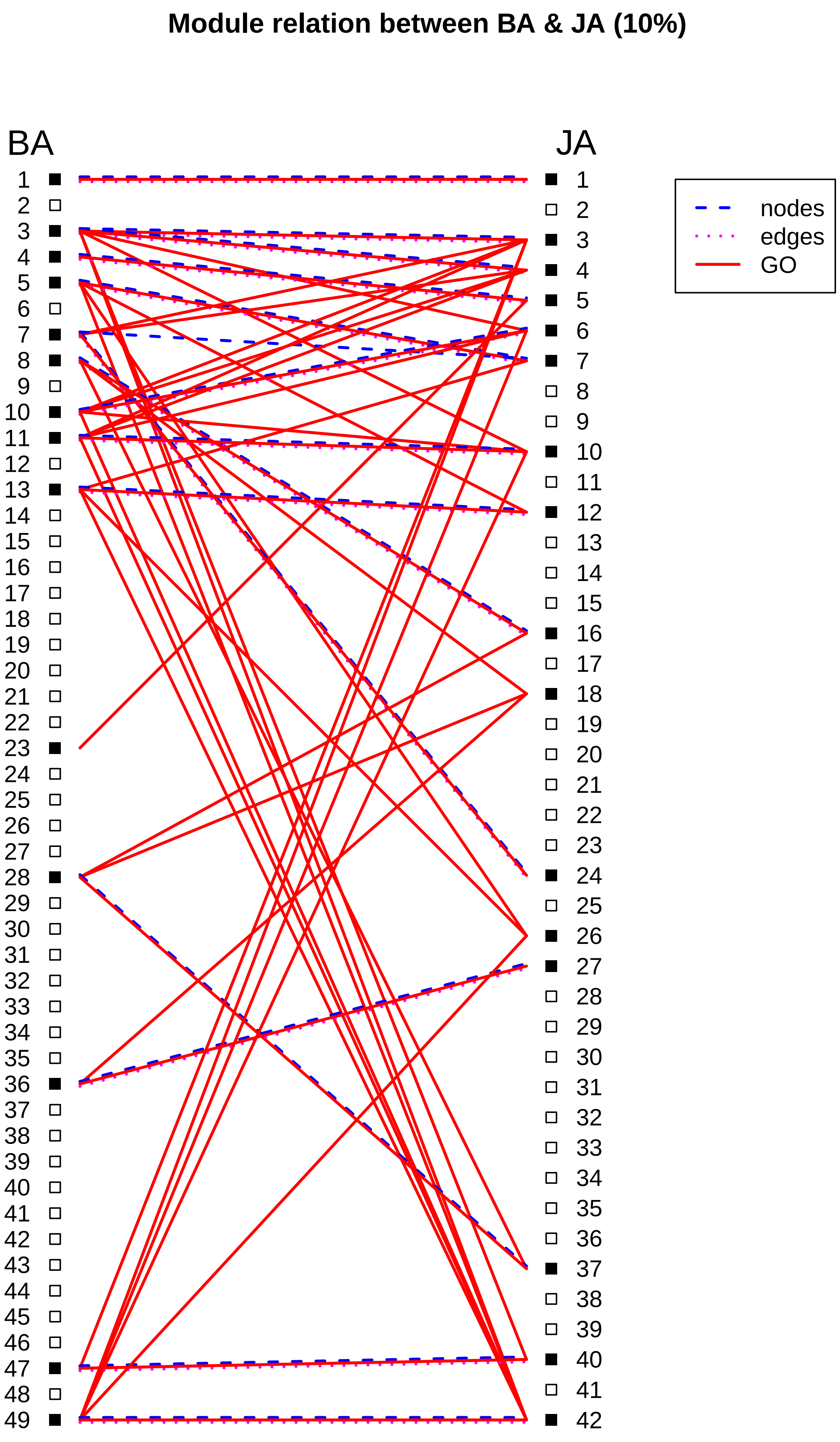

Module relation between BA & JA (10%)
BA
JA
1
2
3
4
5
6
7
8
9
10
11
12
13
14
15
16
17
18
19
20
21
22
23
24
25
26
27
28
29
30
31
32
33
34
35
36
37
38
39
40
41
42
43
44
45
46
47
48
49
nodes
edges
GO

Module relation between BA & Vehicle (10%)

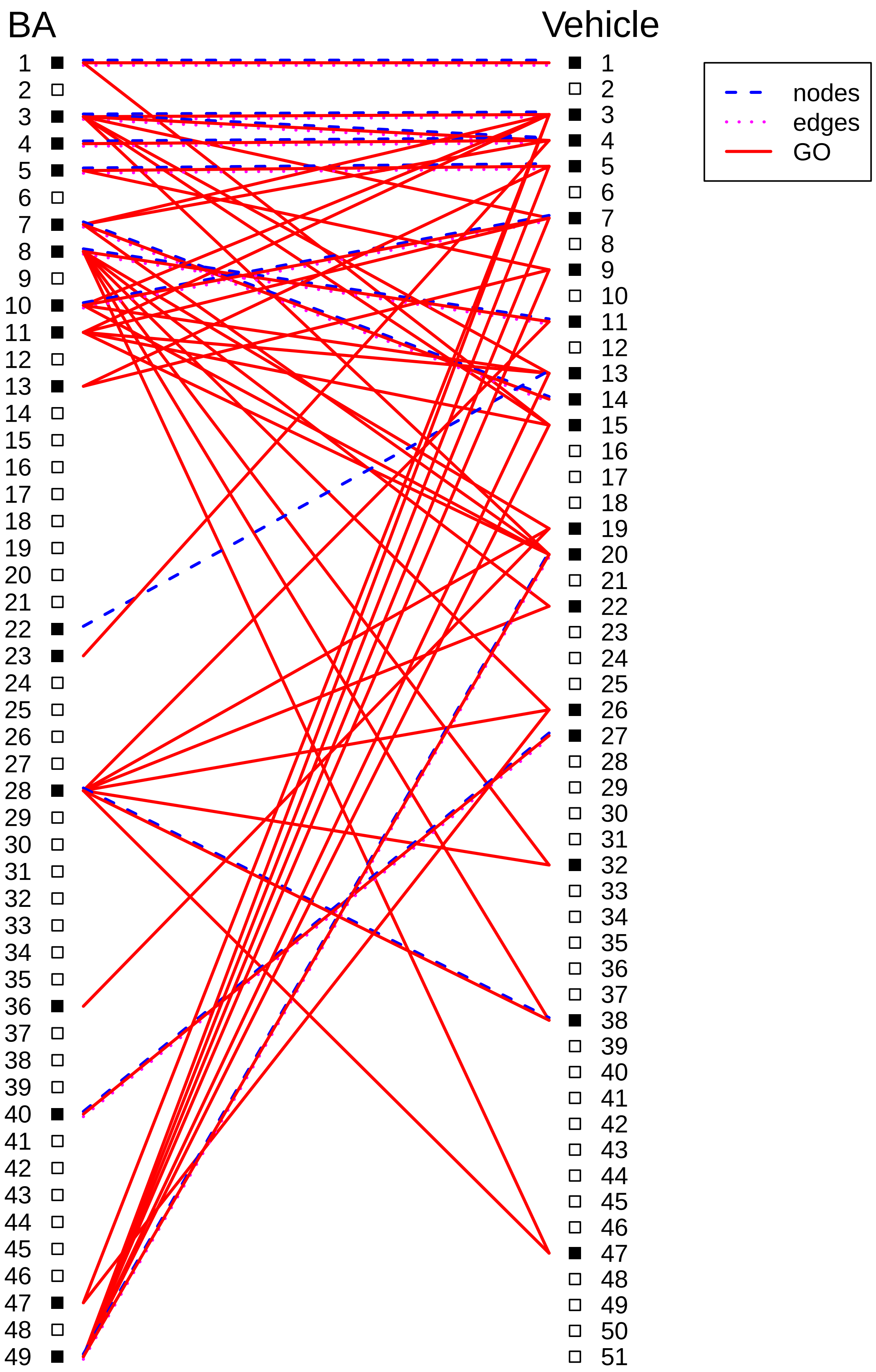

Module relation between CA & JA (10%)

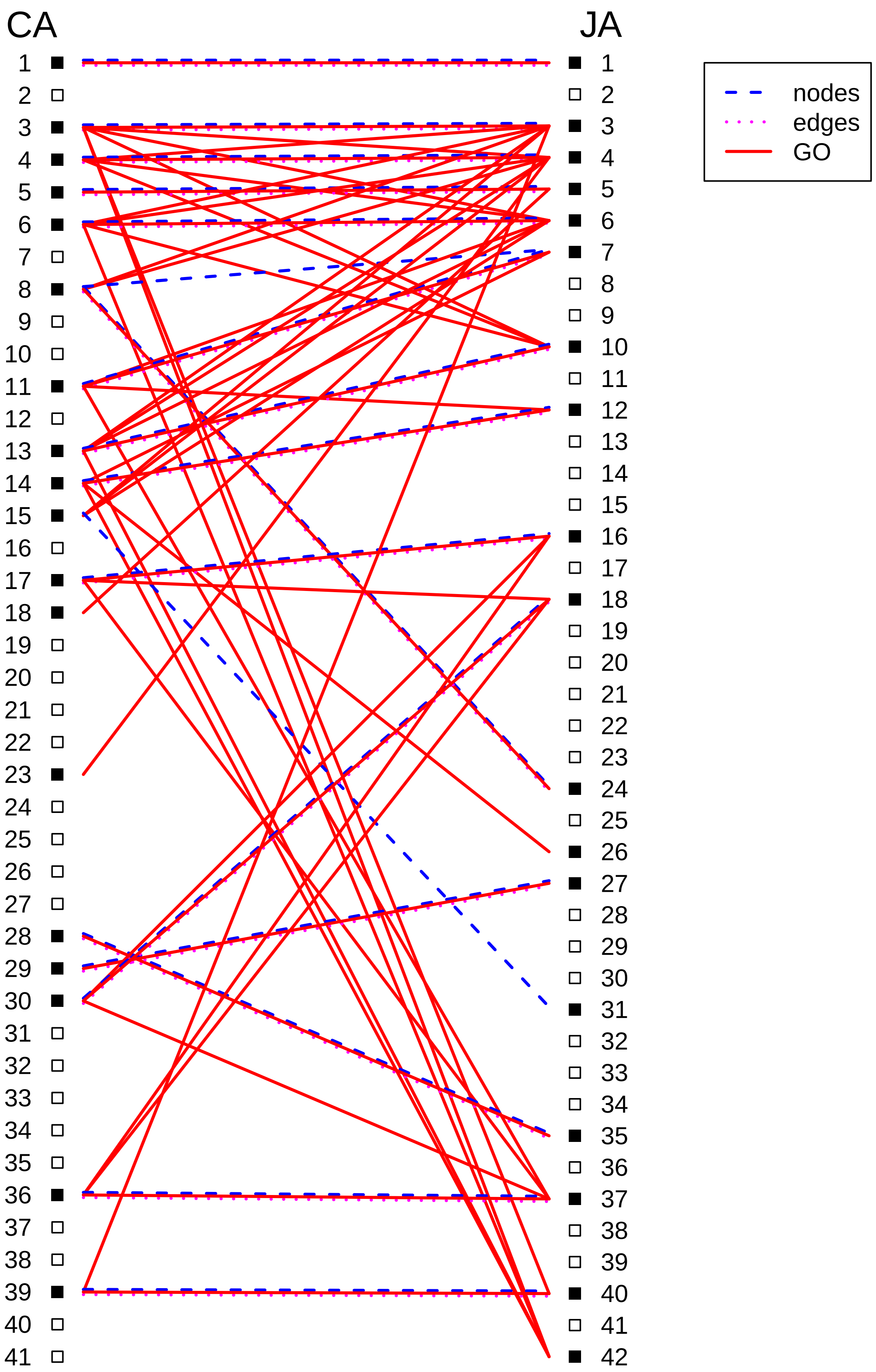

Module relation between CA & Vehicle (10%)

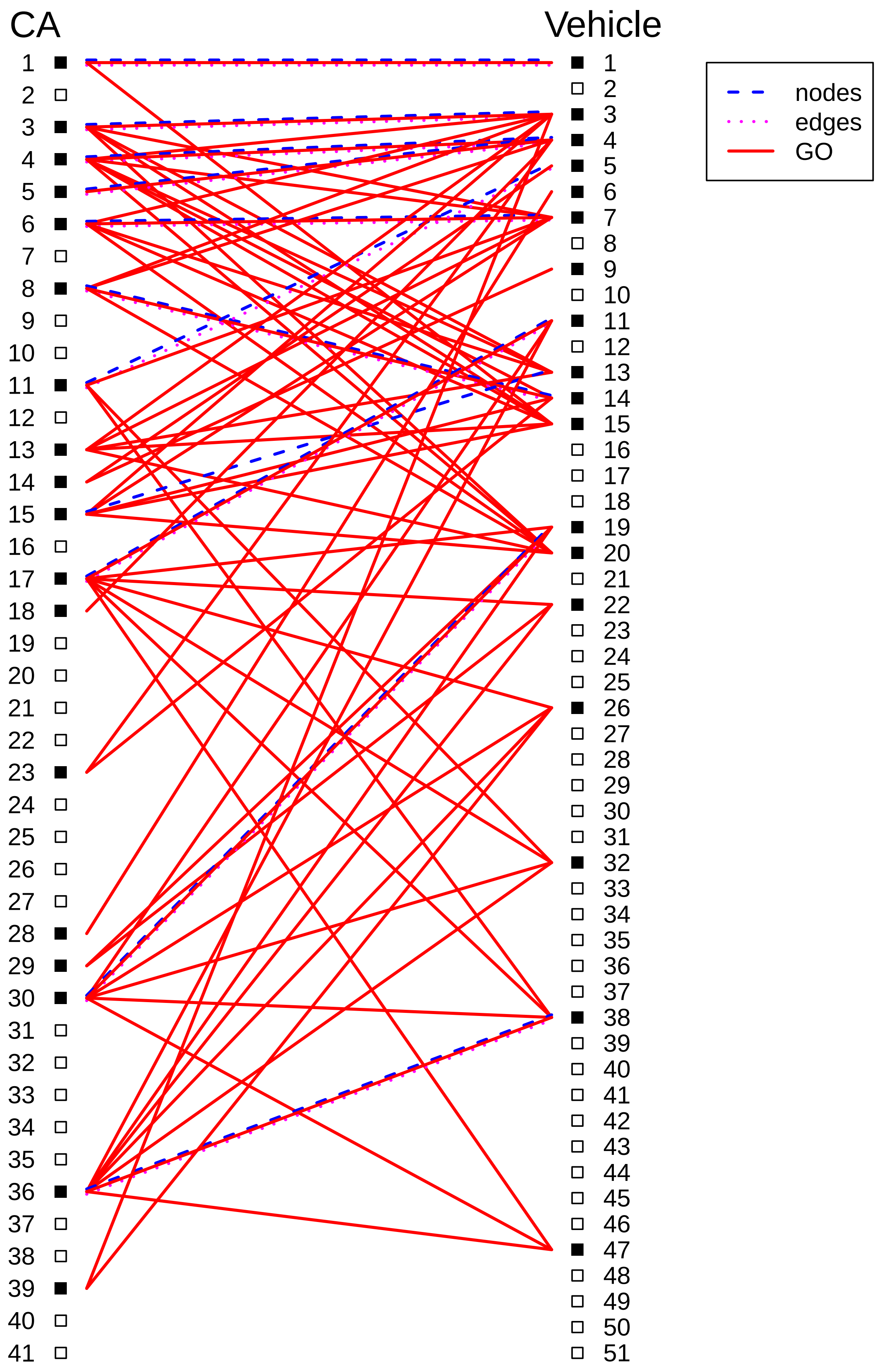

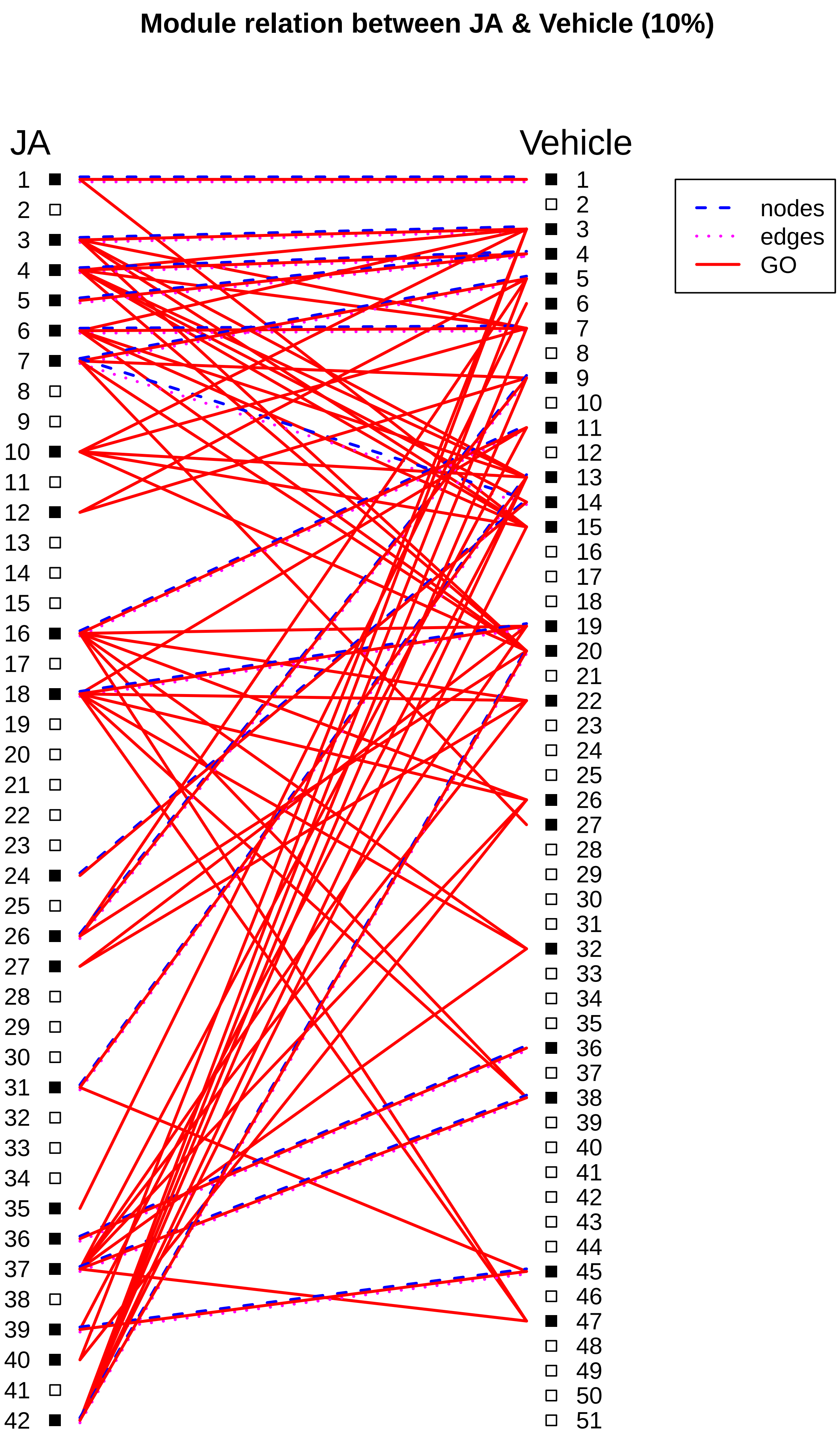

Module relation between JA & Vehicle (10%)
JA
Vehicle
nodes
edges
GO

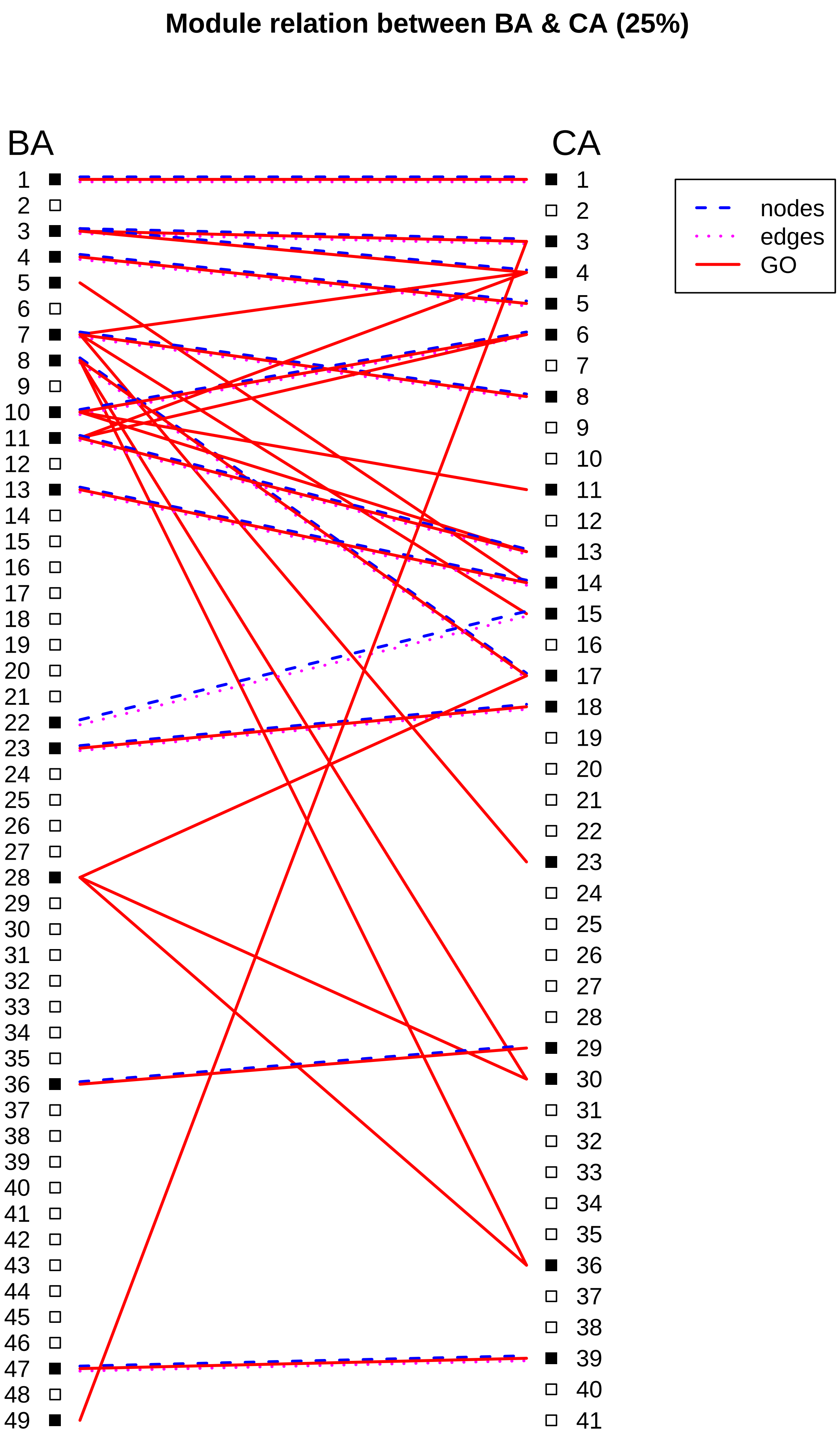
Module relation between BA & CA (25%)
BA
CA
nodes
edges
GO

Module relation between BA & JA (25%)

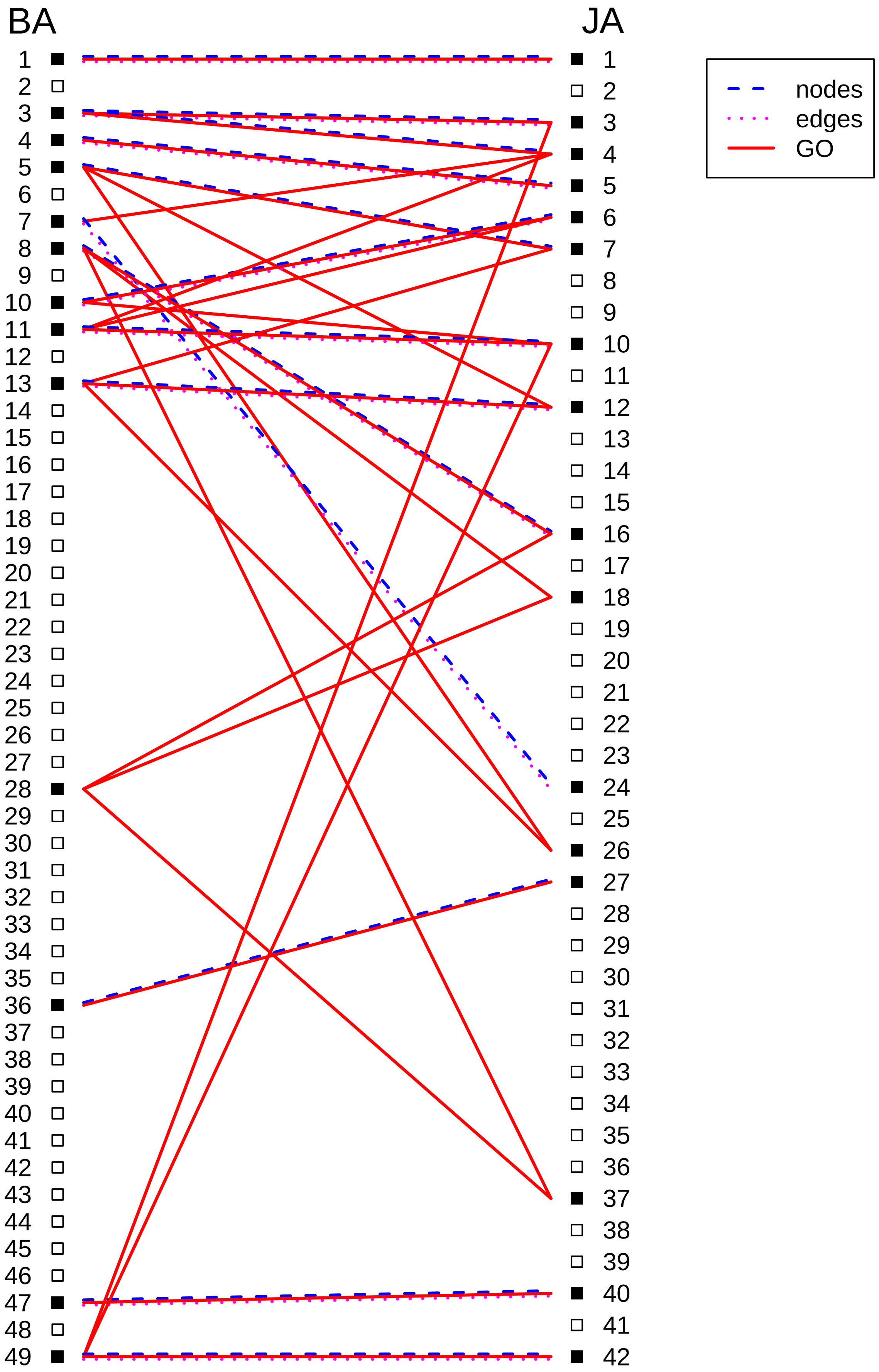

Module relation between BA & Vehicle (25%)

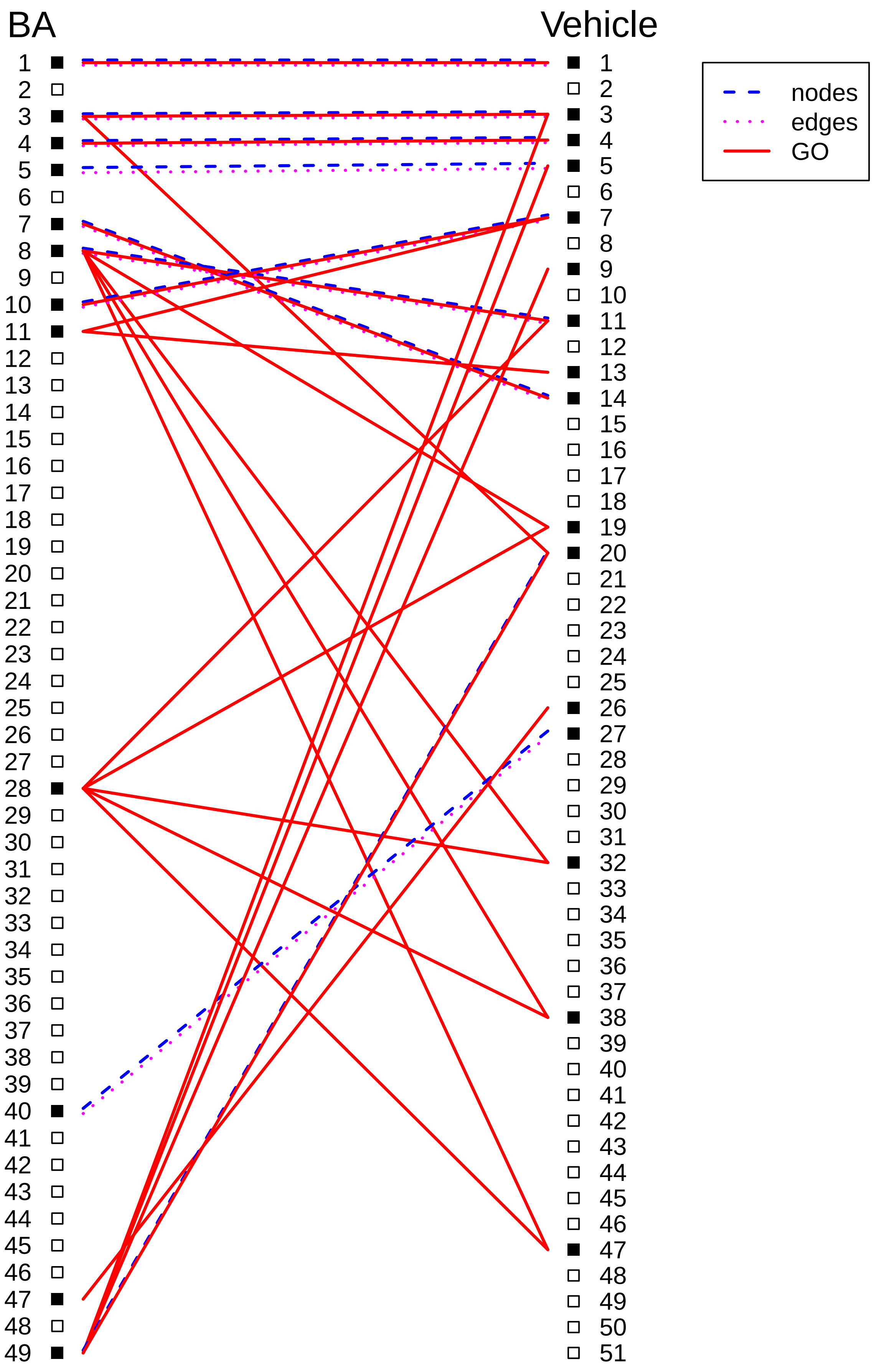

Module relation between CA & JA (25%)

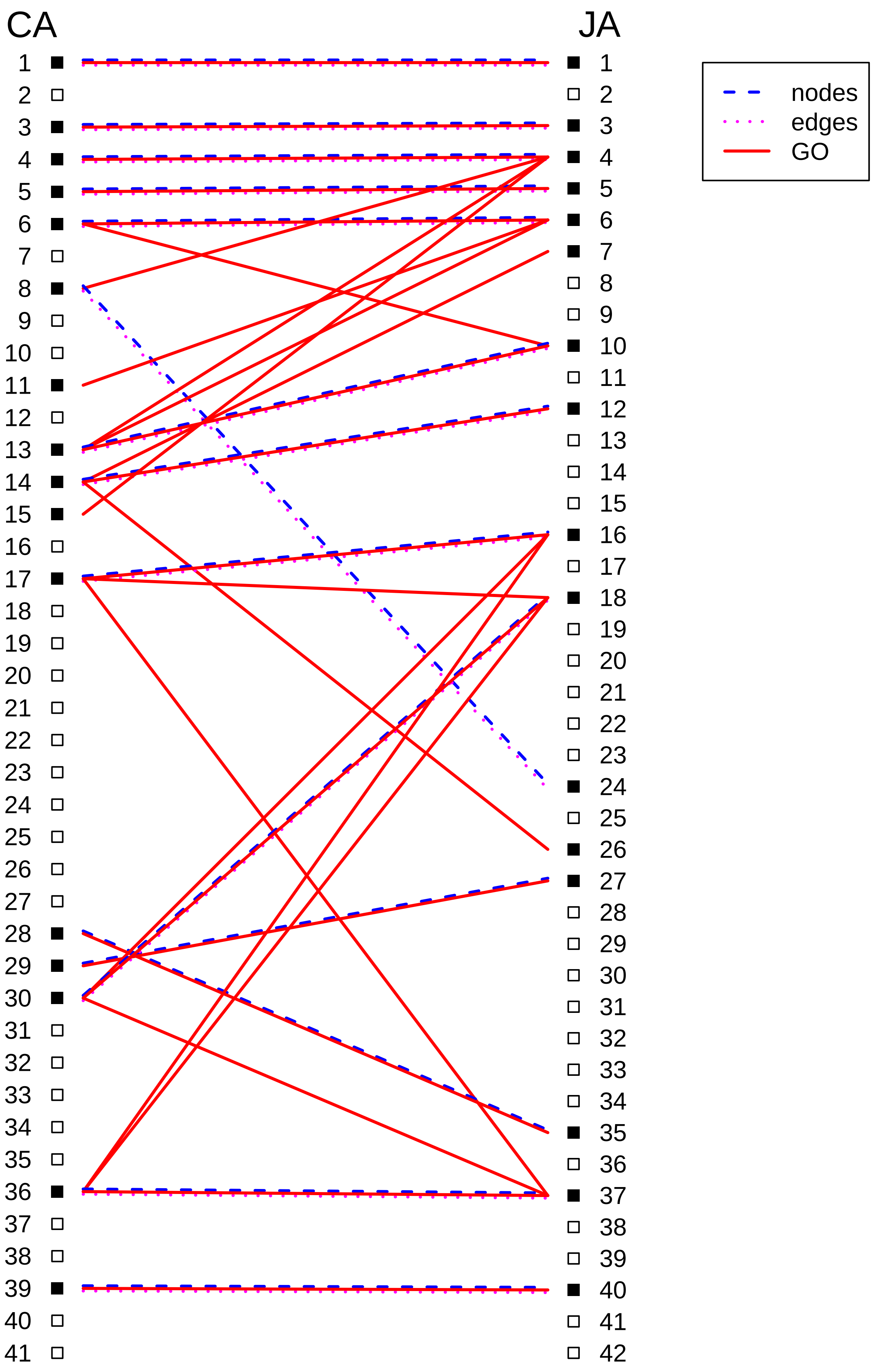

Module relation between CA & Vehicle (25%)

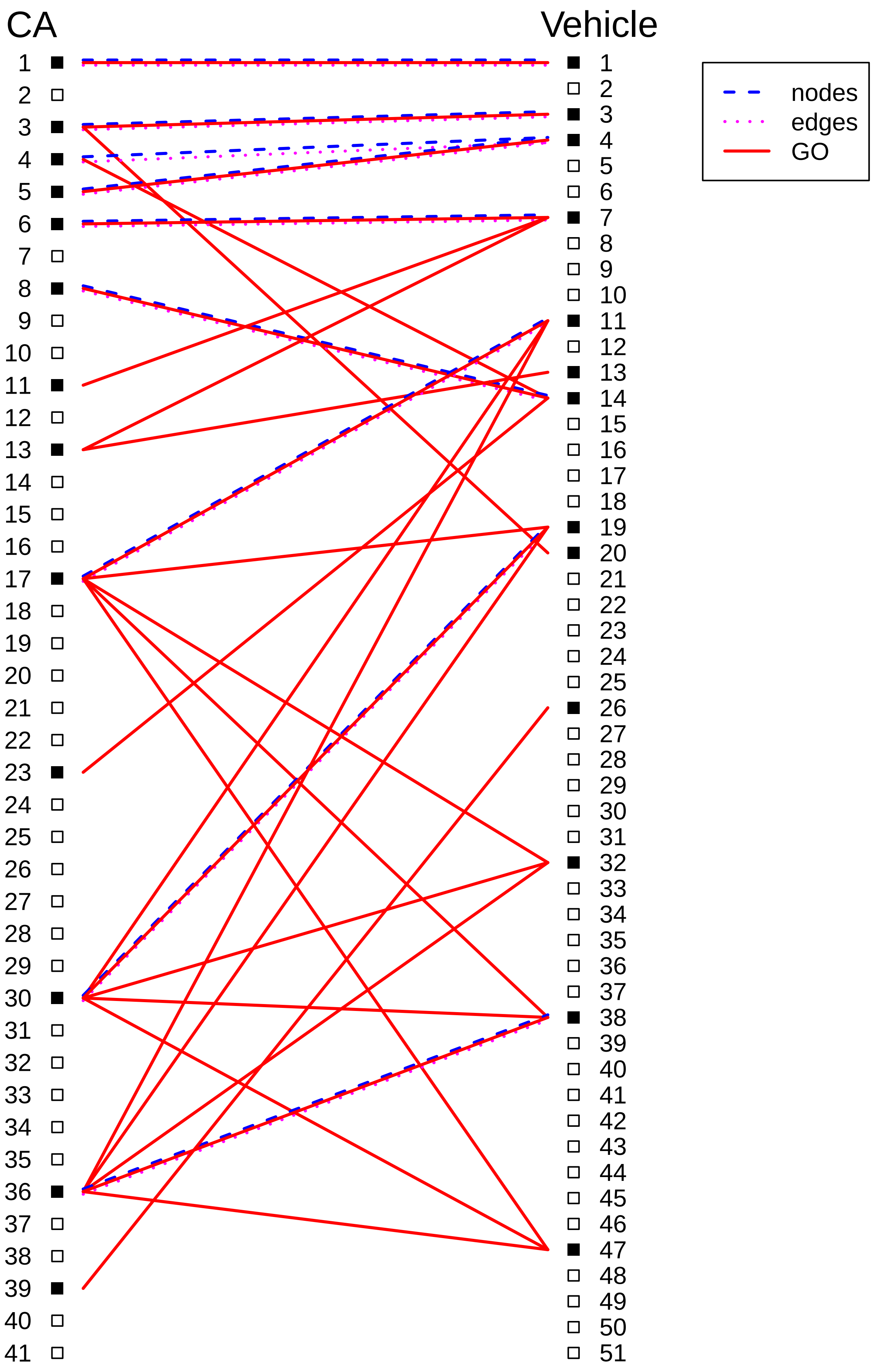

Module relation between JA & Vehicle (25%)

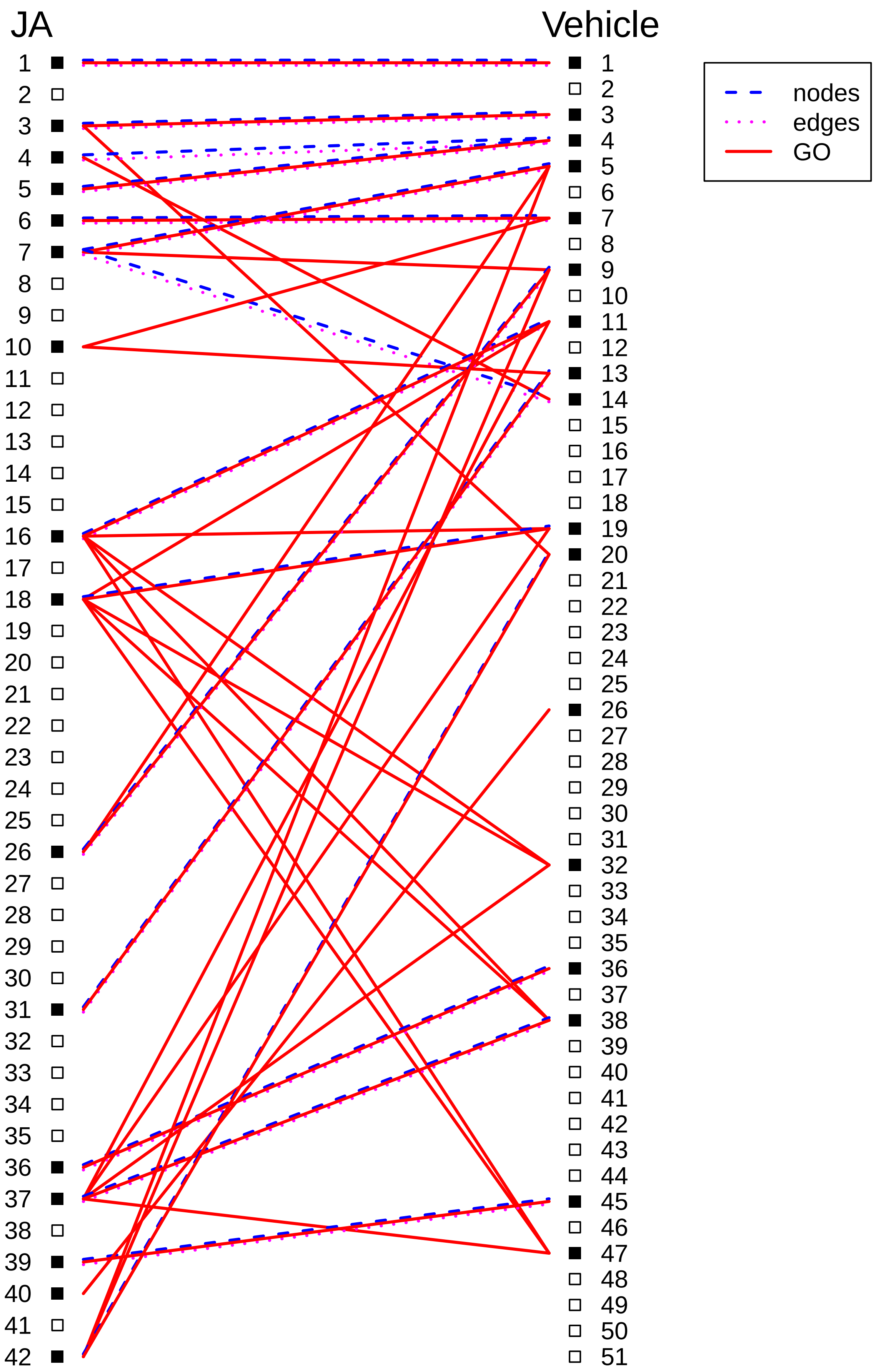

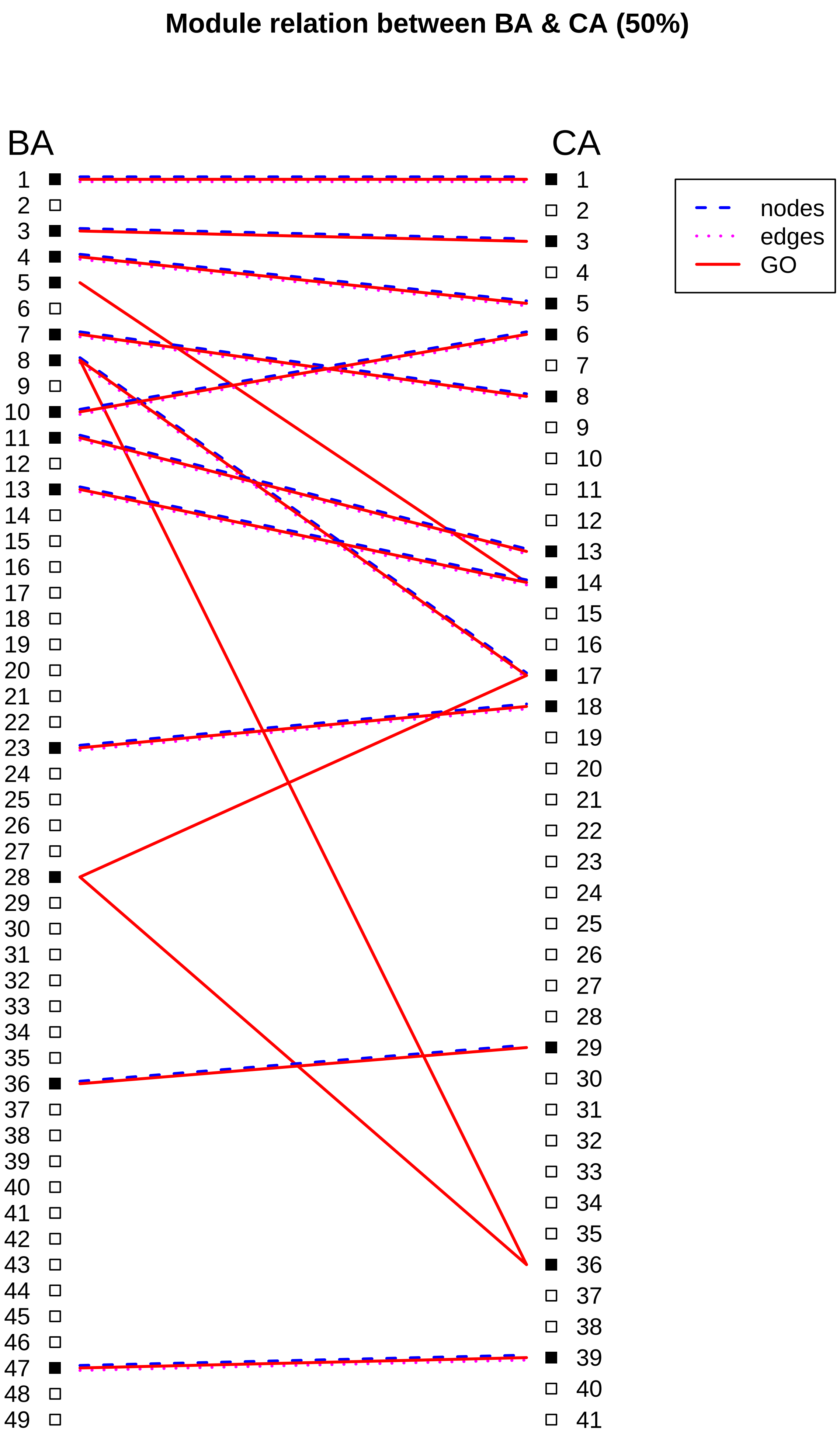
Module relation between BA & CA (50%)
BA
CA
1
2
3
4
5
6
7
8
9
10
11
12
13
14
15
16
17
18
19
20
21
22
23
24
25
26
27
28
29
30
31
32
33
34
35
36
37
38
39
40
41
42
43
44
45
46
47
48
49
nodes
edges
GO

Module relation between BA & JA (50%)

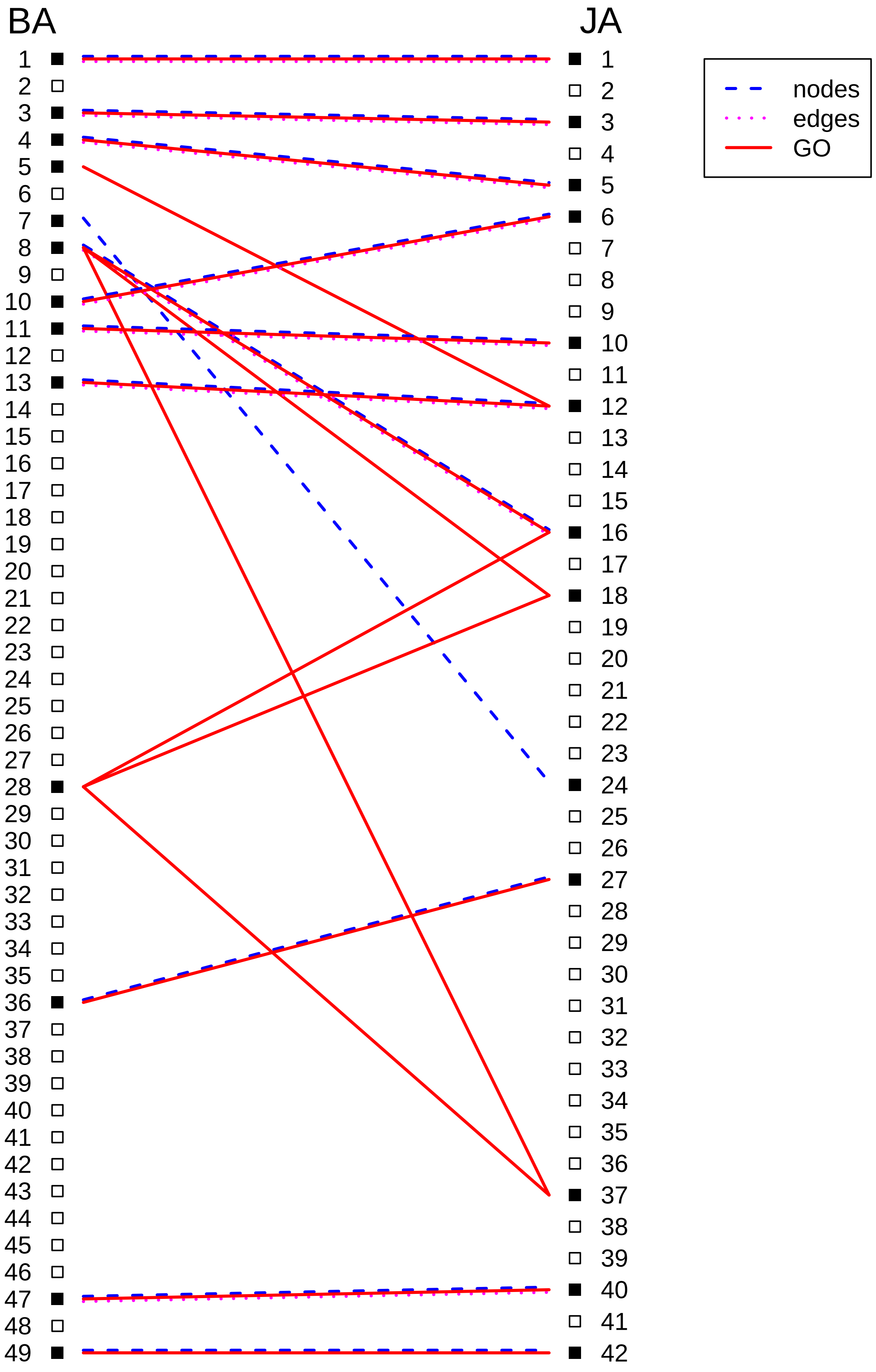

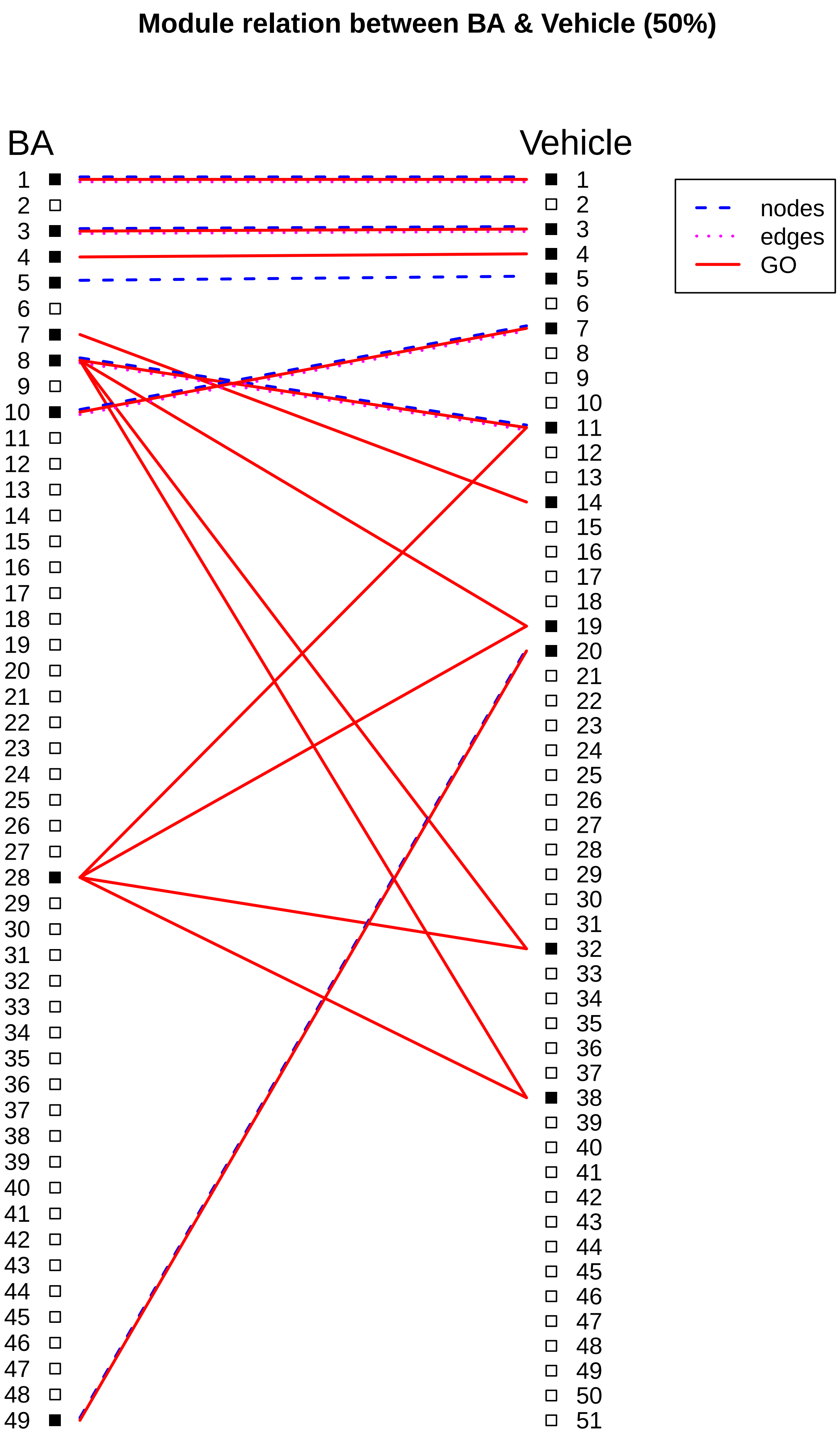

Module relation between BA & Vehicle (50%)
BA
Vehicle
nodes
edges
GO
1
2
3
4
5
6
7
8
9
10
11
12
13
14
15
16
17
18
19
20
21
22
23
24
25
26
27
28
29
30
31
32
33
34
35
36
37
38
39
40
41
42
43
44
45
46
47
48
49
50
51

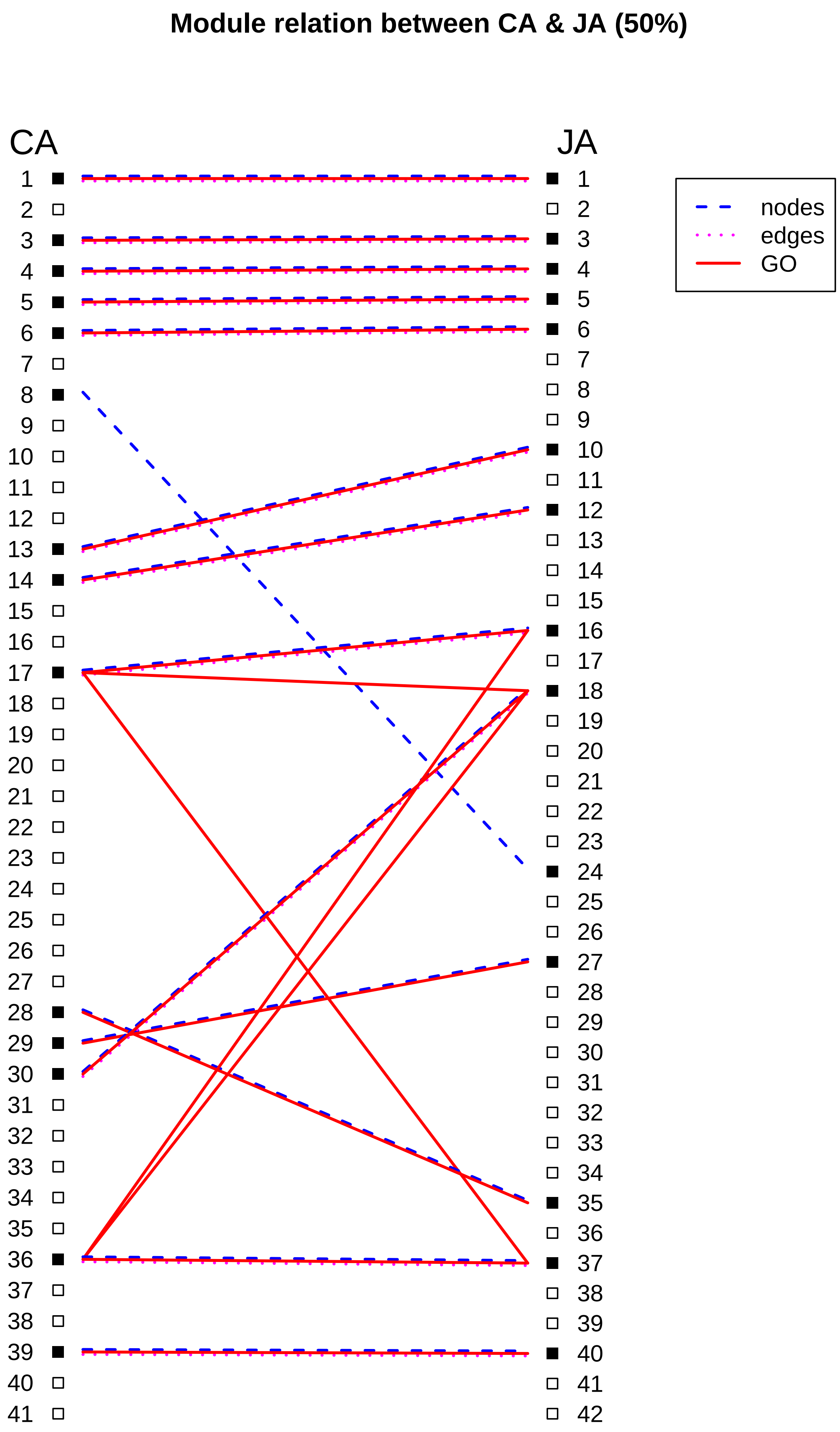

Module relation between CA & JA (50%)
CA
JA
nodes
edges
GO

Module relation between CA & Vehicle (50%)

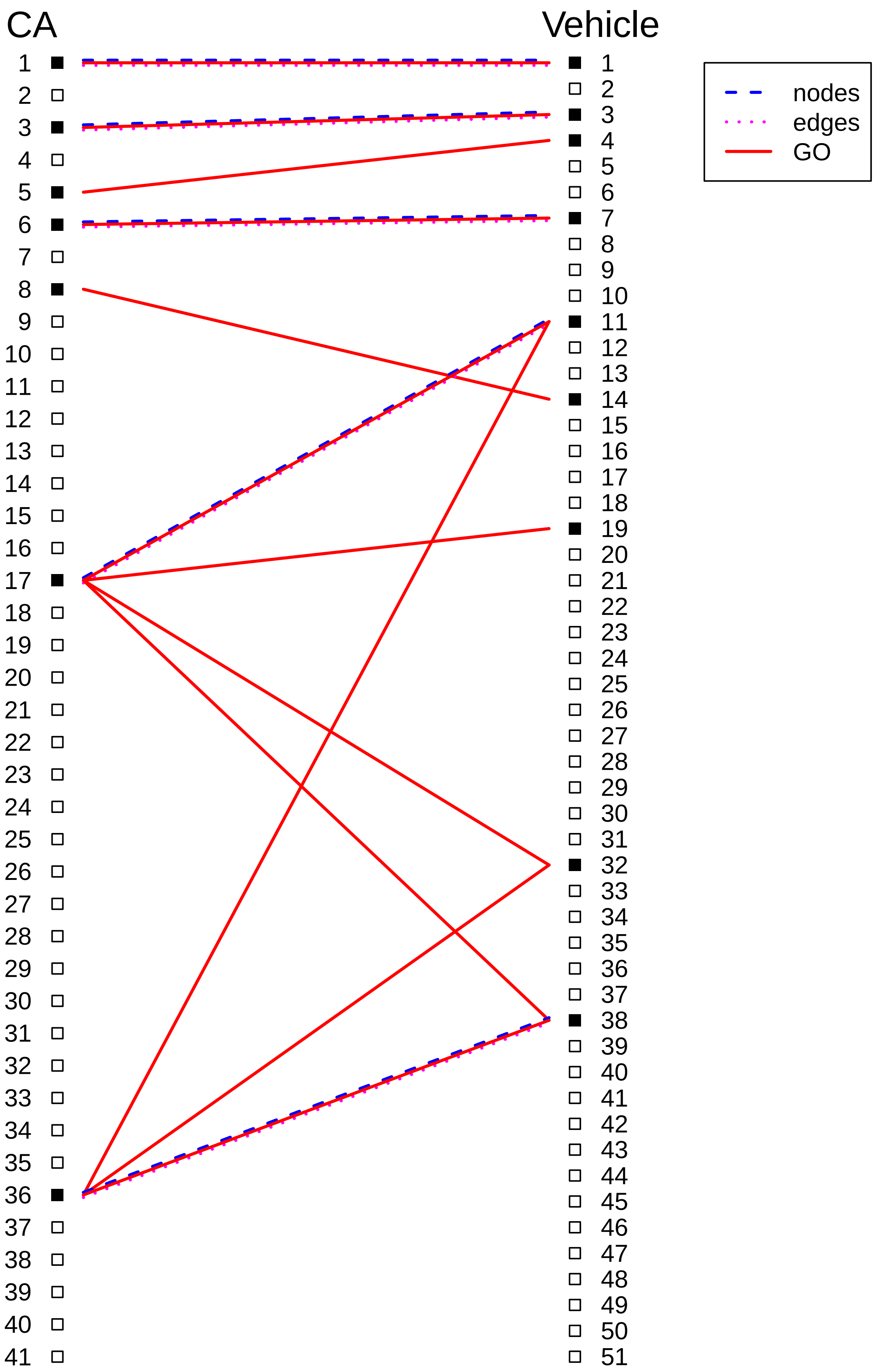

Module relation between JA & Vehicle (50%)

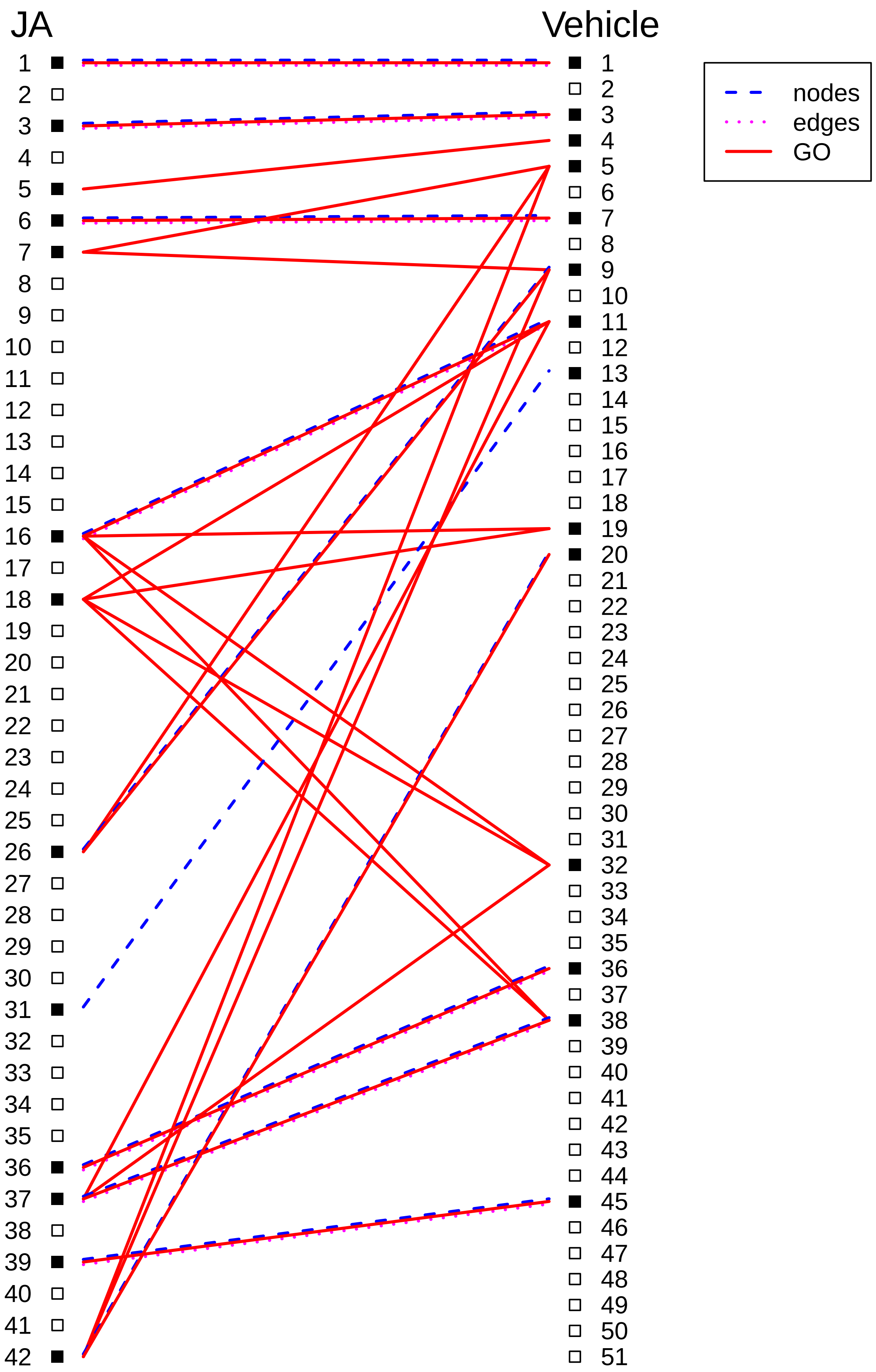

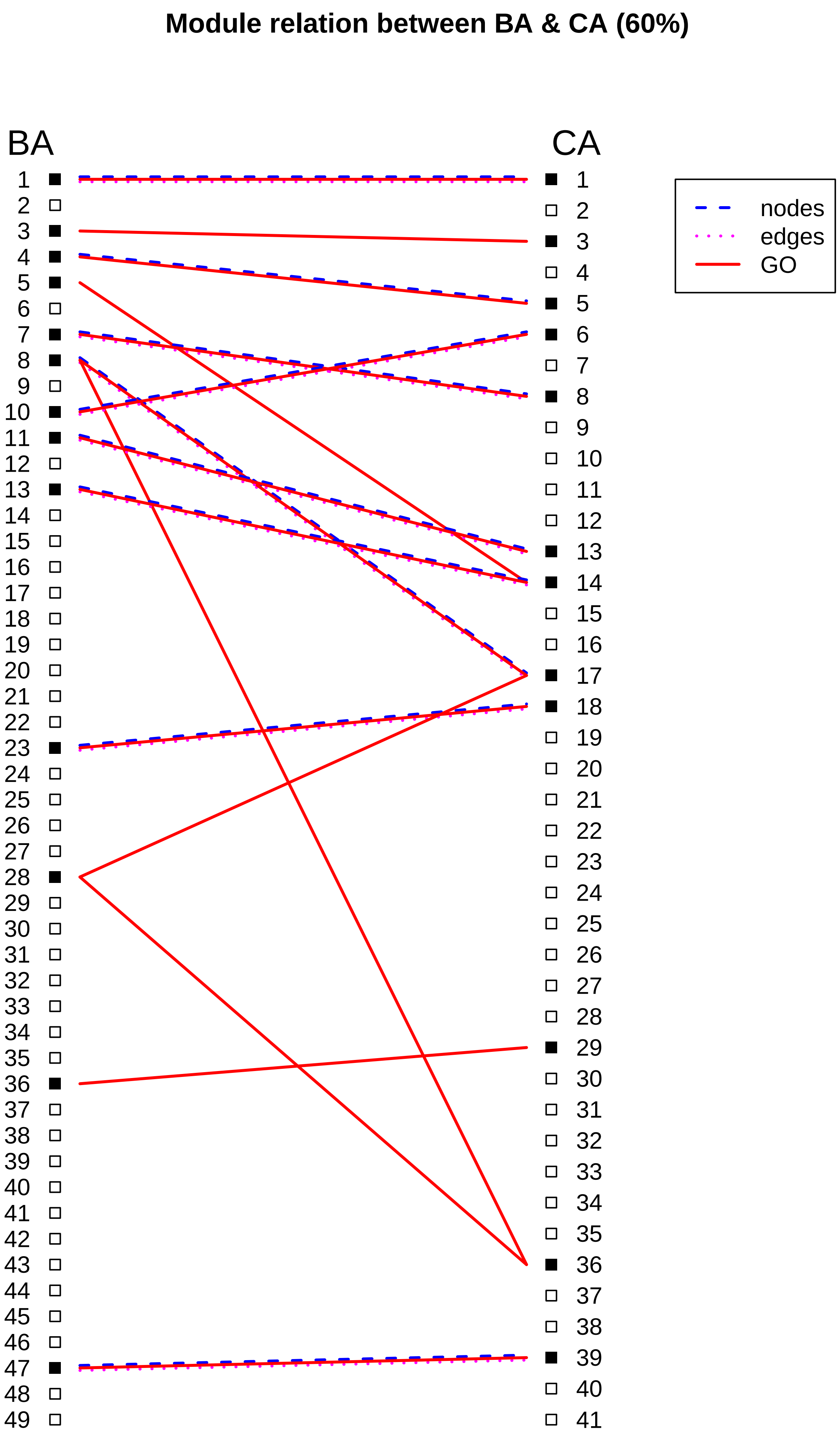
Module relation between BA & CA (60%)
BA
CA
nodes
edges
GO
1
2
3
4
5
6
7
8
9
10
11
12
13
14
15
16
17
18
19
20
21
22
23
24
25
26
27
28
29
30
31
32
33
34
35
36
37
38
39
40
41
42
43
44
45
46
47
48
49

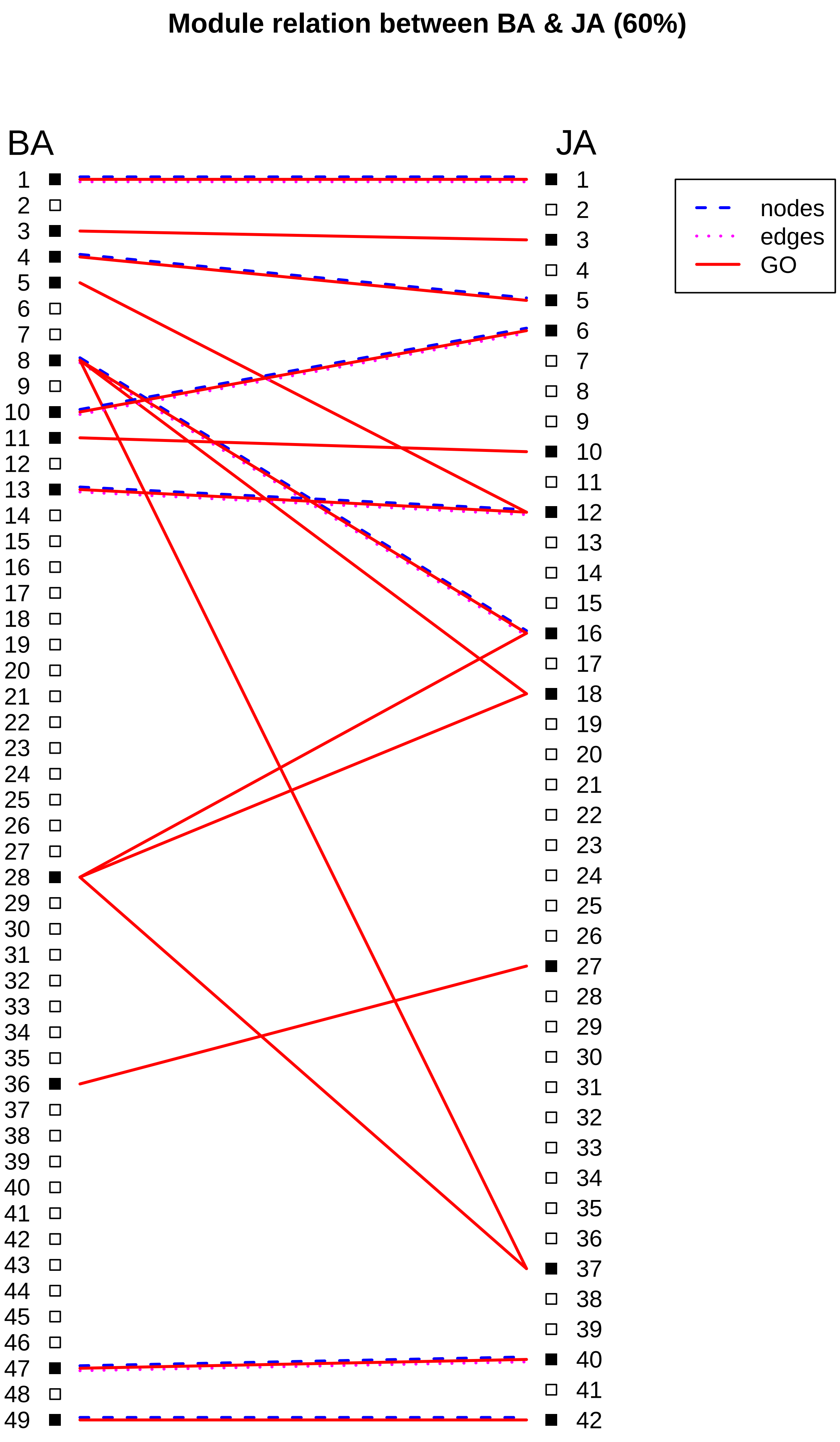
Module relation between BA & JA (60%)
BA
JA
nodes
edges
GO
1
2
3
4
5
6
7
8
9
10
11
12
13
14
15
16
17
18
19
20
21
22
23
24
25
26
27
28
29
30
31
32
33
34
35
36
37
38
39
40
41
42
43
44
45
46
47
48
49

Module relation between BA & Vehicle (60%)

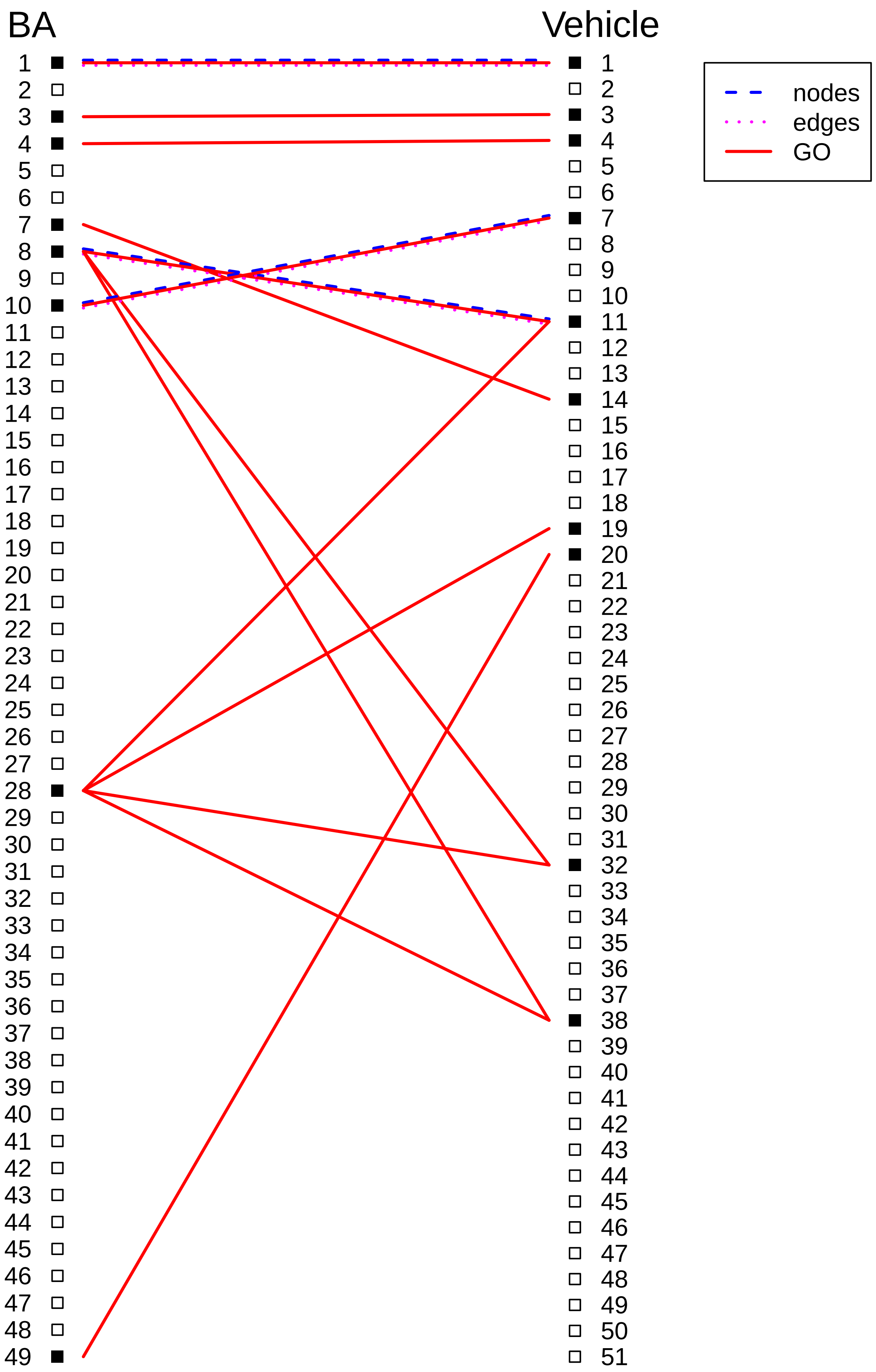

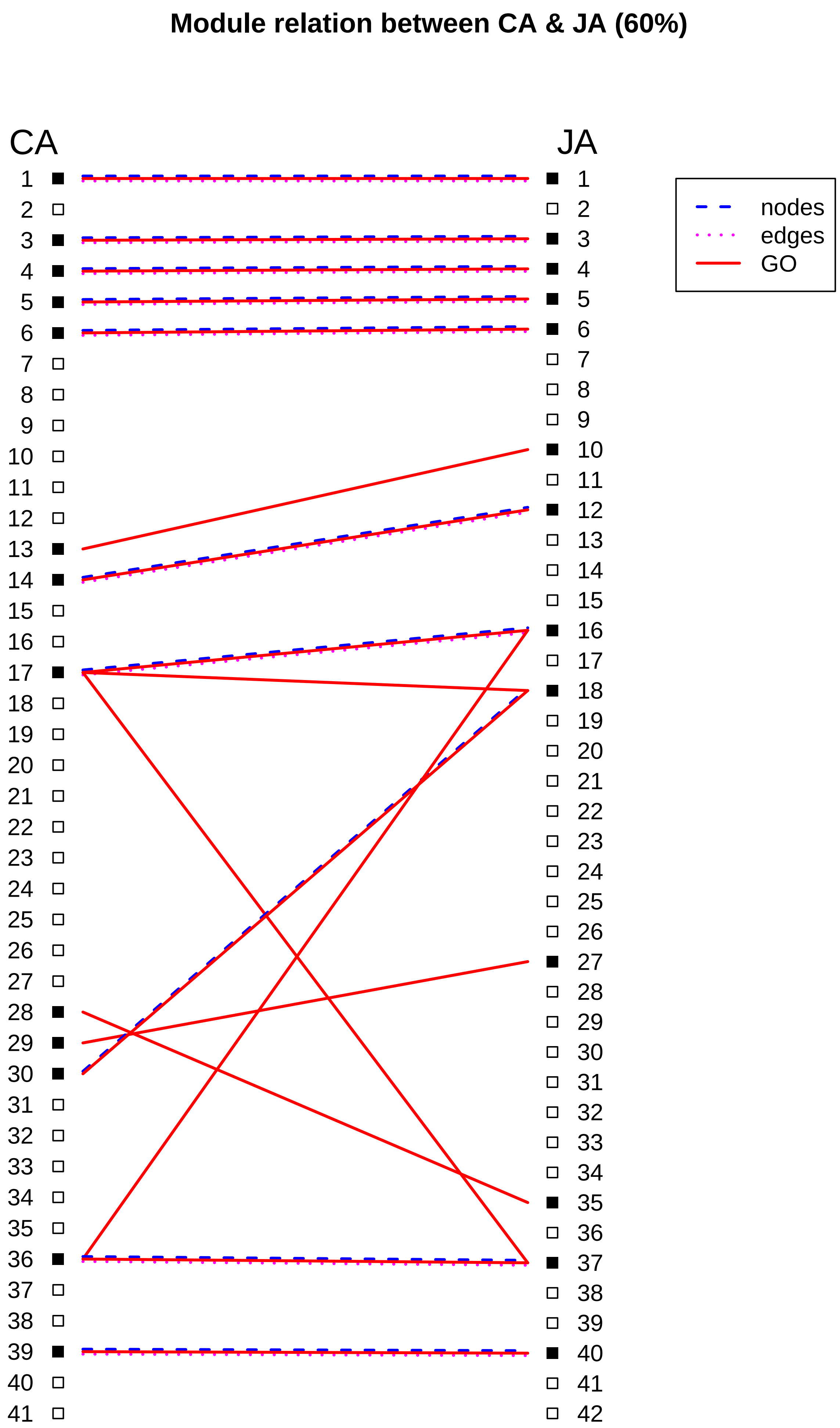
Module relation between CA & JA (60%)
CA
JA
1 2 3 4 5 6 7 8 9 10 11 12 13 14 15 16 17 18 19 20 21 22 23 24 25 26 27 28 29 30 31 32 33 34 35 36 37 38 39 40 41
1 2 3 4 5 6 7 8 9 10 11 12 13 14 15 16 17 18 19 20 21 22 23 24 25 26 27 28 29 30 31 32 33 34 35 36 37 38 39 40 41 42
nodes
edges
GO

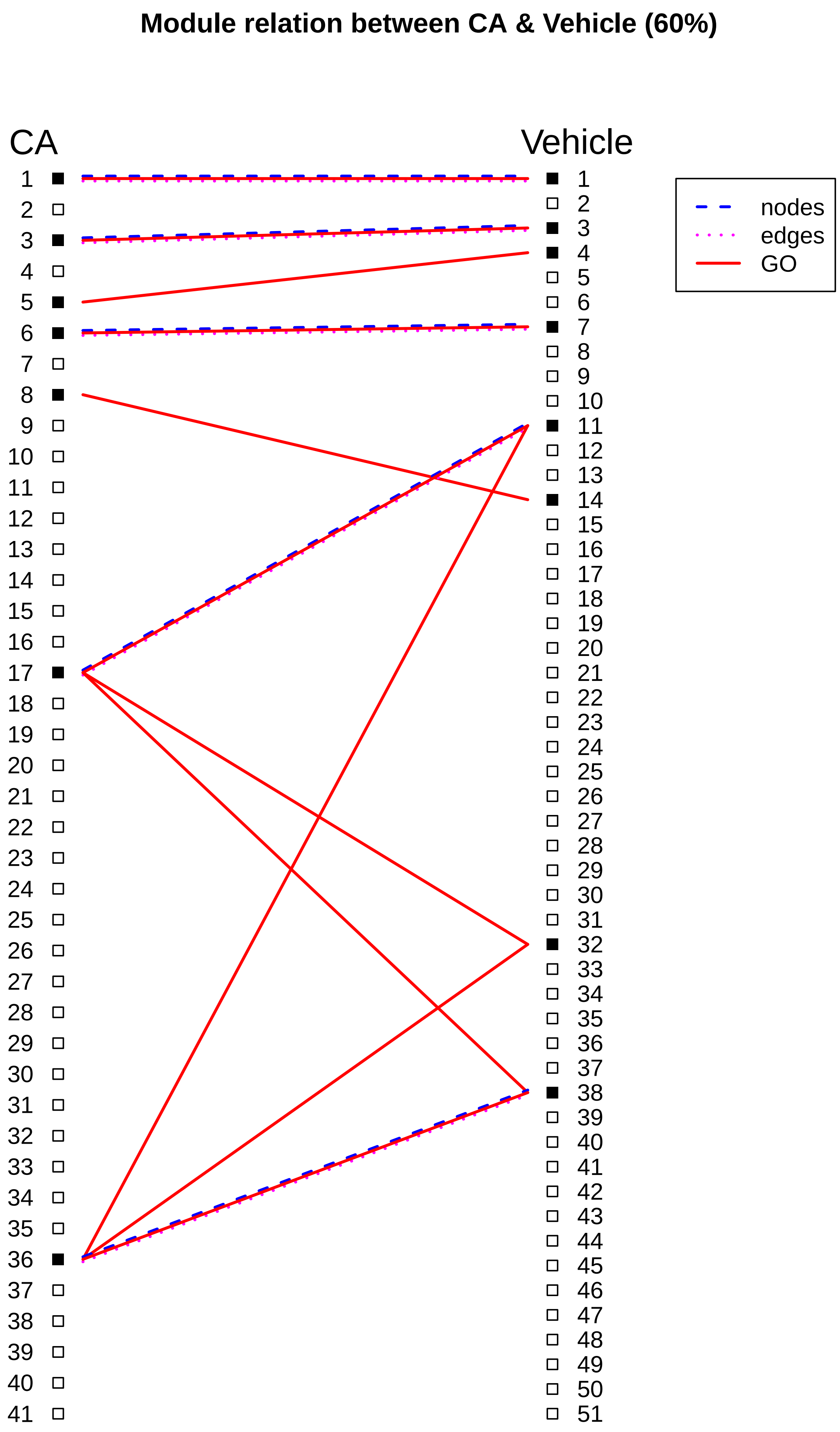

Module relation between CA & Vehicle (60%)
CA
Vehicle
nodes
edges
GO
1
2
3
4
5
6
7
8
9
10
11
12
13
14
15
16
17
18
19
20
21
22
23
24
25
26
27
28
29
30
31
32
33
34
35
36
37
38
39
40
41
42
43
44
45
46
47
48
49
50
51

Module relation between JA & Vehicle (60%)

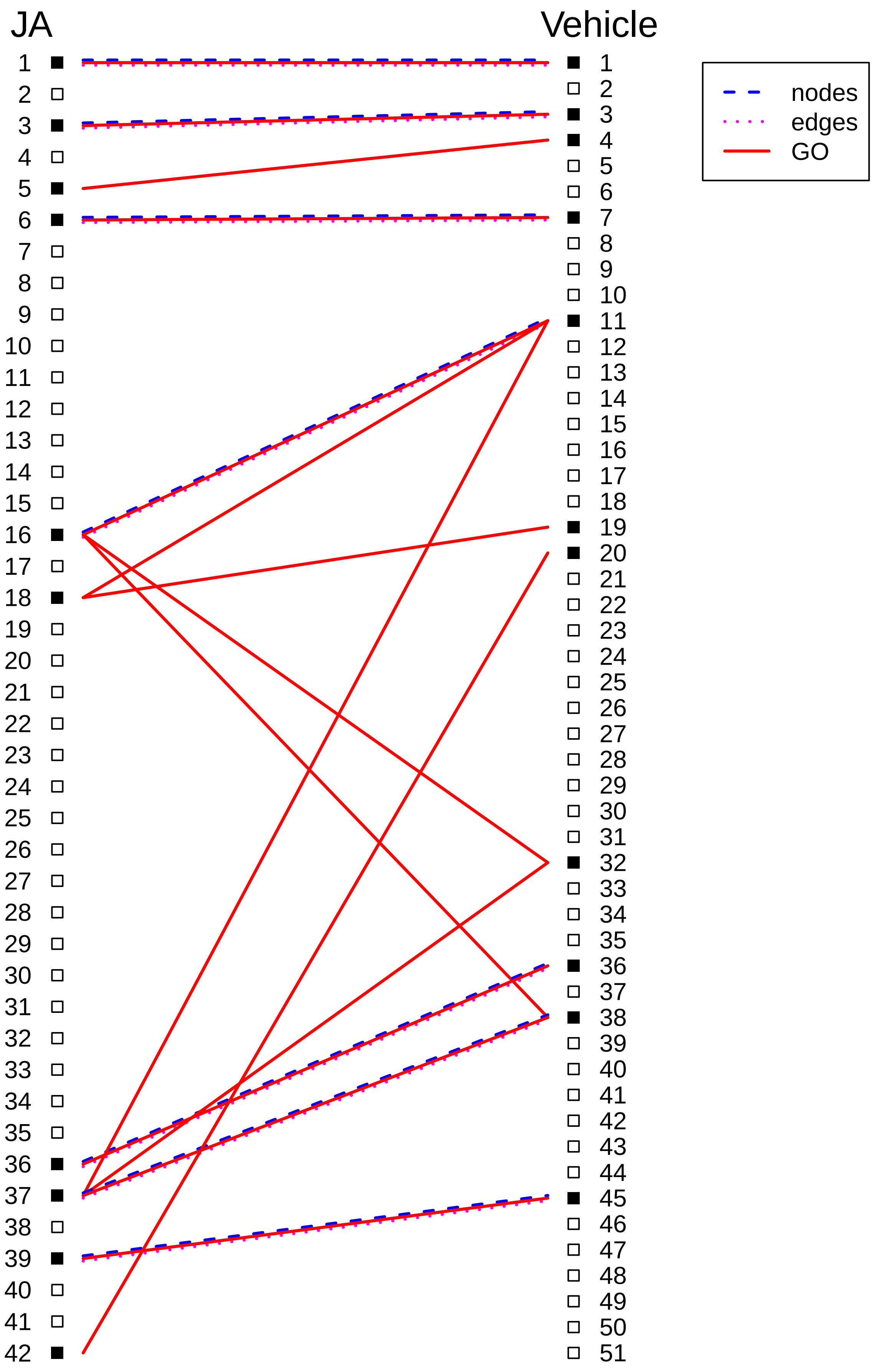

Module relation between BA & CA (75%)

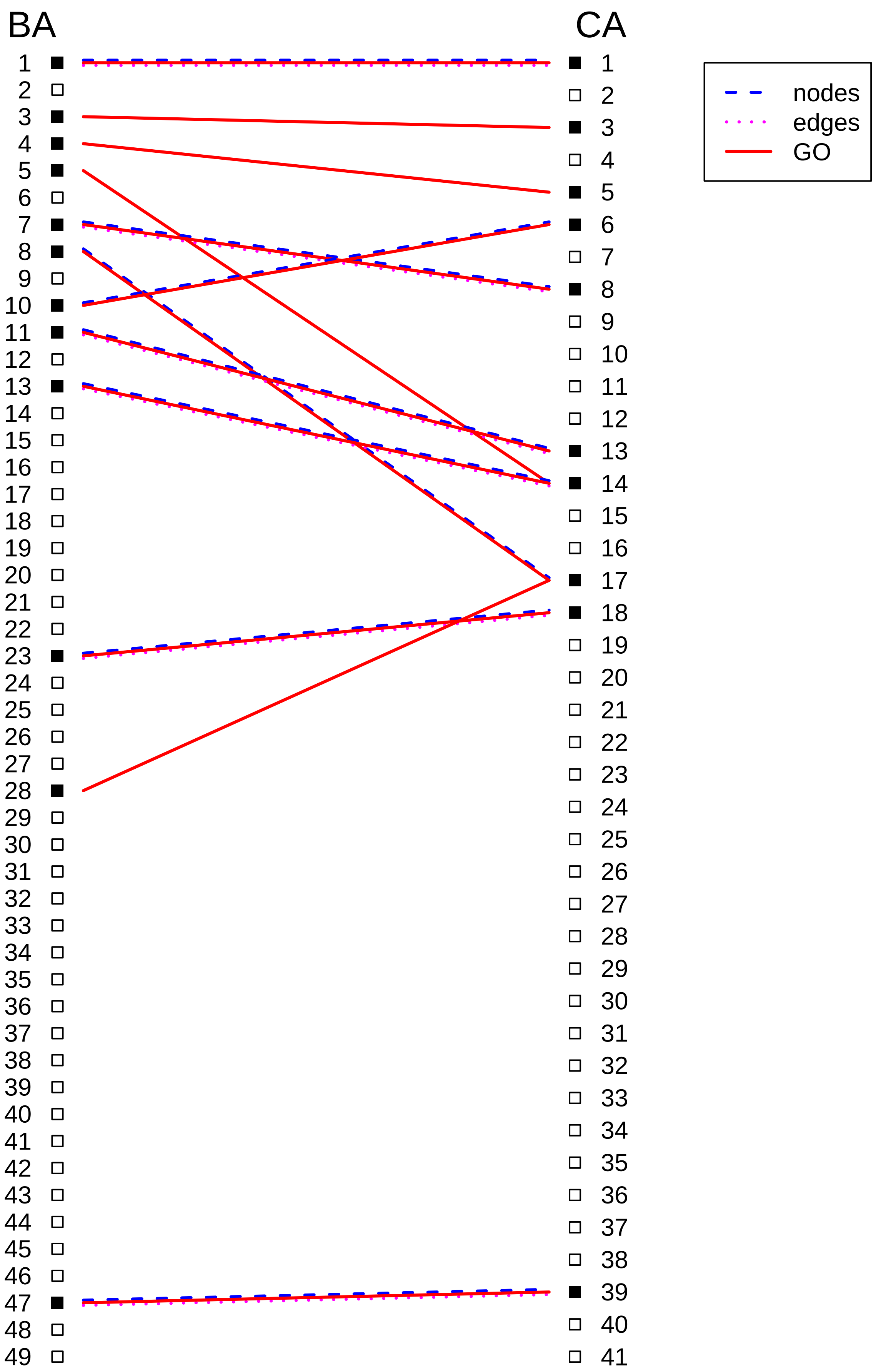

Module relation between BA & JA (75%)

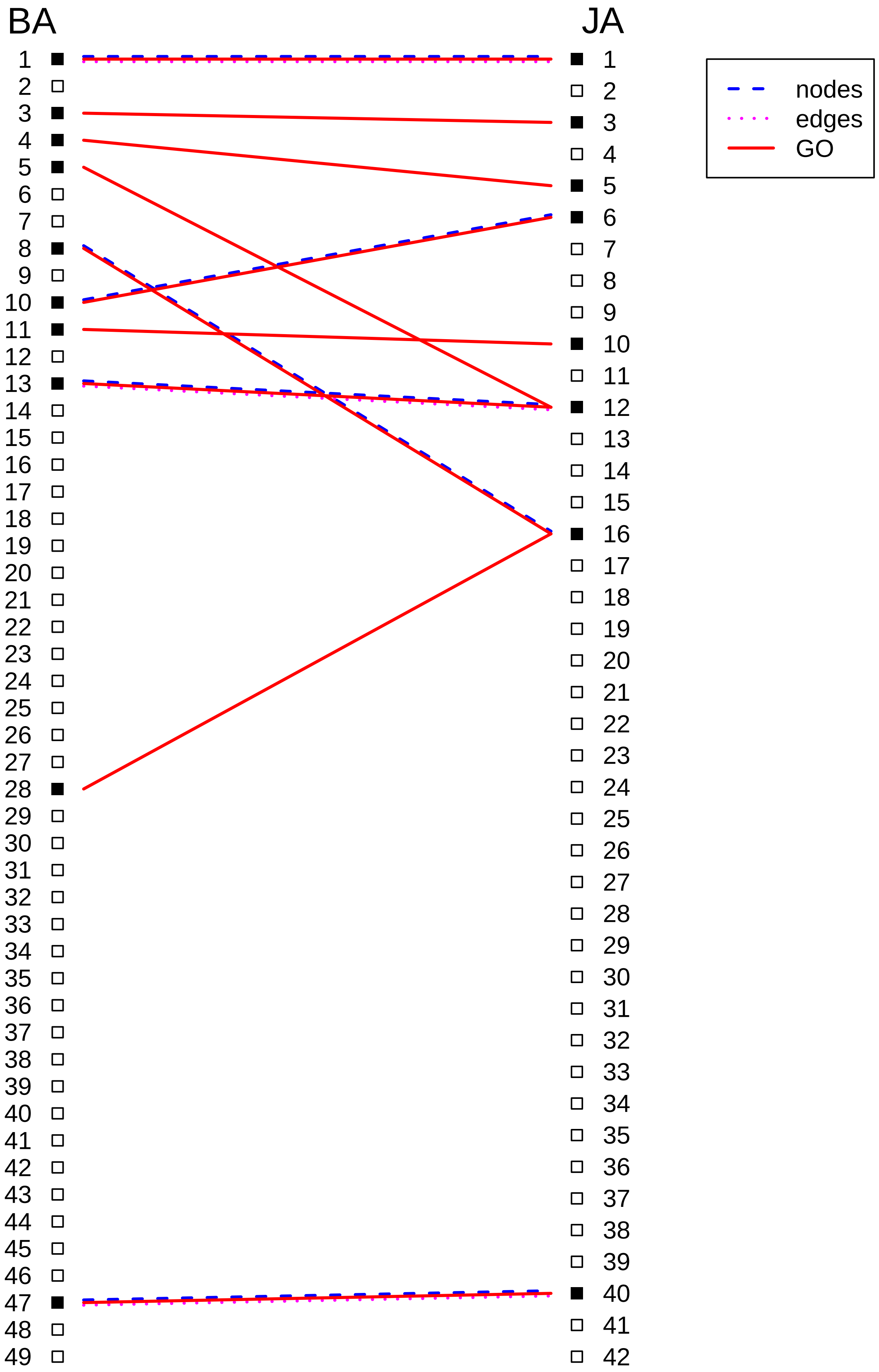

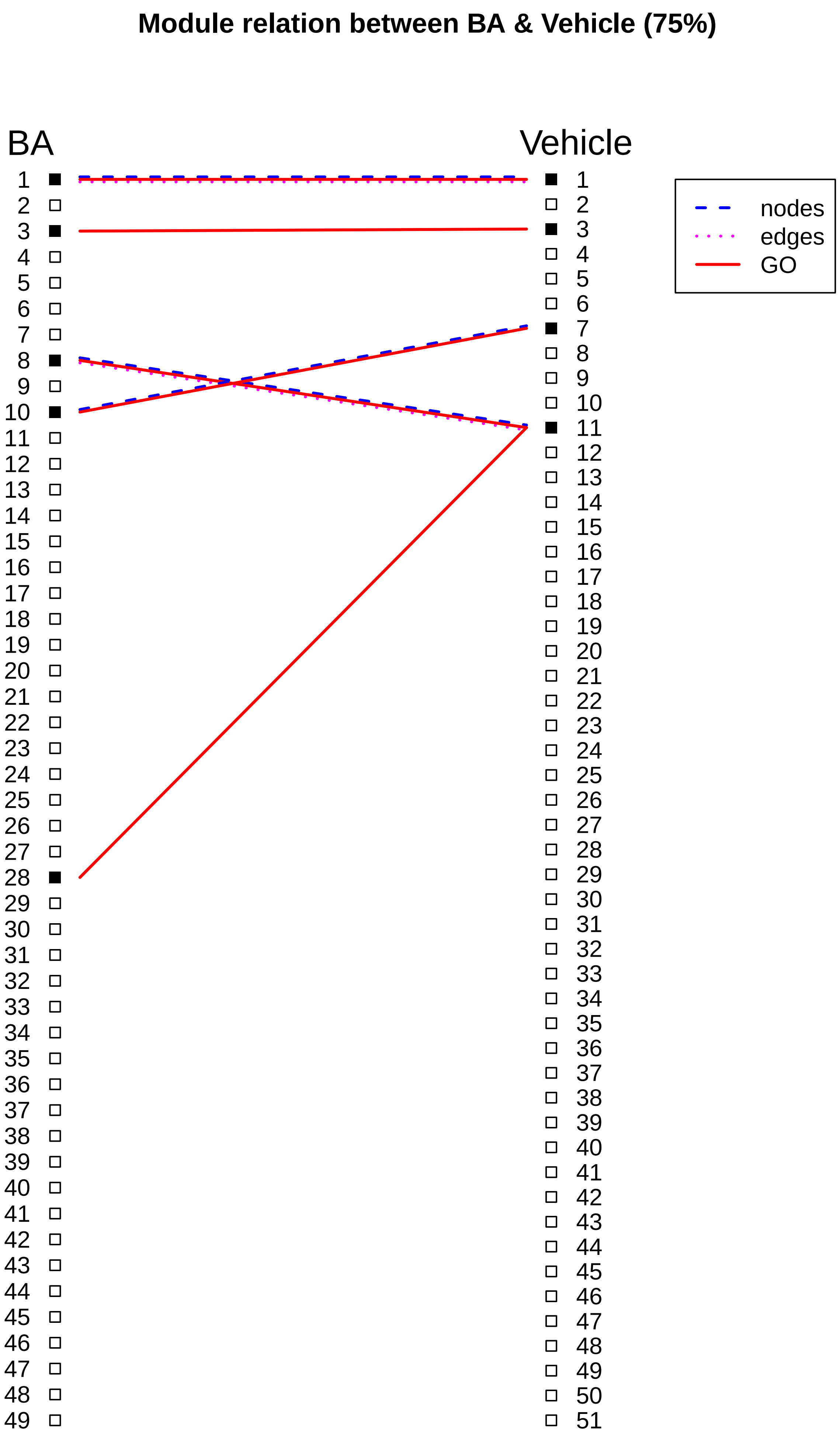
Module relation between BA & Vehicle (75%)
BA
Vehicle
nodes
edges
GO
1
2
3
4
5
6
7
8
9
10
11
12
13
14
15
16
17
18
19
20
21
22
23
24
25
26
27
28
29
30
31
32
33
34
35
36
37
38
39
40
41
42
43
44
45
46
47
48
49
50
51

Module relation between CA & JA (75%)

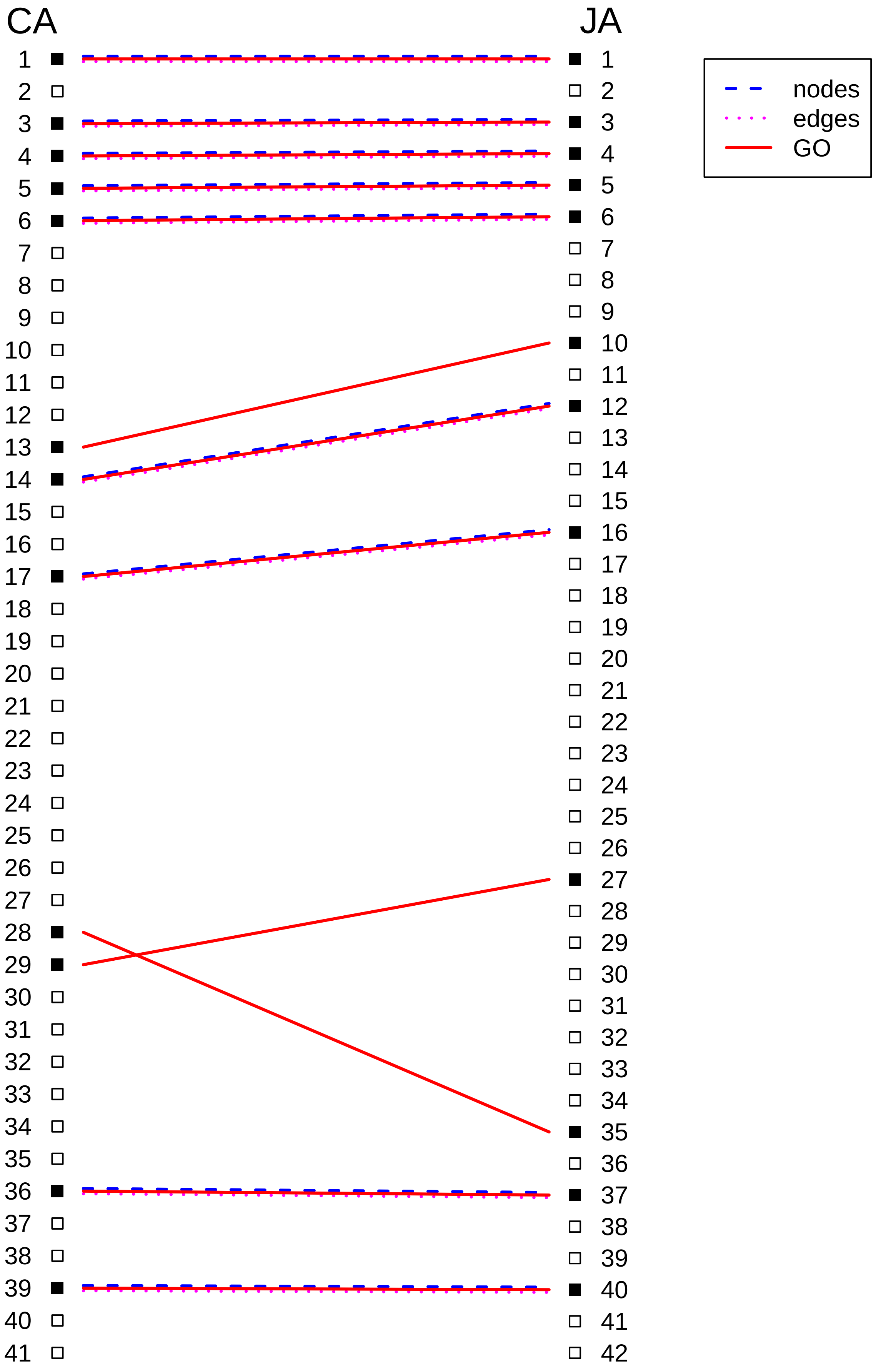

Module relation between CA & Vehicle (75%)

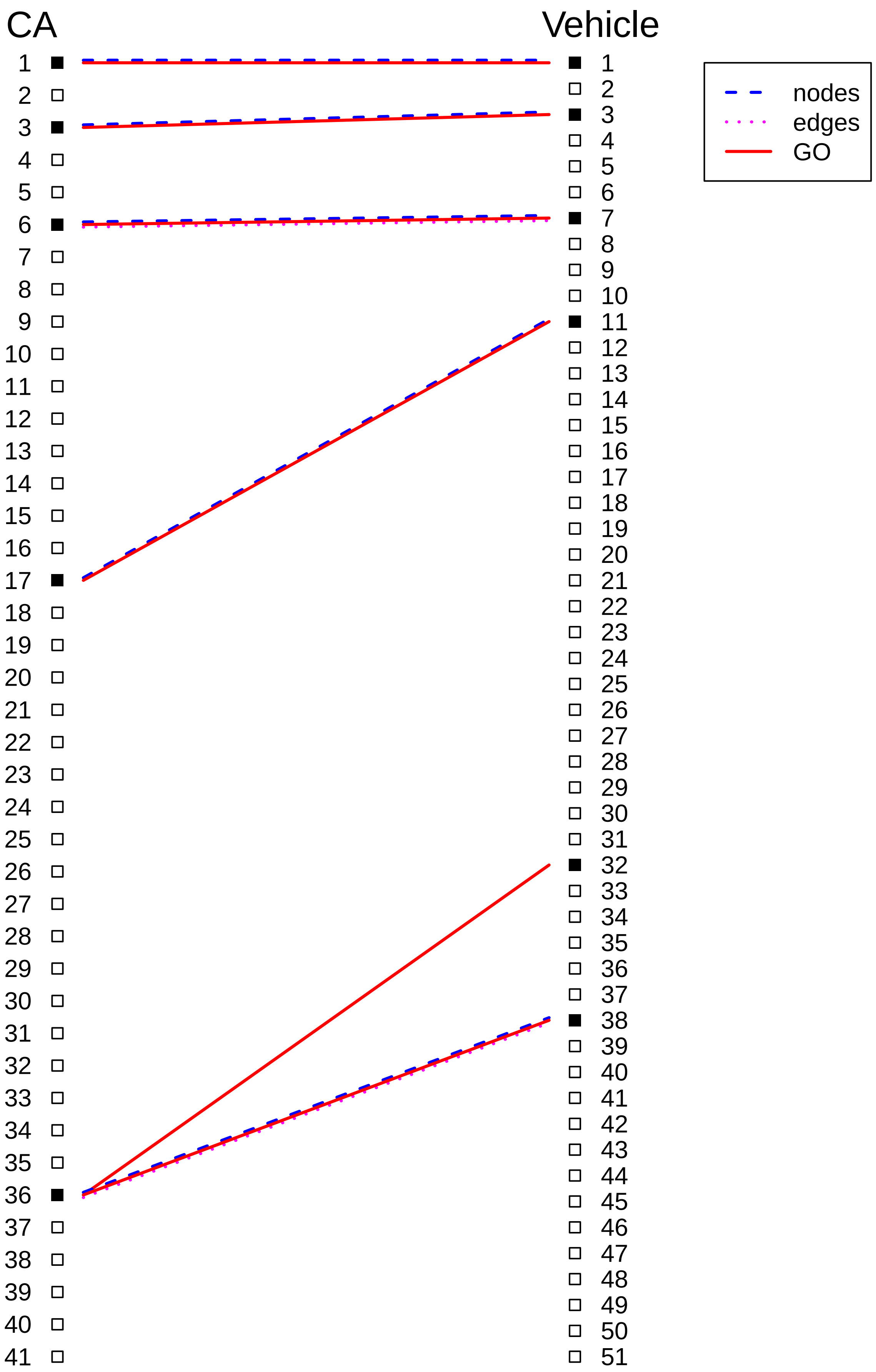

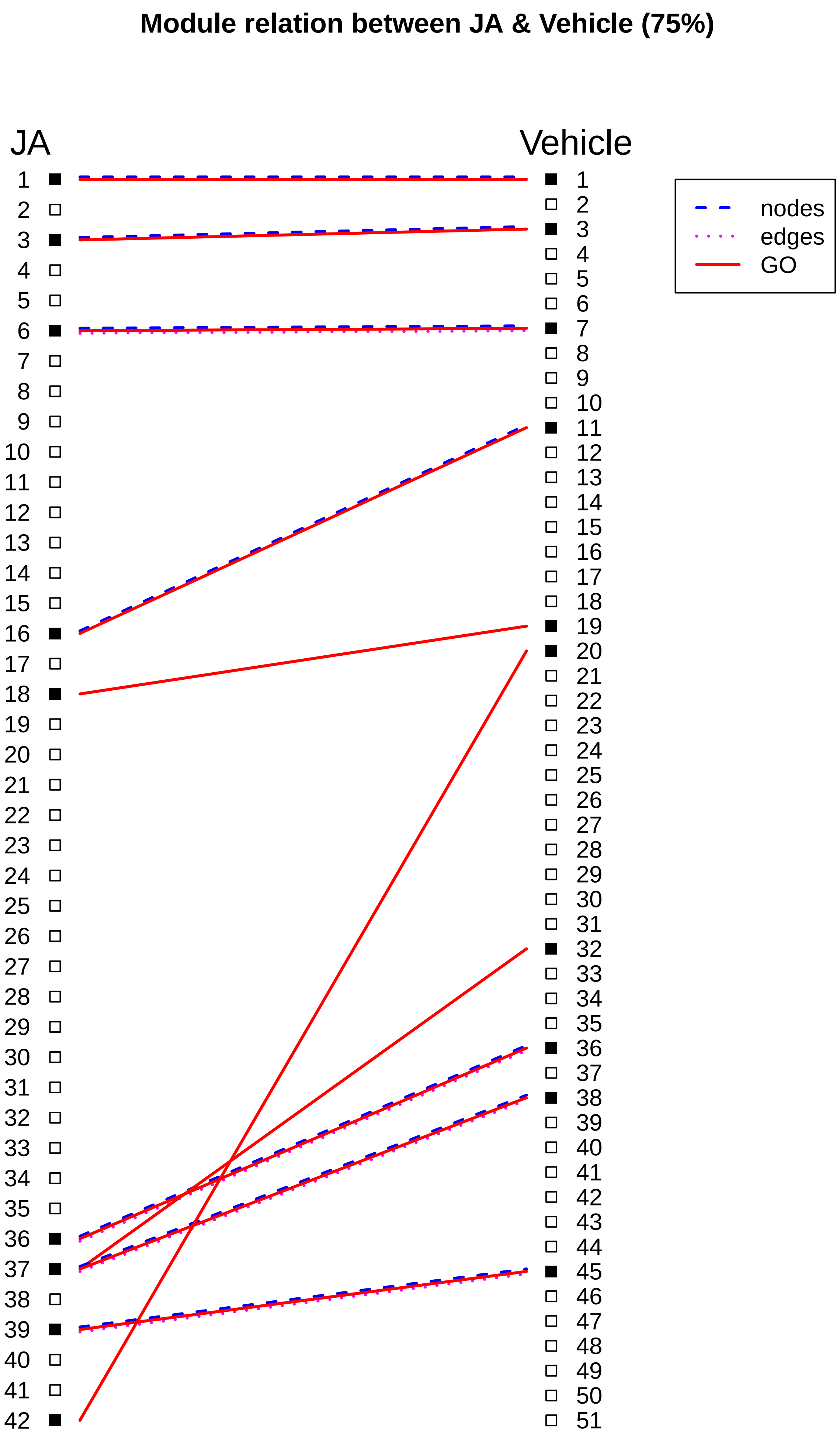
Module relation between JA & Vehicle (75%)
JA
Vehicle
nodes
edges
GO
1
2
3
4
5
6
7
8
9
10
11
12
13
14
15
16
17
18
19
20
21
22
23
24
25
26
27
28
29
30
31
32
33
34
35
36
37
38
39
40
41
42
43
44
45
46
47
48
49
50
51

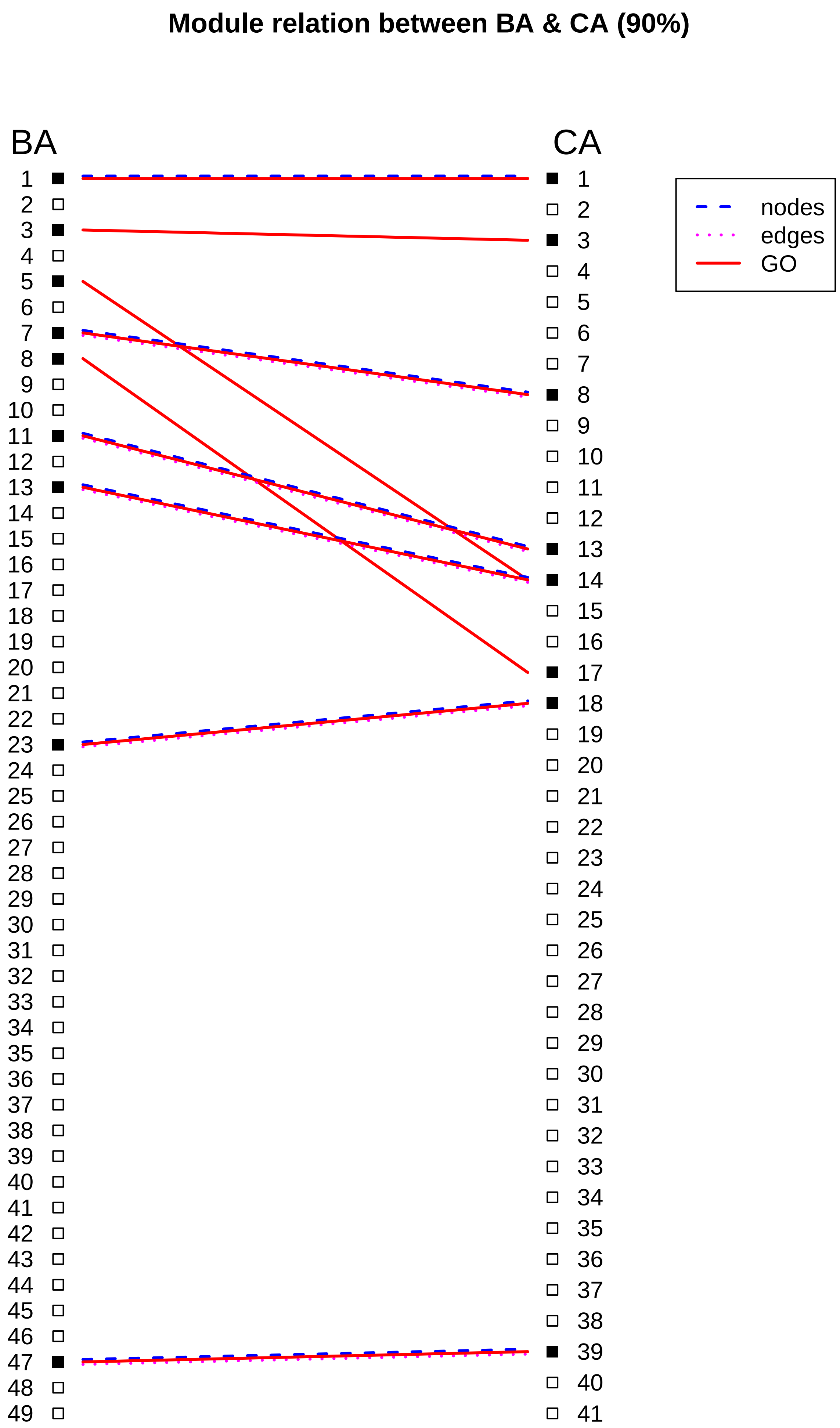
Module relation between BA & CA (90%)
BA
CA
1
2
3
4
5
6
7
8
9
10
11
12
13
14
15
16
17
18
19
20
21
22
23
24
25
26
27
28
29
30
31
32
33
34
35
36
37
38
39
40
41
42
43
44
45
46
47
48
49
nodes
edges
GO

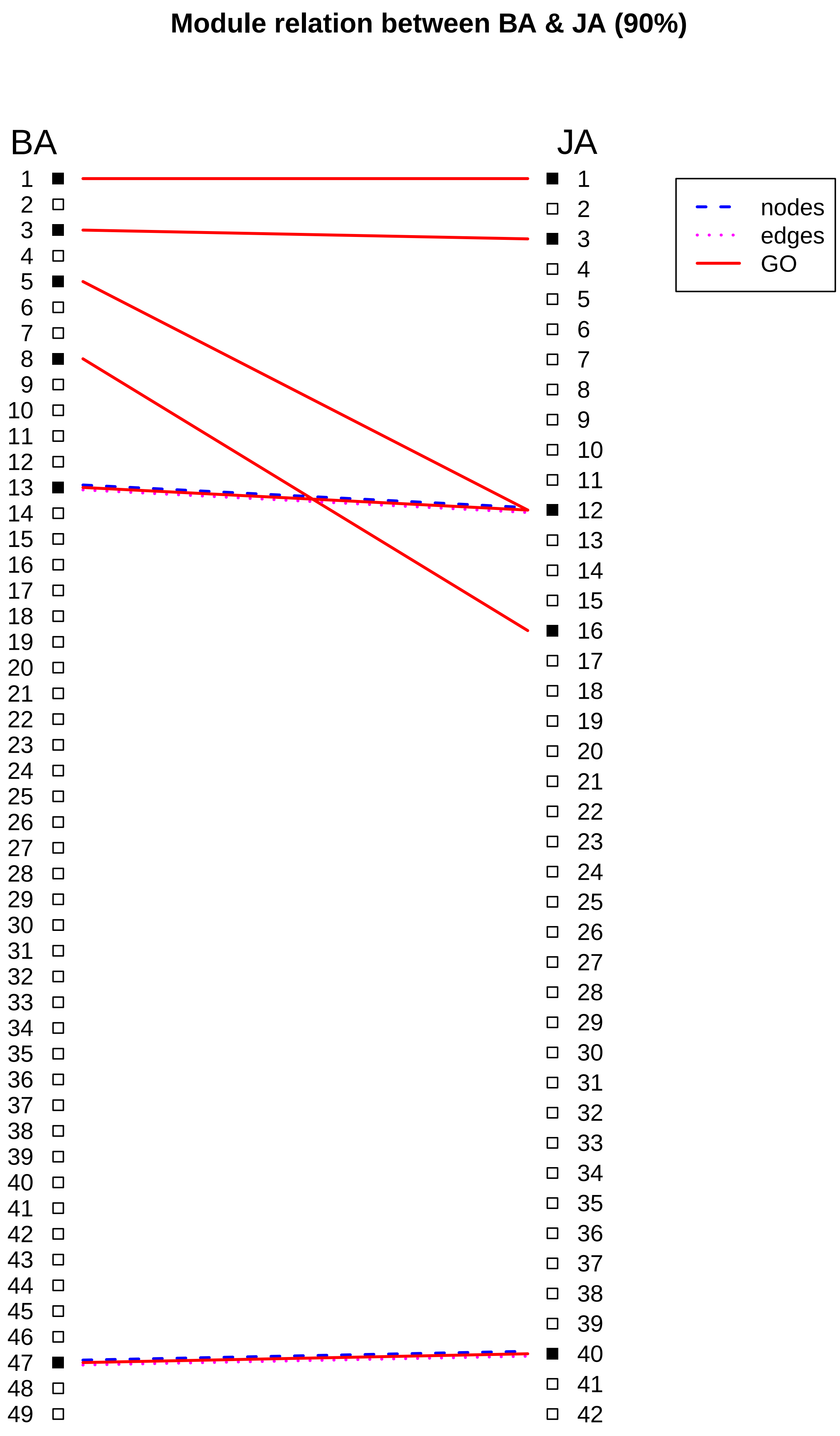
Module relation between BA & JA (90%)
BA
JA
nodes
edges
GO
1
2
3
4
5
6
7
8
9
10
11
12
13
14
15
16
17
18
19
20
21
22
23
24
25
26
27
28
29
30
31
32
33
34
35
36
37
38
39
40
41
42
43
44
45
46
47
48
49

Module relation between BA & Vehicle (90%)

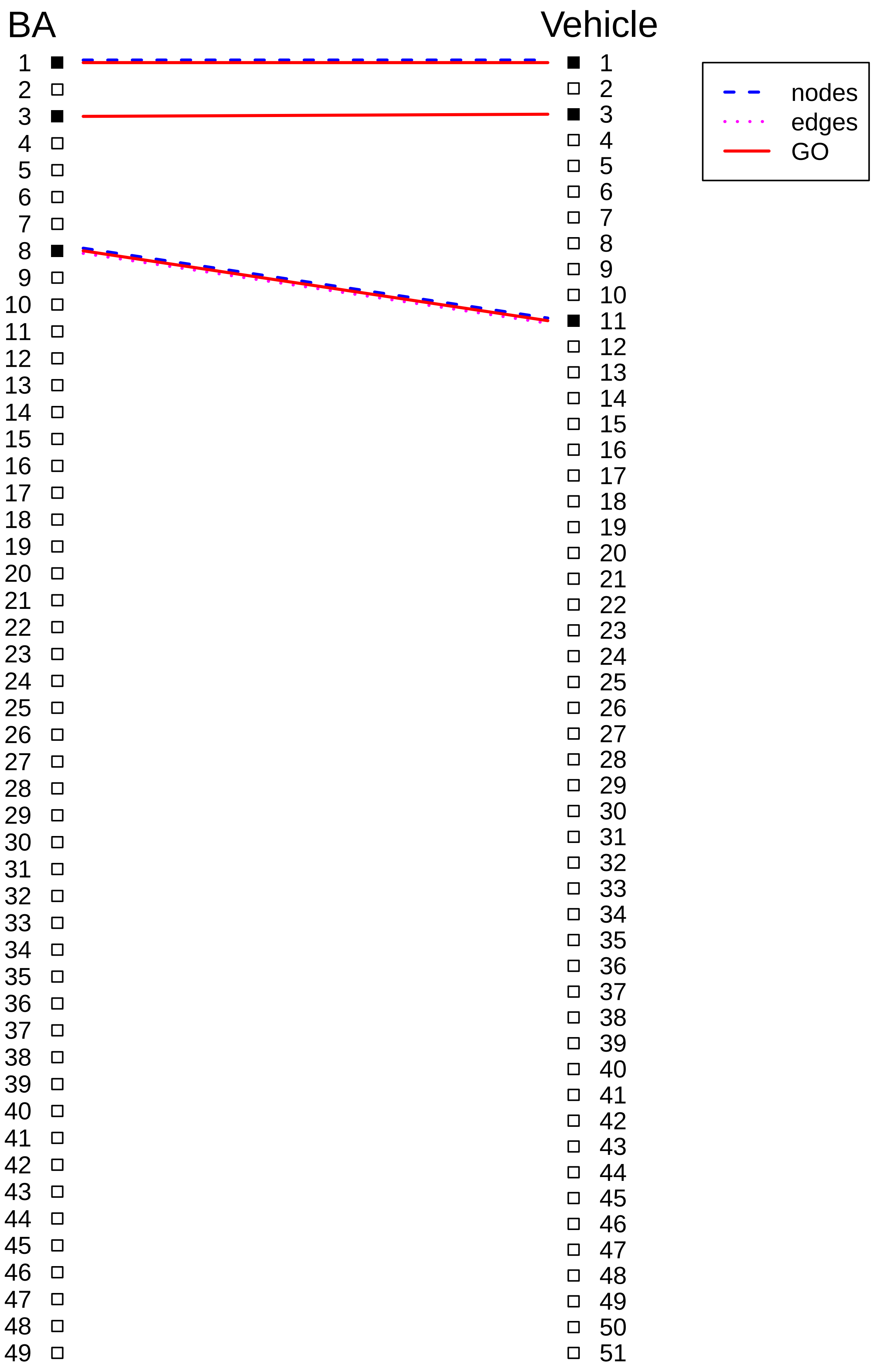

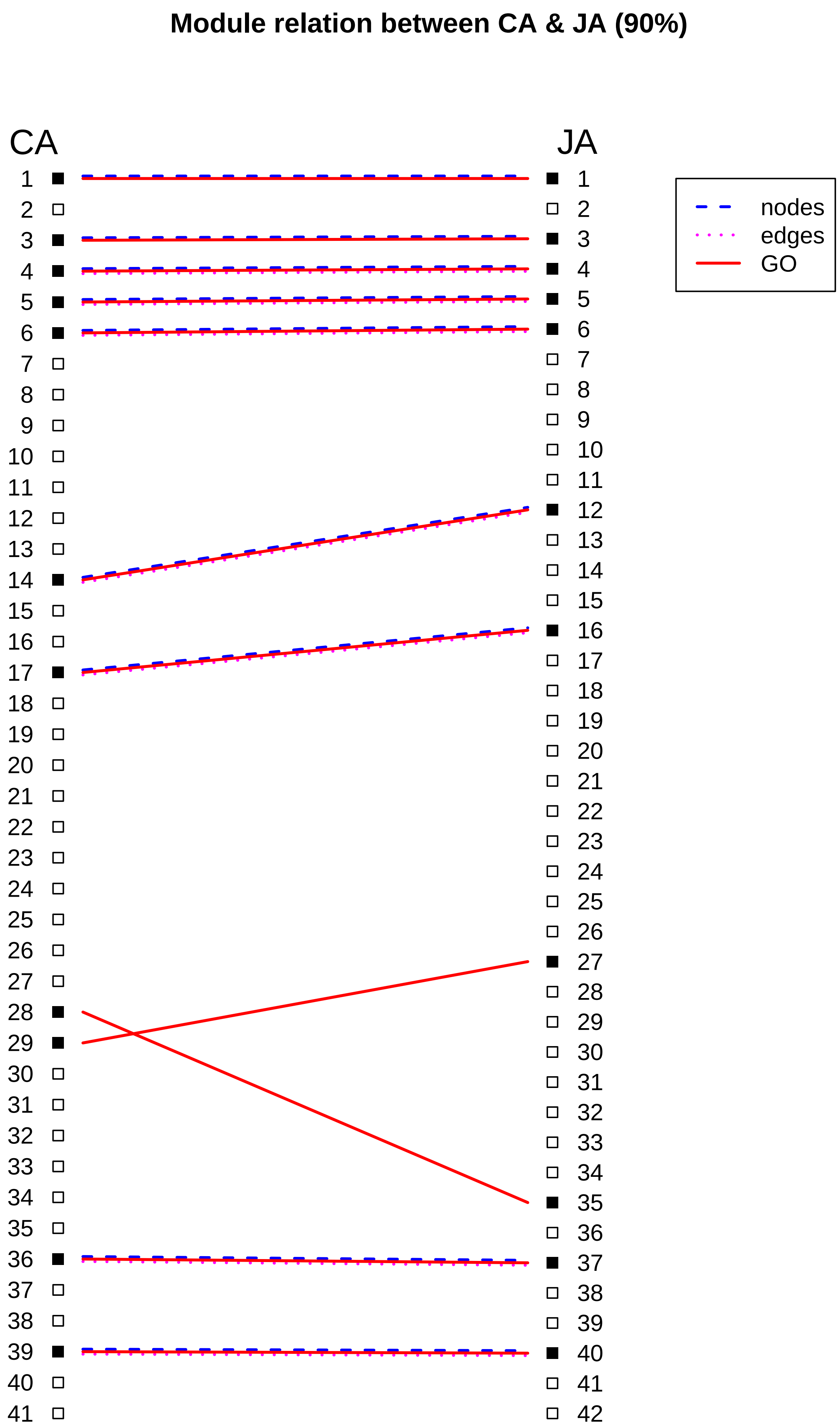
Module relation between CA & JA (90%)
CA
JA
1
2
3
4
5
6
7
8
9
10
11
12
13
14
15
16
17
18
19
20
21
22
23
24
25
26
27
28
29
30
31
32
33
34
35
36
37
38
39
40
41
42
nodes
edges
GO

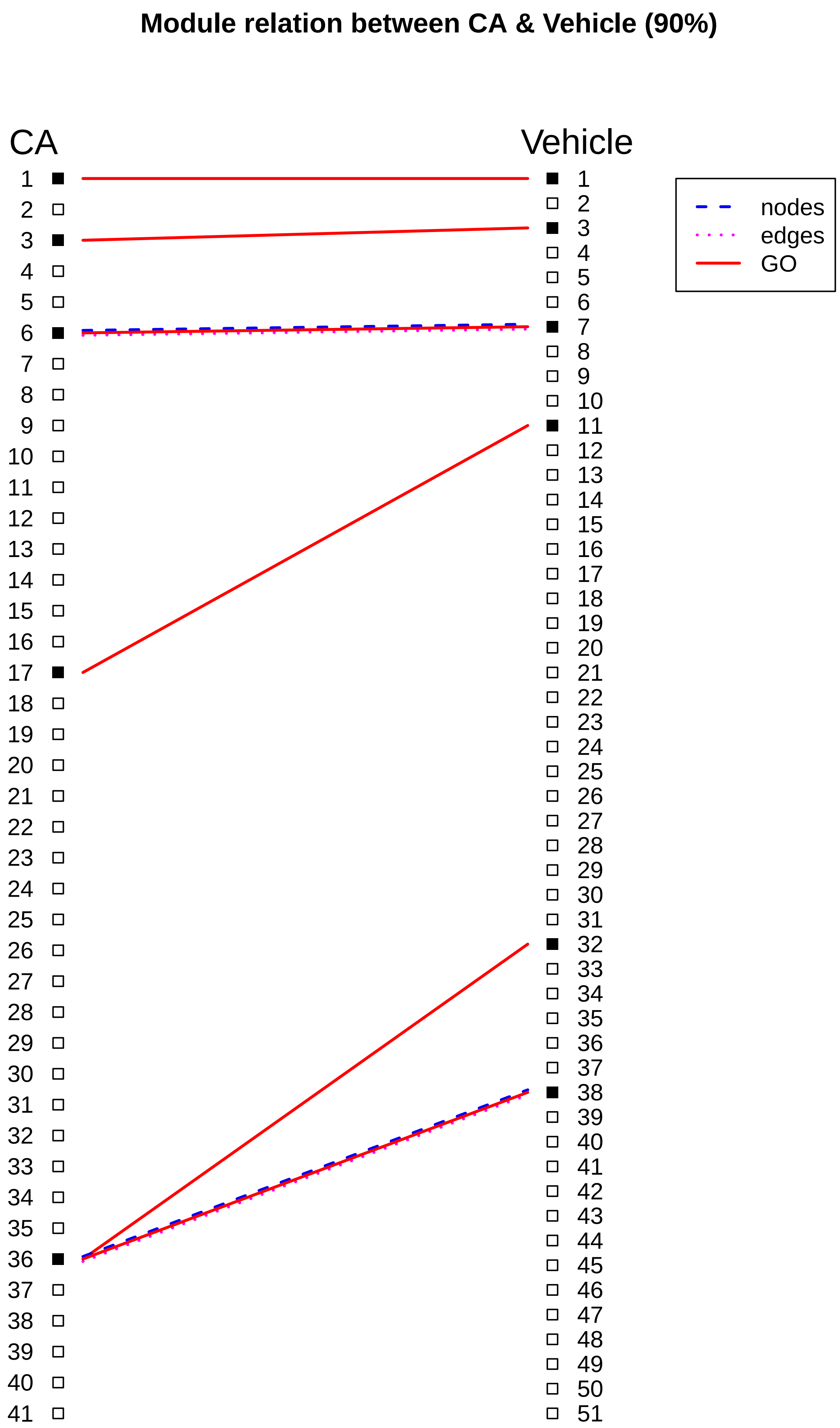
Module relation between CA & Vehicle (90%)
CA
Vehicle
nodes
edges
GO
1 2 3 4 5 6 7 8 9 10 11 12 13 14 15 16 17 18 19 20 21 22 23 24 25 26 27 28 29 30 31 32 33 34 35 36 37 38 39 40 41
1 2 3 4 5 6 7 8 9 10 11 12 13 14 15 16 17 18 19 20 21 22 23 24 25 26 27 28 29 30 31 32 33 34 35 36 37 38 39 40 41 42 43 44 45 46 47 48 49 50 51

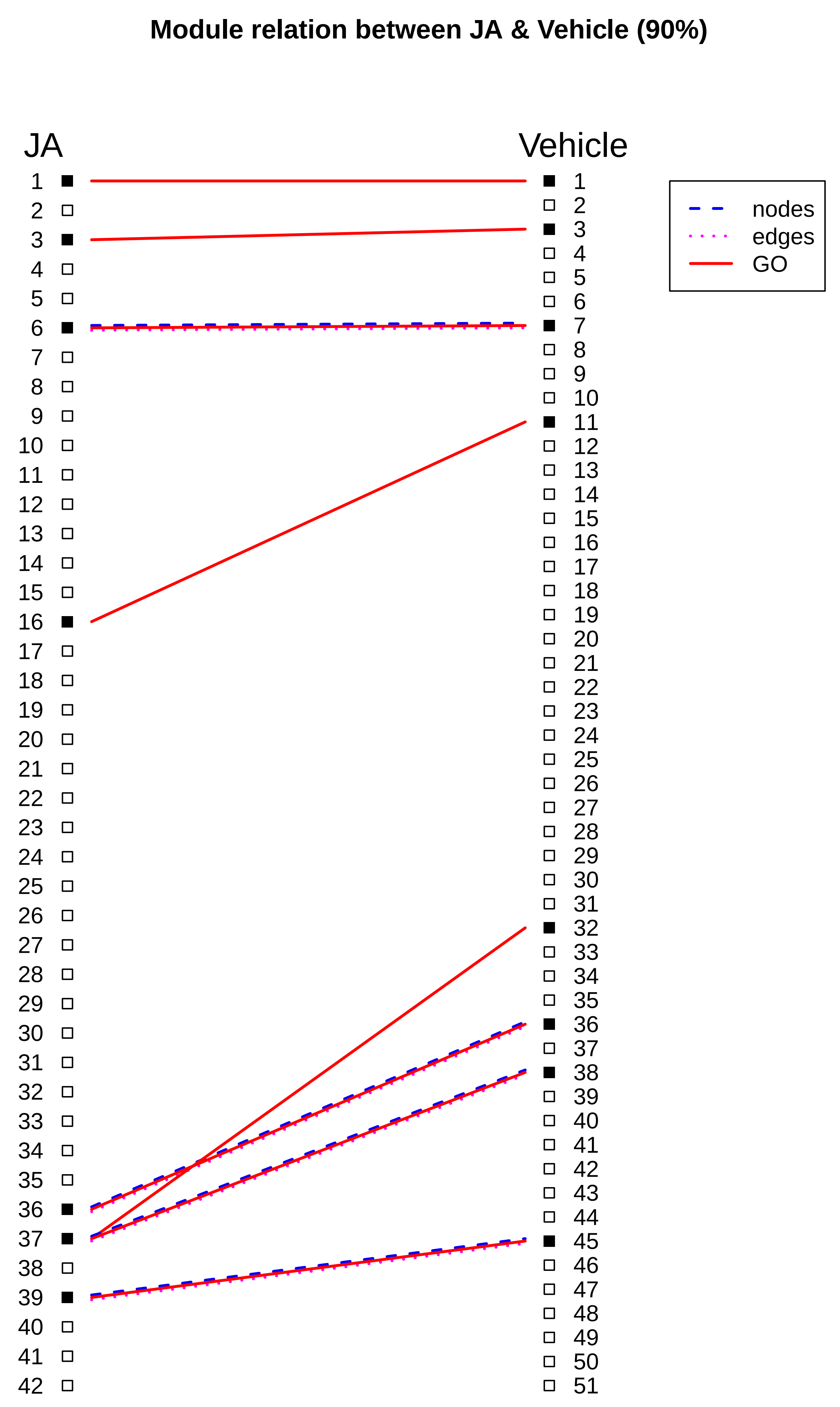
Module relation between JA & Vehicle (90%)
JA
Vehicle
nodes
edges
GO
1 2 3 4 5 6 7 8 9 10 11 12 13 14 15 16 17 18 19 20 21 22 23 24 25 26 27 28 29 30 31 32 33 34 35 36 37 38 39 40 41 42
1 2 3 4 5 6 7 8 9 10 11 12 13 14 15 16 17 18 19 20 21 22 23 24 25 26 27 28 29 30 31 32 33 34 35 36 37 38 39 40 41 42 43 44 45 46 47 48 49 50 51

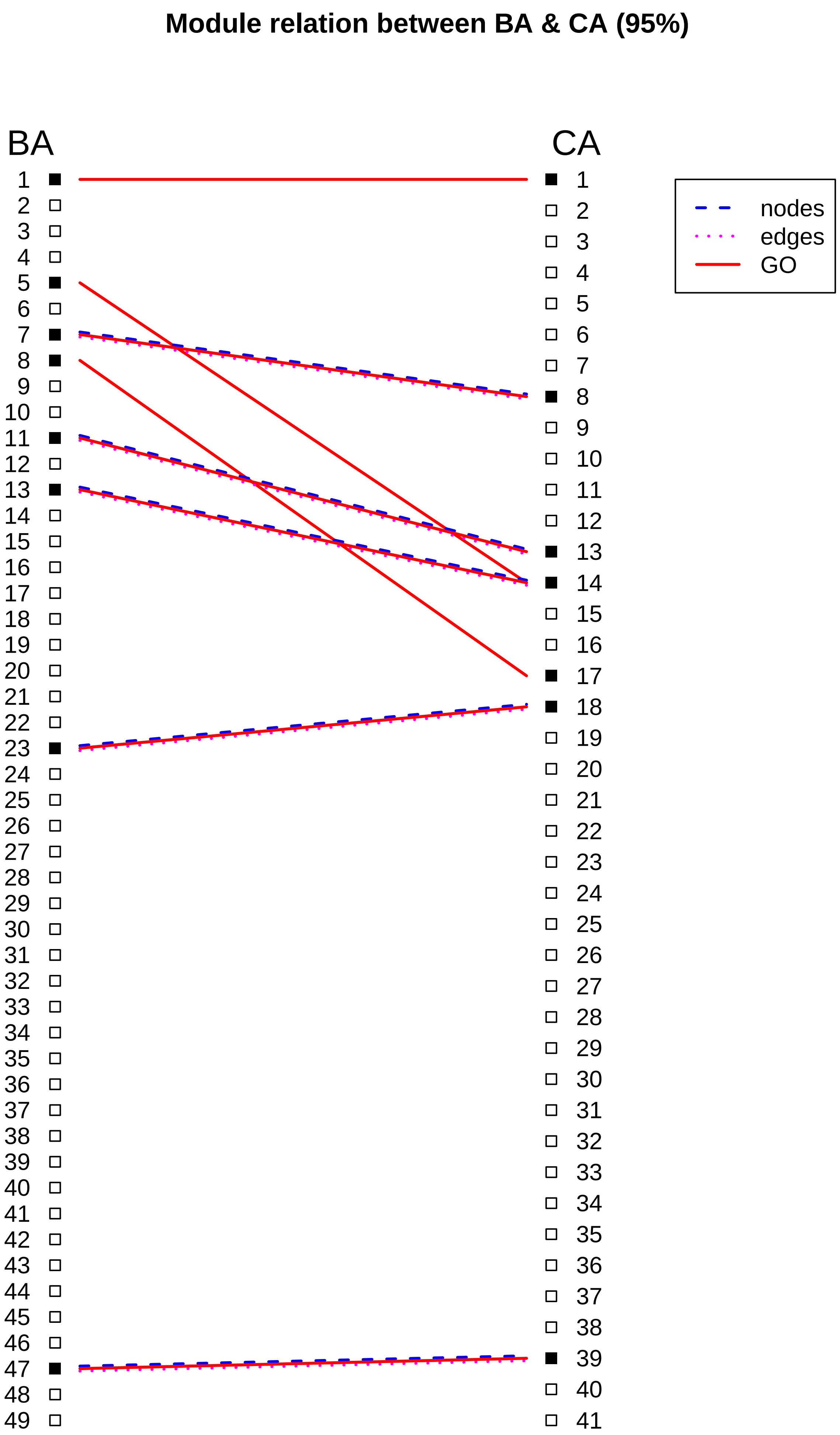
Module relation between BA & CA (95%)
BA
CA
1
2
3
4
5
6
7
8
9
10
11
12
13
14
15
16
17
18
19
20
21
22
23
24
25
26
27
28
29
30
31
32
33
34
35
36
37
38
39
40
41
42
43
44
45
46
47
48
49
1
2
3
4
5
6
7
8
9
10
11
12
13
14
15
16
17
18
19
20
21
22
23
24
25
26
27
28
29
30
31
32
33
34
35
36
37
38
39
40
41
nodes
edges
GO

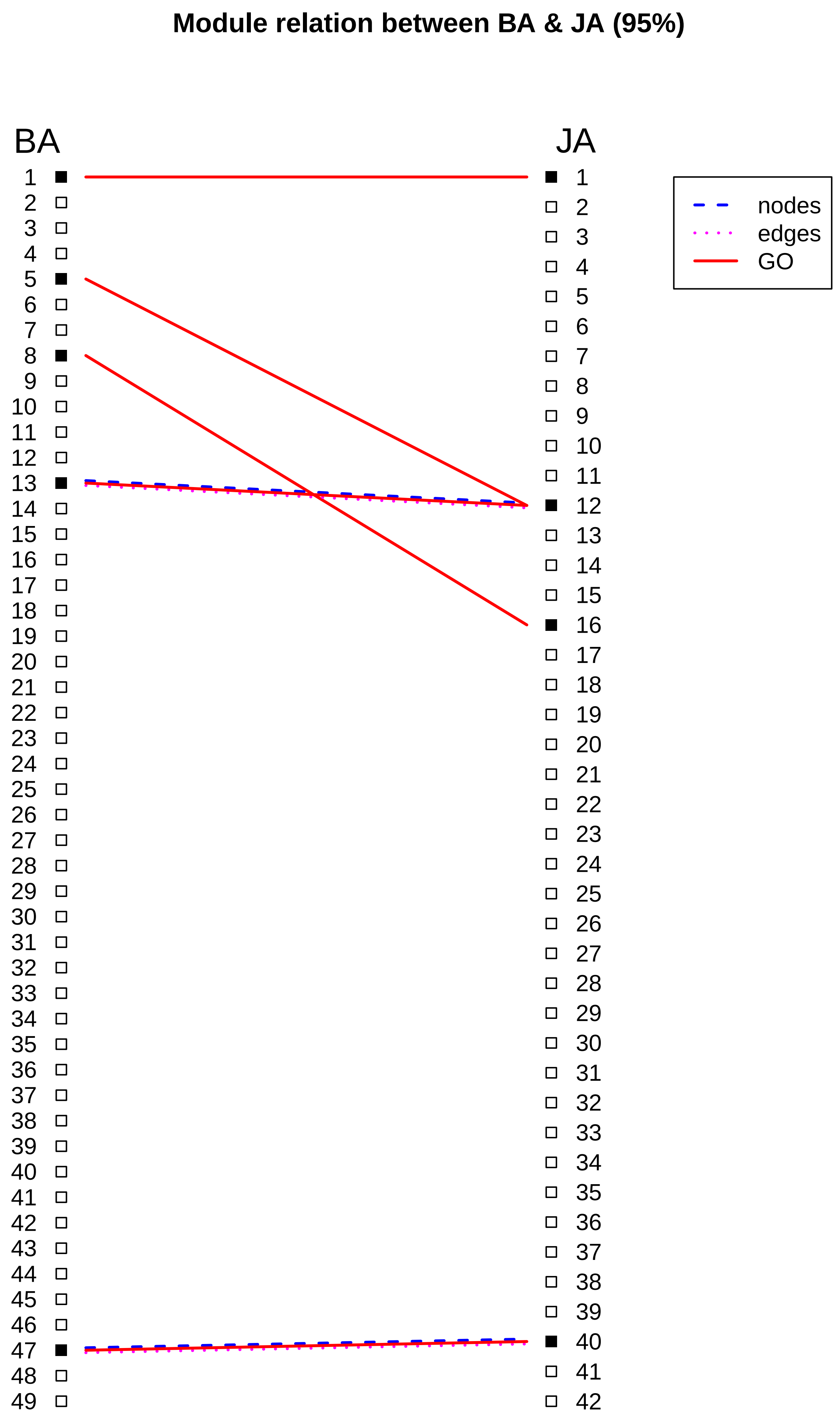
Module relation between BA & JA (95%)
BA
JA
1
2
3
4
5
6
7
8
9
10
11
12
13
14
15
16
17
18
19
20
21
22
23
24
25
26
27
28
29
30
31
32
33
34
35
36
37
38
39
40
41
42
43
44
45
46
47
48
49
1
2
3
4
5
6
7
8
9
10
11
12
13
14
15
16
17
18
19
20
21
22
23
24
25
26
27
28
29
30
31
32
33
34
35
36
37
38
39
40
41
42
nodes
edges
GO

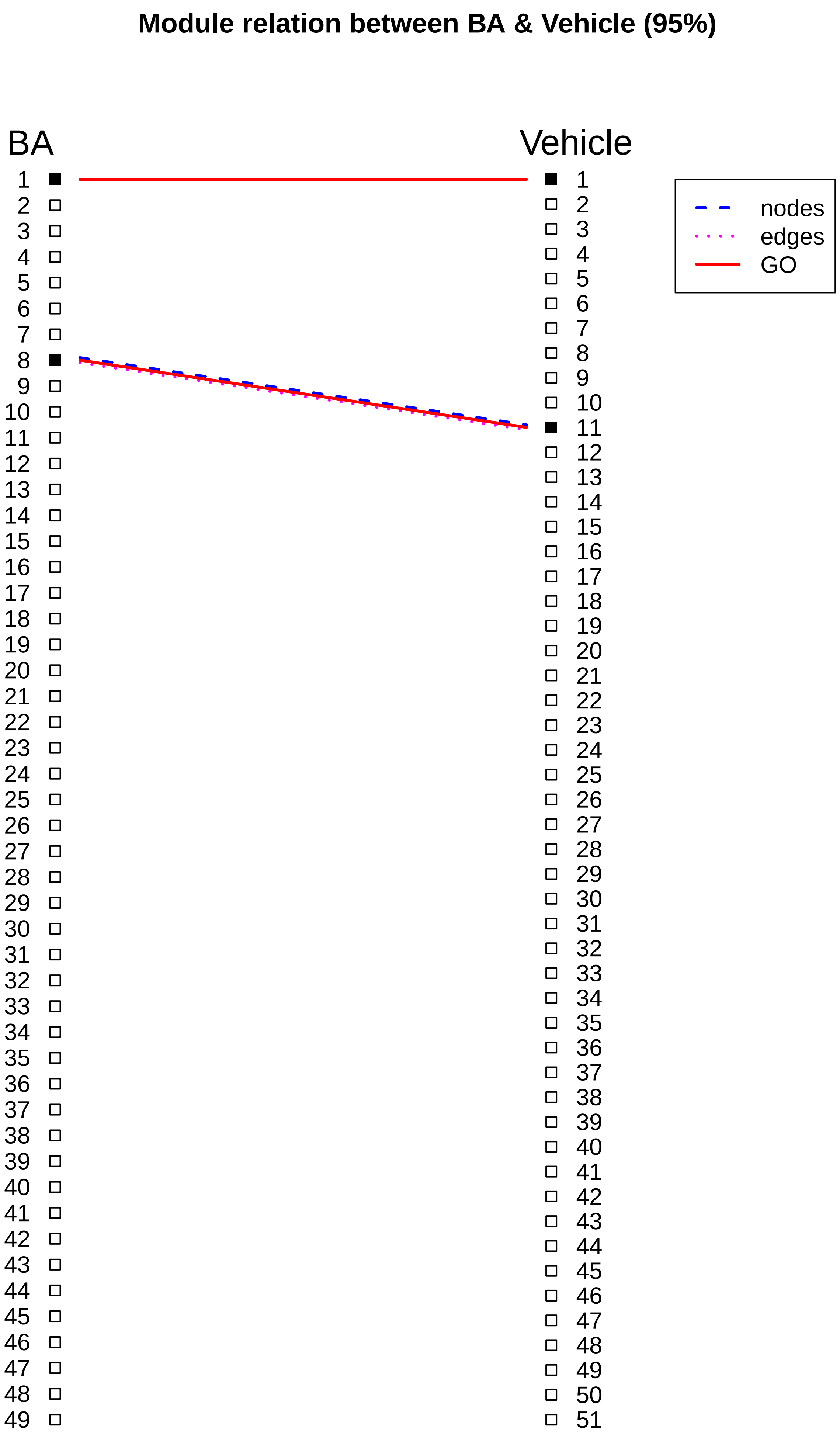
Module relation between BA & Vehicle (95%)
BA
Vehicle
nodes
edges
GO
1 2 3 4 5 6 7 8 9 10 11 12 13 14 15 16 17 18 19 20 21 22 23 24 25 26 27 28 29 30 31 32 33 34 35 36 37 38 39 40 41 42 43 44 45 46 47 48 49
1 2 3 4 5 6 7 8 9 10 11 12 13 14 15 16 17 18 19 20 21 22 23 24 25 26 27 28 29 30 31 32 33 34 35 36 37 38 39 40 41 42 43 44 45 46 47 48 49 50 51

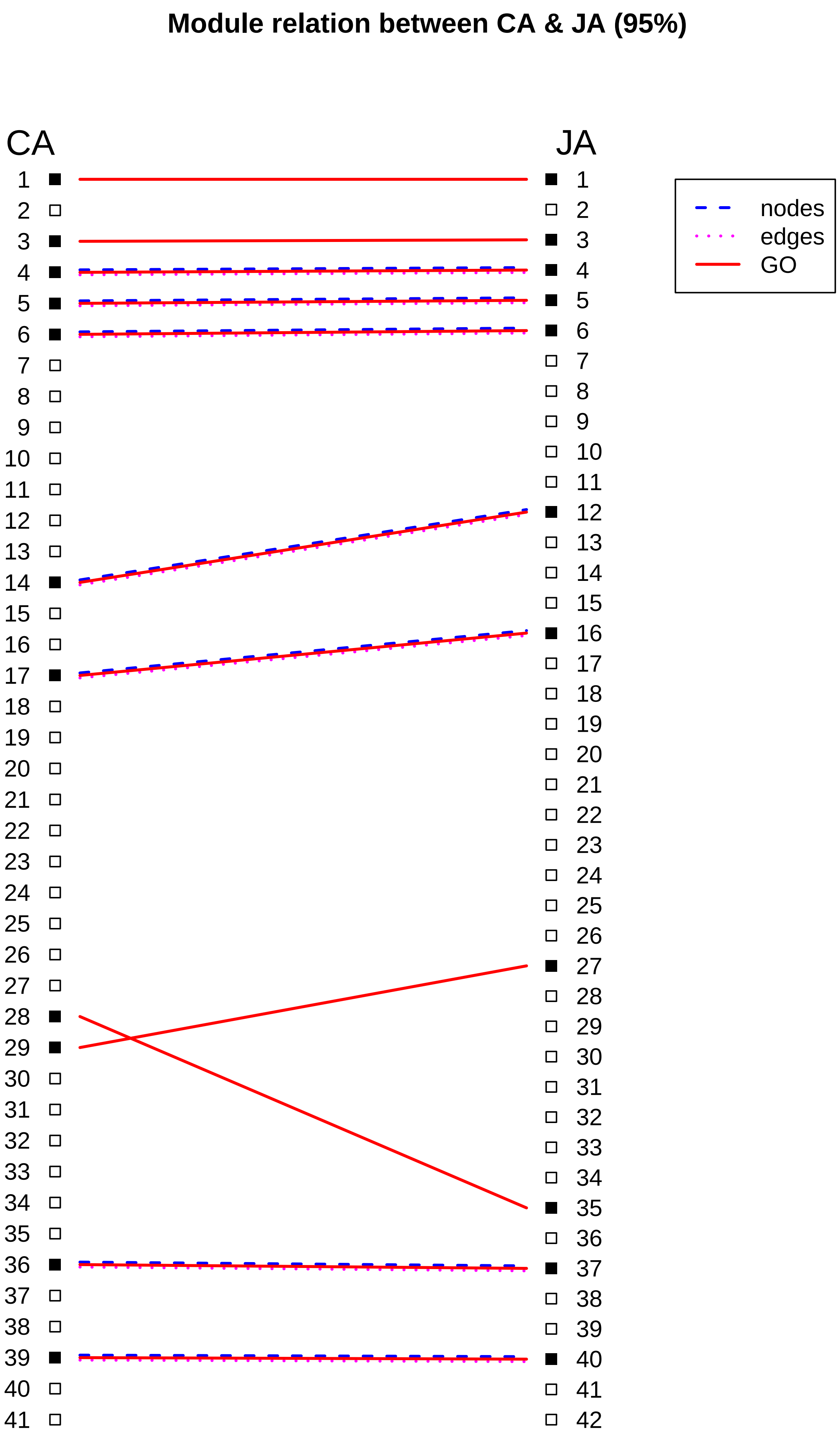
Module relation between CA & JA (95%)
CA
JA
1
2
3
4
5
6
7
8
9
10
11
12
13
14
15
16
17
18
19
20
21
22
23
24
25
26
27
28
29
30
31
32
33
34
35
36
37
38
39
40
41
1
2
3
4
5
6
7
8
9
10
11
12
13
14
15
16
17
18
19
20
21
22
23
24
25
26
27
28
29
30
31
32
33
34
35
36
37
38
39
40
41
42
nodes
edges
GO

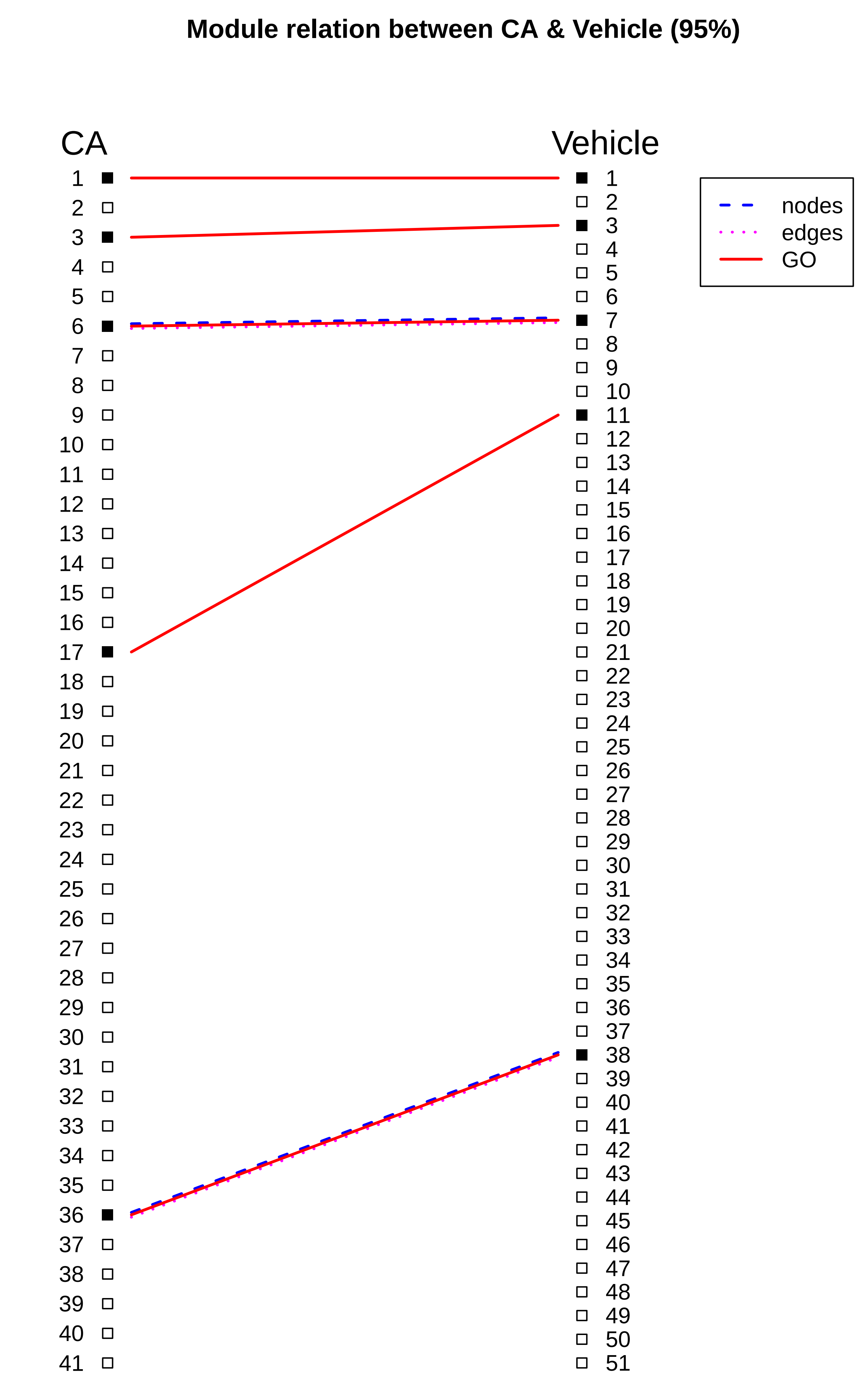
Module relation between CA & Vehicle (95%)
CA
Vehicle
nodes
edges
GO
1 2 3 4 5 6 7 8 9 10 11 12 13 14 15 16 17 18 19 20 21 22 23 24 25 26 27 28 29 30 31 32 33 34 35 36 37 38 39 40 41
1 2 3 4 5 6 7 8 9 10 11 12 13 14 15 16 17 18 19 20 21 22 23 24 25 26 27 28 29 30 31 32 33 34 35 36 37 38 39 40 41 42 43 44 45 46 47 48 49 50 51

Module relation between JA & Vehicle (95%)

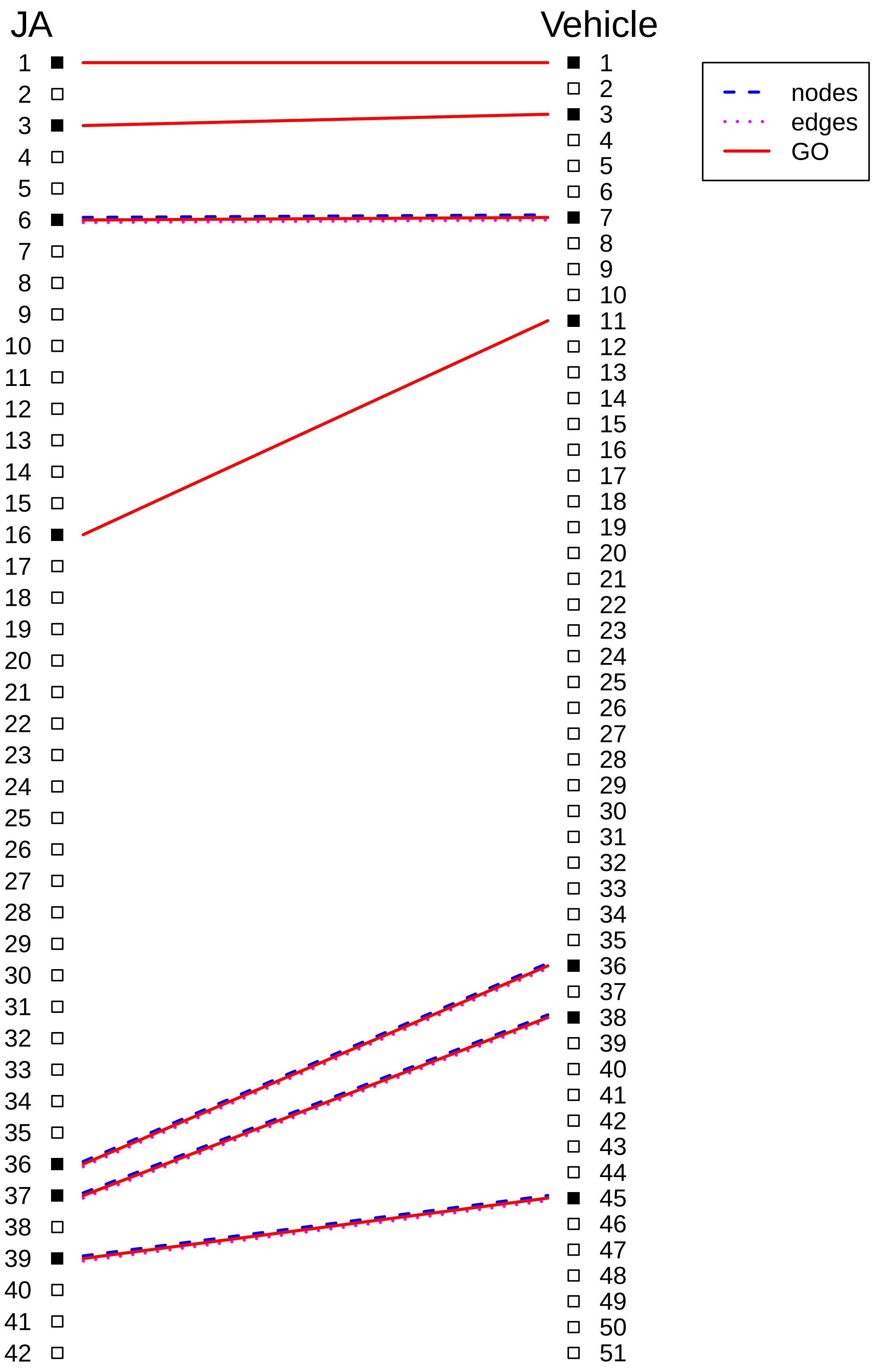

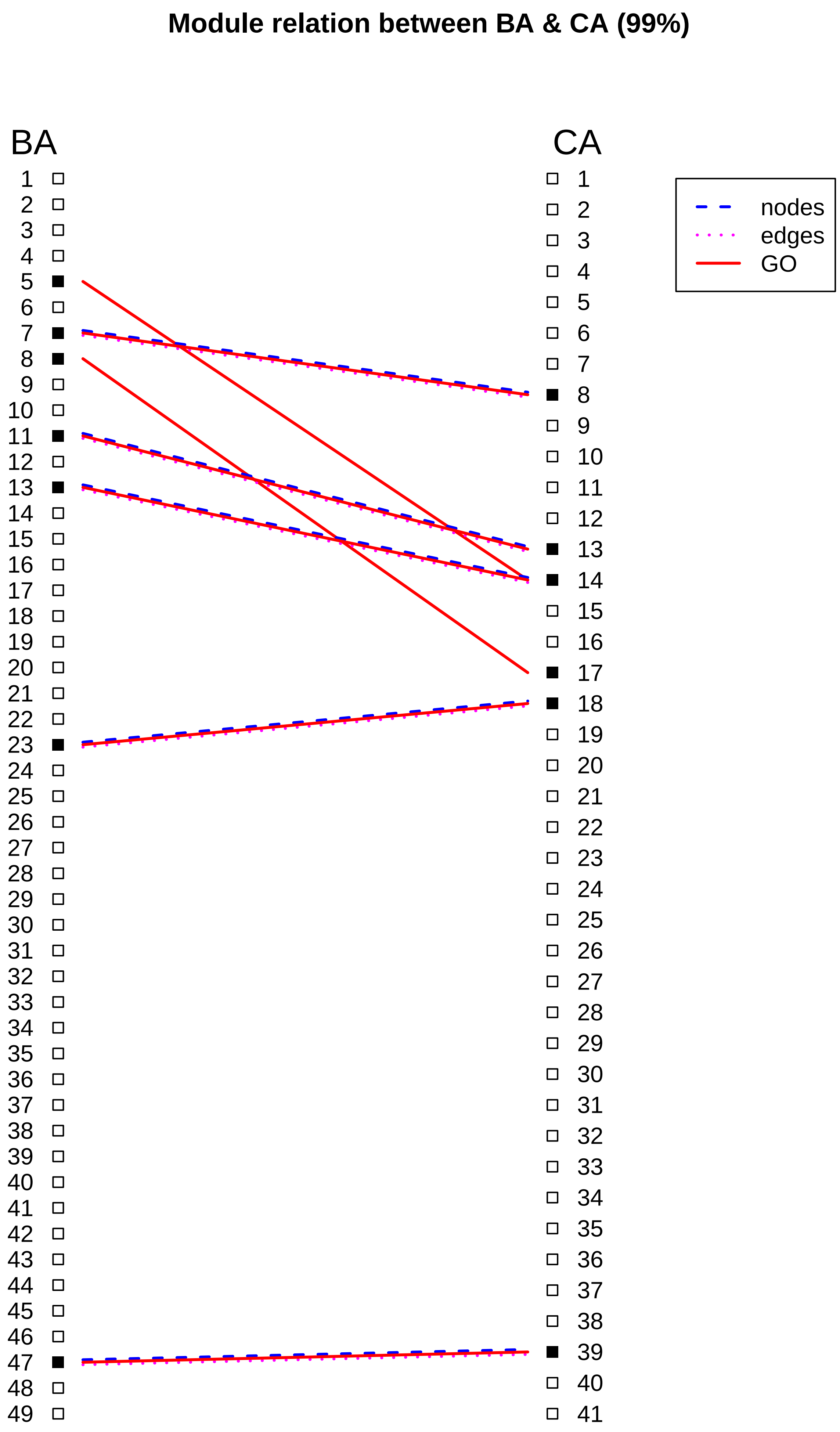
Module relation between BA & CA (99%)
BA
CA
nodes
edges
GO
1
2
3
4
5
6
7
8
9
10
11
12
13
14
15
16
17
18
19
20
21
22
23
24
25
26
27
28
29
30
31
32
33
34
35
36
37
38
39
40
41
42
43
44
45
46
47
48
49

Module relation between BA & JA (99%)

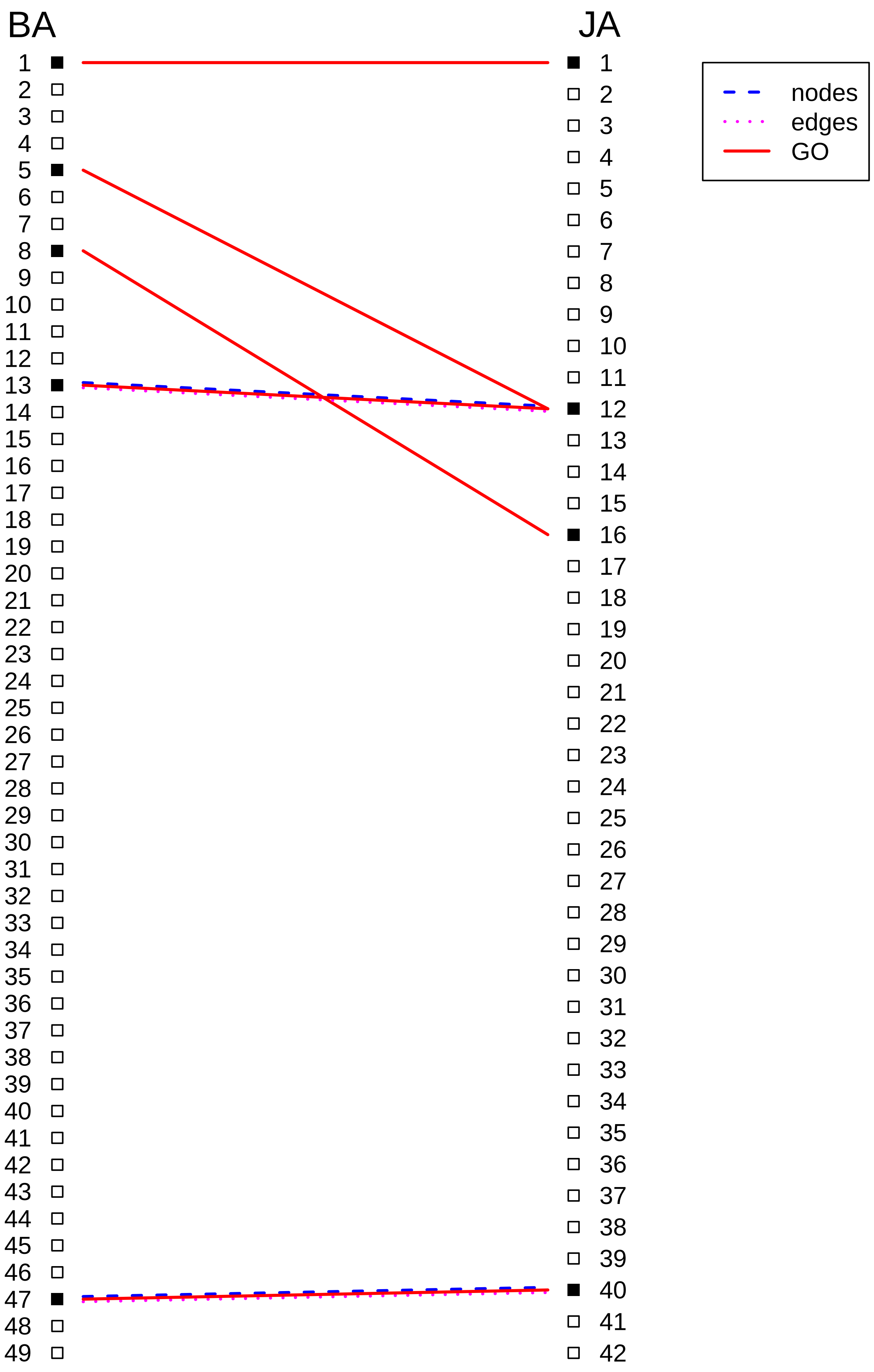

Module relation between BA & Vehicle (99%)

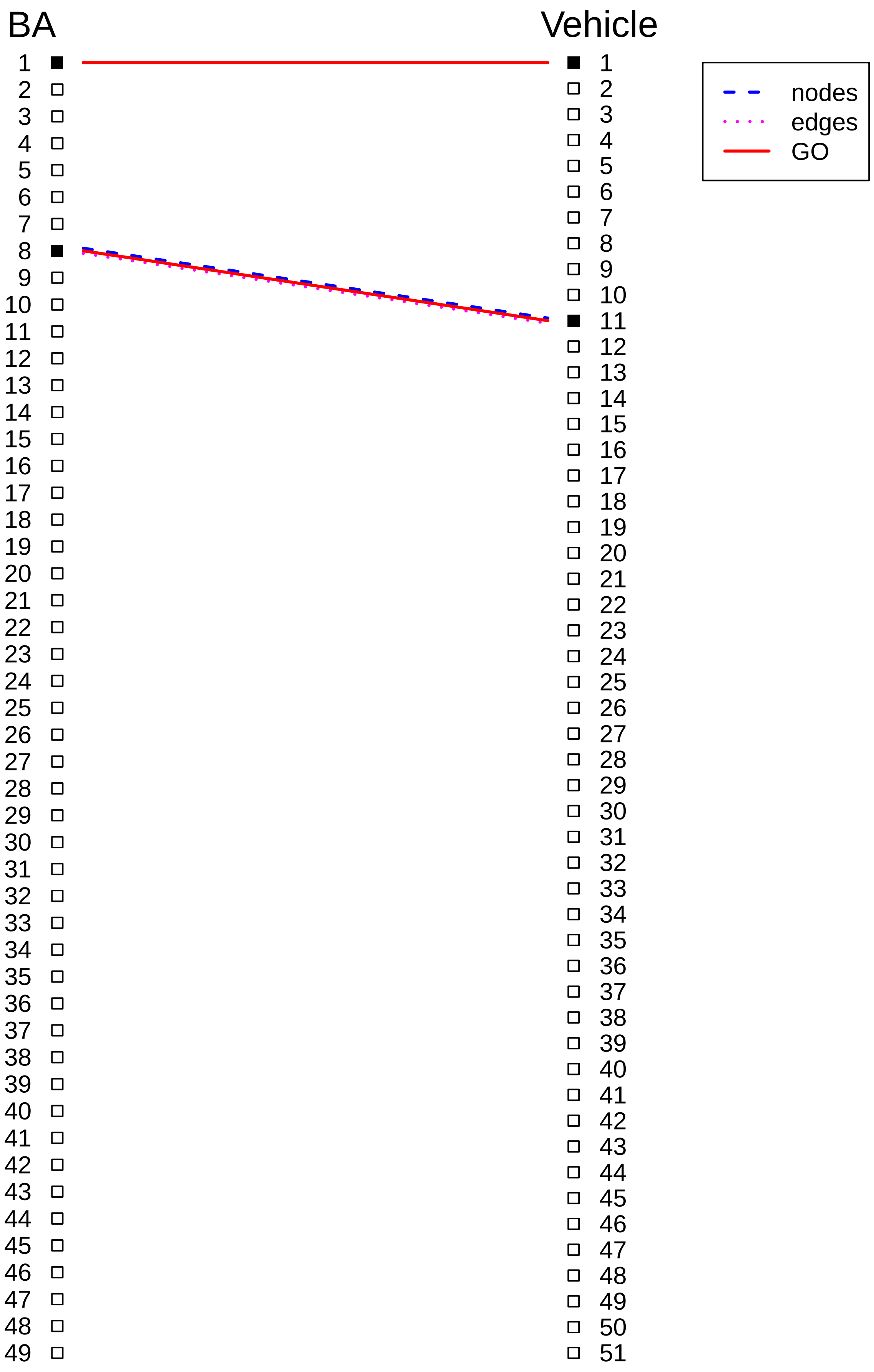

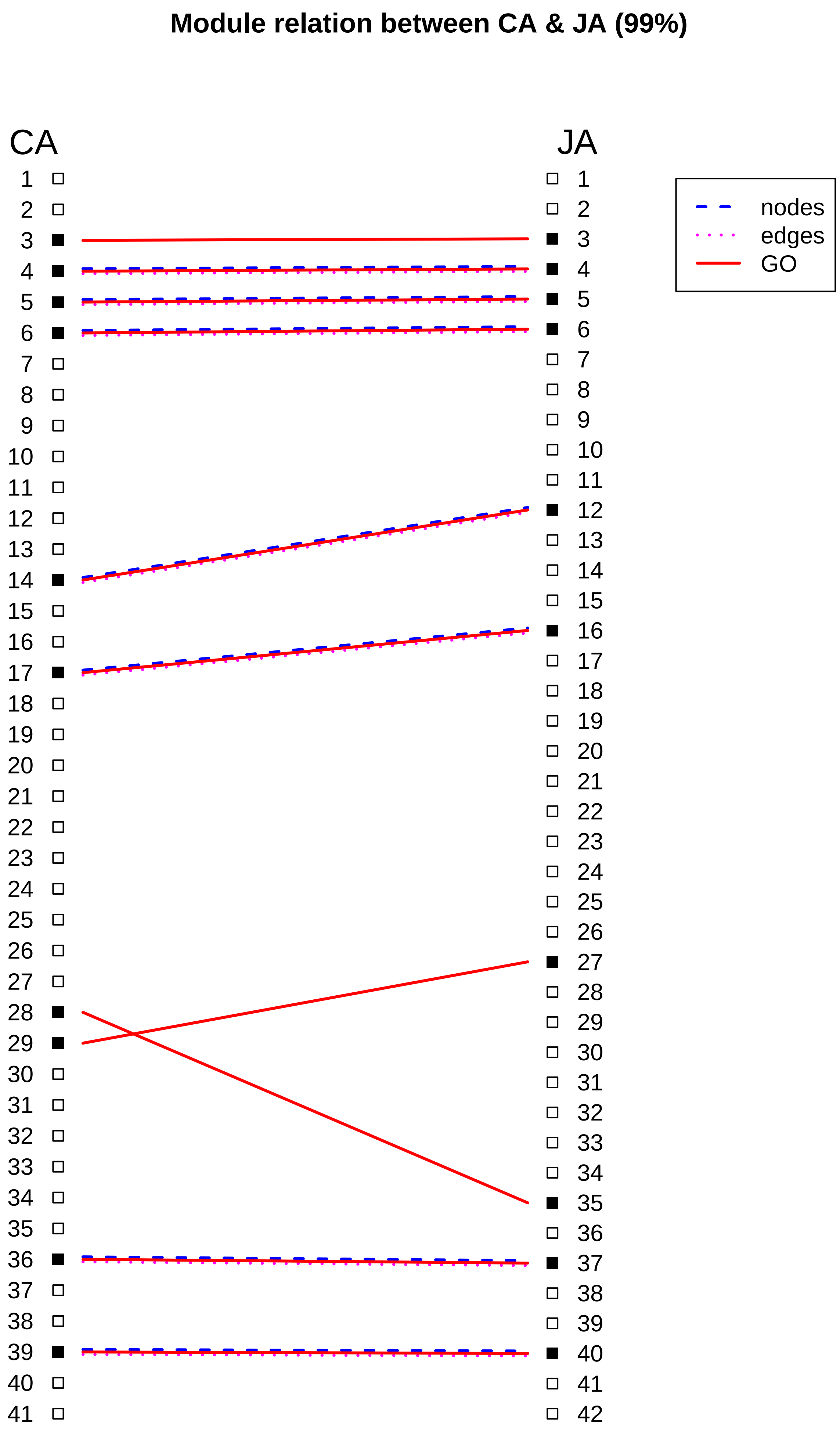

Module relation between CA & JA (99%)
CA
JA
nodes
edges
GO
1
2
3
4
5
6
7
8
9
10
11
12
13
14
15
16
17
18
19
20
21
22
23
24
25
26
27
28
29
30
31
32
33
34
35
36
37
38
39
40
41
42

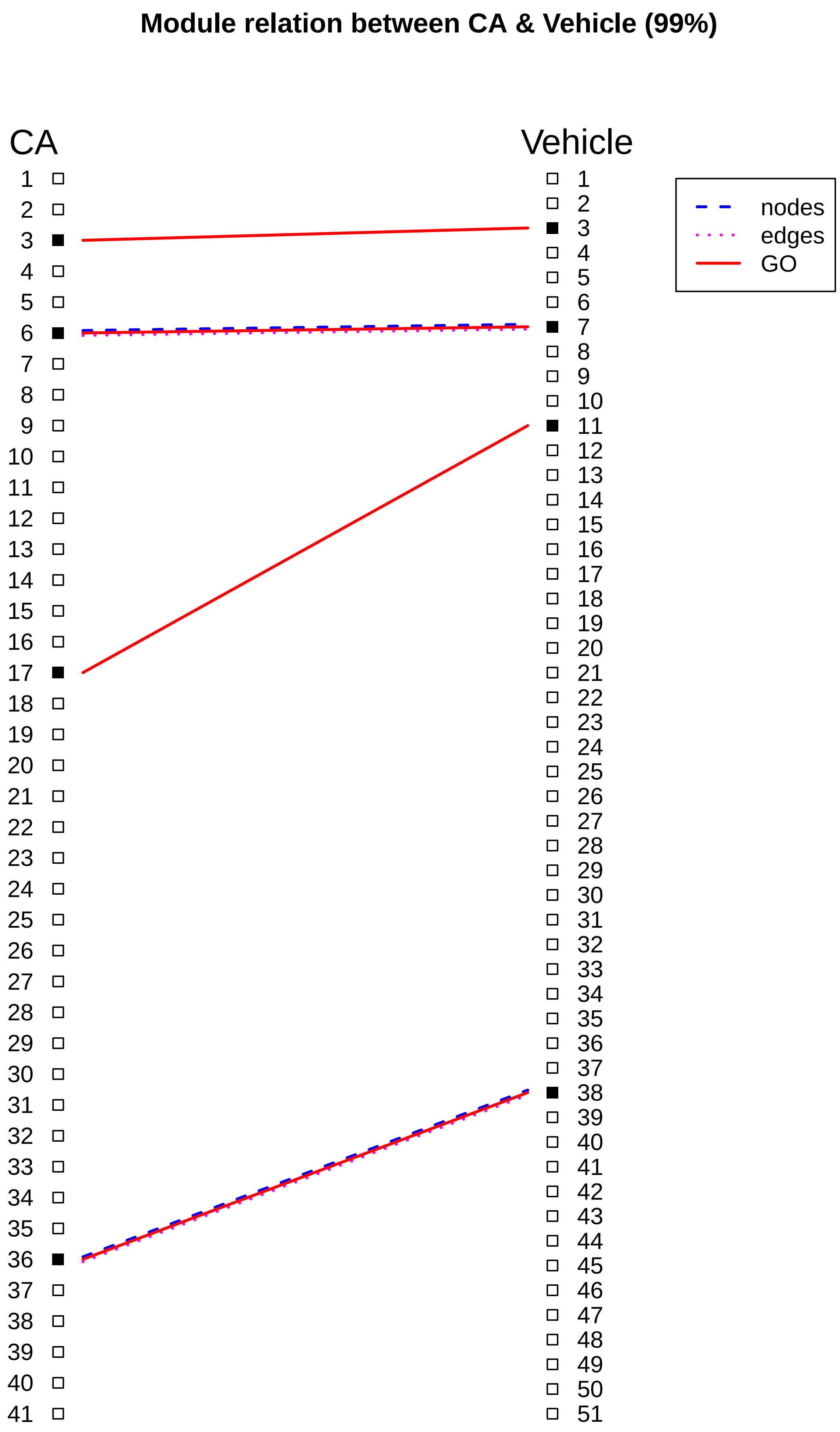

Module relation between CA & Vehicle (99%)
CA
Vehicle
nodes
edges
GO
1
2
3
4
5
6
7
8
9
10
11
12
13
14
15
16
17
18
19
20
21
22
23
24
25
26
27
28
29
30
31
32
33
34
35
36
37
38
39
40
41
42
43
44
45
46
47
48
49
50
51

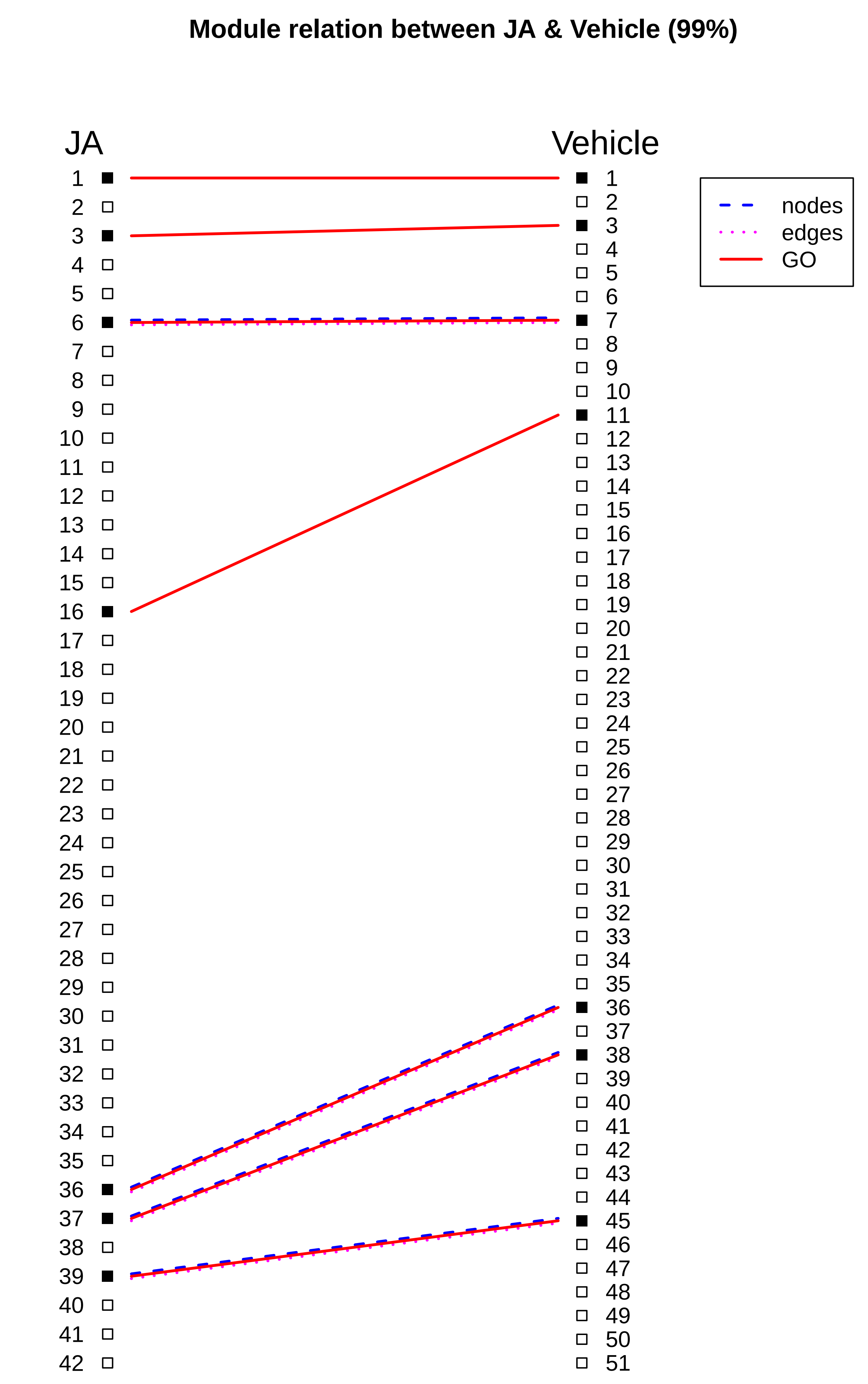
Module relation between JA & Vehicle (99%)
JA
Vehicle
nodes
edges
GO
1 2 3 4 5 6 7 8 9 10 11 12 13 14 15 16 17 18 19 20 21 22 23 24 25 26 27 28 29 30 31 32 33 34 35 36 37 38 39 40 41 42
1 2 3 4 5 6 7 8 9 10 11 12 13 14 15 16 17 18 19 20 21 22 23 24 25 26 27 28 29 30 31 32 33 34 35 36 37 38 39 40 41 42 43 44 45 46 47 48 49 50 51
