# Supplementary material for: Quantitative Determination of Flexible Pharmacological Mechanisms Based On Topological Variation in Mice Anti-Ischemic Modular Networks
Source: PLoS One. 2016 Jul 6;11(7):e0158379. doi: 10.1371/journal.pone.0158379 (PMC4934924; doi:10.1371/journal.pone.0158379)
Supplement: S1 Table — (DOCX) [file pone.0158379.s002.docx]

**S1 Table. Topological attributes of global networks in different groups.**

| **Groups** | **Nodes** | **Edges** | **Network density** | **Clustering coefficient** | **Connected components** | **Network diameter** | **Network radius** | **Network centralization** |
| --- | --- | --- | --- | --- | --- | --- | --- | --- |
| **Vehicle** | 2183 | 6656 | 0.003 | 0.109 | 26 | 12 | 1 | 0.129 |
| **BA** | 2229 | 6738 | 0.003 | 0.102 | 27 | 11 | 1 | 0.128 |
| **CA** | 1981 | 5780 | 0.003 | 0.106 | 25 | 13 | 1 | 0.136 |
| **JA** | 2032 | 5980 | 0.003 | 0.109 | 26 | 12 | 1 | 0.136 |
