# Supplementary material for: Quantitative Determination of Flexible Pharmacological Mechanisms Based On Topological Variation in Mice Anti-Ischemic Modular Networks
Source: PLoS One. 2016 Jul 6;11(7):e0158379. doi: 10.1371/journal.pone.0158379 (PMC4934924; doi:10.1371/journal.pone.0158379)
Supplement: S2 Table — (DOCX) [file pone.0158379.s003.docx]

**S2 Table. Affinity propagation (AP) results for all parameters tested.**

| **Groups** | **Preference** | **Clusters** | **Average size** | **Maximum size** | **Minimum size** | **Modularity** | **Entropy** |
| --- | --- | --- | --- | --- | --- | --- | --- |
| **BA** | 0.1 | 187 | 11.92 | 696 | 1 | 0.147 | 6.85707 |
|  | 0.2 | 187 | 11.92 | 697 | 1 | 0.147 | 6.85824 |
|  | 0.3 | 187 | 11.92 | 696 | 1 | 0.147 | 6.85729 |
|  | 0.4 | 187 | 11.92 | 697 | 1 | 0.146 | 6.8581 |
|  | 0.5 | 187 | 11.92 | 697 | 1 | 0.146 | 6.8581 |
|  | 0.6 | 187 | 11.92 | 696 | 1 | 0.147 | 6.85743 |
|  | 0.7 | 187 | 11.92 | 697 | 1 | 0.147 | 6.85824 |
|  | 0.8 | 187 | 11.92 | 696 | 1 | 0.147 | 6.85729 |
|  | 0.9 | 187 | 11.92 | 697 | 1 | 0.146 | 6.8581 |
|  | 0.95 | 187 | 11.92 | 697 | 1 | 0.147 | 6.85824 |
| **CA** | 0.1 | 141 | 14.05 | 715 | 1 | 0.121 | 6.75786 |
|  | 0.2 | 141 | 14.05 | 714 | 1 | 0.122 | 6.75695 |
|  | 0.3 | 141 | 14.05 | 715 | 1 | 0.121 | 6.75786 |
|  | 0.4 | 141 | 14.05 | 714 | 1 | 0.122 | 6.75695 |
|  | 0.5 | 141 | 14.05 | 715 | 1 | 0.121 | 6.75786 |
|  | 0.6 | 141 | 14.05 | 715 | 1 | 0.121 | 6.75786 |
|  | 0.7 | 141 | 14.05 | 714 | 1 | 0.122 | 6.75695 |
|  | 0.8 | 141 | 14.05 | 715 | 1 | 0.121 | 6.75786 |
|  | 0.9 | 141 | 14.05 | 714 | 1 | 0.122 | 6.75695 |
|  | 0.95 | 141 | 14.05 | 714 | 1 | 0.122 | 6.75695 |
| **JA** | 0.1 | 166 | 12.241 | 657 | 1 | 0.134 | 6.80488 |
|  | 0.2 | 166 | 12.241 | 657 | 1 | 0.134 | 6.80511 |
|  | 0.3 | 166 | 12.241 | 657 | 1 | 0.135 | 6.806 |
|  | 0.4 | 166 | 12.241 | 657 | 1 | 0.135 | 6.806 |
|  | 0.5 | 166 | 12.241 | 657 | 1 | 0.134 | 6.80511 |
|  | 0.6 | 166 | 12.241 | 657 | 1 | 0.134 | 6.80511 |
|  | 0.7 | 166 | 12.241 | 657 | 1 | 0.134 | 6.80511 |
|  | 0.8 | 166 | 12.241 | 657 | 1 | 0.135 | 6.806 |
|  | 0.9 | 166 | 12.241 | 657 | 1 | 0.135 | 6.806 |
|  | 0.95 | 166 | 12.241 | 657 | 1 | 0.134 | 6.80511 |
| **Vehicle** | 0.1 | 181 | 12.061 | 674 | 1 | 0.158 | 6.83255 |
|  | 0.2 | 181 | 12.061 | 674 | 1 | 0.158 | 6.83284 |
|  | 0.3 | 182 | 11.995 | 674 | 1 | 0.158 | 6.83191 |
|  | 0.4 | 182 | 11.995 | 674 | 1 | 0.158 | 6.83219 |
|  | 0.5 | 181 | 12.061 | 674 | 1 | 0.158 | 6.83284 |
|  | 0.6 | 181 | 12.061 | 674 | 1 | 0.158 | 6.83255 |
|  | 0.7 | 181 | 12.061 | 674 | 1 | 0.158 | 6.83284 |
|  | 0.8 | 182 | 11.995 | 674 | 1 | 0.158 | 6.83191 |
|  | 0.9 | 182 | 11.995 | 674 | 1 | 0.158 | 6.83219 |
|  | 0.95 | 181 | 12.061 | 674 | 1 | 0.158 | 6.83284 |
