# Supplementary material for: Quantitative Determination of Flexible Pharmacological Mechanisms Based On Topological Variation in Mice Anti-Ischemic Modular Networks
Source: PLoS One. 2016 Jul 6;11(7):e0158379. doi: 10.1371/journal.pone.0158379 (PMC4934924; doi:10.1371/journal.pone.0158379)
Supplement: S3 Table — (DOCX) [file pone.0158379.s004.docx]

**S3 Table. MCL results for all parameters tested.**

| **Groups** | **Inflation** | **Clusters** | **Average size** | **Maximum size** | **Minimum size** | **Modularity** | **Entropy** |
| --- | --- | --- | --- | --- | --- | --- | --- |
| **BA** | 1.5 | 107 | 20.832 | 1779 | 2 | 0.04 | 7.06837 |
|  | 1.8 | 412 | 5.294 | 286 | 2 | 0.324 | 7.17784 |
|  | 2 | 508 | 4.205 | 168 | 2 | 0.286 | 7.25503 |
|  | 2.5 | 607 | 3.292 | 118 | 2 | 0.235 | 7.24264 |
|  | 3 | 642 | 2.975 | 94 | 2 | 0.202 | 7.26574 |
|  | 3.5 | 631 | 2.867 | 85 | 2 | 0.182 | 7.27564 |
|  | 4 | 620 | 2.823 | 81 | 2 | 0.173 | 7.21025 |
|  | 4.5 | 609 | 2.782 | 78 | 2 | 0.164 | 7.2589 |
|  | 5 | 591 | 2.763 | 78 | 2 | 0.155 | 7.15933 |
| **CA** | 1.5 | 89 | 22.258 | 1629 | 2 | 0.014 | 6.96415 |
|  | 1.8 | 371 | 5.218 | 274 | 2 | 0.304 | 7.07877 |
|  | 2 | 437 | 4.309 | 160 | 2 | 0.293 | 7.12247 |
|  | 2.5 | 549 | 3.209 | 113 | 2 | 0.233 | 7.19854 |
|  | 3 | 577 | 2.919 | 93 | 2 | 0.205 | 7.1687 |
|  | 3.5 | 571 | 2.834 | 85 | 2 | 0.186 | 7.18417 |
|  | 4 | 552 | 2.783 | 81 | 2 | 0.175 | 7.14589 |
|  | 4.5 | 538 | 2.76 | 79 | 2 | 0.165 | 7.15504 |
|  | 5 | 528 | 2.733 | 77 | 2 | 0.158 | 7.03055 |
| **JA** | 1.5 | 95 | 21.389 | 1687 | 2 | 0.007 | 6.97995 |
|  | 1.8 | 394 | 5.043 | 303 | 2 | 0.297 | 7.07195 |
|  | 2 | 462 | 4.193 | 170 | 2 | 0.284 | 7.15808 |
|  | 2.5 | 569 | 3.193 | 111 | 2 | 0.229 | 7.18239 |
|  | 3 | 588 | 2.923 | 86 | 2 | 0.201 | 7.20059 |
|  | 3.5 | 585 | 2.843 | 80 | 2 | 0.187 | 7.19467 |
|  | 4 | 568 | 2.803 | 77 | 2 | 0.177 | 7.17821 |
|  | 4.5 | 555 | 2.768 | 74 | 2 | 0.167 | 7.17414 |
|  | 5 | 543 | 2.753 | 74 | 2 | 0.161 | 7.0725 |
| **Vehicle** | 1.5 | 97 | 22.505 | 1839 | 2 | 0.011 | 7.05278 |
|  | 1.8 | 415 | 5.142 | 311 | 2 | 0.289 | 7.15601 |
|  | 2 | 494 | 4.178 | 179 | 2 | 0.283 | 7.18571 |
|  | 2.5 | 596 | 3.28 | 123 | 2 | 0.226 | 7.25266 |
|  | 3 | 623 | 2.995 | 96 | 2 | 0.2 | 7.2747 |
|  | 3.5 | 612 | 2.904 | 83 | 2 | 0.182 | 7.22857 |
|  | 4 | 600 | 2.835 | 79 | 2 | 0.172 | 7.25636 |
|  | 4.5 | 589 | 2.796 | 76 | 2 | 0.164 | 7.20077 |
|  | 5 | 577 | 2.776 | 75 | 2 | 0.156 | 7.23938 |
