# Supplementary material for: Quantitative Determination of Flexible Pharmacological Mechanisms Based On Topological Variation in Mice Anti-Ischemic Modular Networks
Source: PLoS One. 2016 Jul 6;11(7):e0158379. doi: 10.1371/journal.pone.0158379 (PMC4934924; doi:10.1371/journal.pone.0158379)
Supplement: S4 Table — (DOCX) [file pone.0158379.s005.docx]

**S4 Table. MCODE results for all parameters tested.**

| **Groups** | **Parameters ^a^** | **Clusters** | **Average size** | **Maximum size** | **Minimum size** | **Modularity** | **Entropy** |
| --- | --- | --- | --- | --- | --- | --- | --- |
| **BA** | 1 | 31 | 10.323 | 46 | 3 | 0.064 | 5.55785 |
|  | 2 | 40 | 7.15 | 33 | 3 | 0.077 | 5.43721 |
|  | 3 | 41 | 6.561 | 24 | 3 | 0.077 | 5.37963 |
|  | **4** | **49** | **5.327** | **19** | **3** | **0.077** | **5.33077** |
|  | 5 | 31 | 58.677 | 389 | 3 | 0.146 | 6.4269 |
|  | 6 | 40 | 41.9 | 284 | 3 | 0.237 | 6.30021 |
|  | 7 | 41 | 37.146 | 318 | 3 | 0.214 | 6.22903 |
|  | 8 | 49 | 26.837 | 182 | 3 | 0.226 | 6.22418 |
|  | 9 | 31 | 15.065 | 57 | 3 | 0.073 | 5.8719 |
|  | 10 | 40 | 10.175 | 44 | 3 | 0.087 | 5.72386 |
|  | 11 | 41 | 9.098 | 32 | 3 | 0.086 | 5.63819 |
|  | 12 | 49 | 7.122 | 25 | 3 | 0.079 | 5.55695 |
|  | 13 | 31 | 73.065 | 389 | 4 | 0.173 | 6.64938 |
|  | 14 | 40 | 53.875 | 284 | 4 | 0.282 | 6.537 |
|  | 15 | 41 | 49.902 | 332 | 4 | 0.264 | 6.48761 |
|  | 16 | 49 | 41.857 | 351 | 3 | 0.265 | 6.43646 |
| **CA** | 1 | 30 | 8.967 | 58 | 3 | 0.054 | 5.3891 |
|  | 2 | 39 | 6.513 | 31 | 3 | 0.074 | 5.31087 |
|  | **3** | **41** | **6.171** | **24** | **3** | **0.079** | **5.3029** |
|  | 4 | 45 | 5.333 | 13 | 3 | 0.076 | 5.40279 |
|  | 5 | 30 | 52.533 | 316 | 4 | 0.086 | 6.33974 |
|  | 6 | 39 | 39.821 | 259 | 4 | 0.208 | 6.23593 |
|  | 7 | 41 | 36.659 | 259 | 3 | 0.219 | 6.19395 |
|  | 8 | 45 | 32.689 | 259 | 4 | 0.233 | 6.28491 |
|  | 9 | 30 | 14.533 | 81 | 3 | 0.063 | 5.80669 |
|  | 10 | 39 | 9.744 | 49 | 3 | 0.086 | 5.65487 |
|  | 11 | 41 | 8.634 | 35 | 3 | 0.09 | 5.63574 |
|  | 12 | 45 | 7.2 | 26 | 3 | 0.085 | 5.51539 |
|  | 13 | 30 | 70.5 | 387 | 4 | 0.117 | 6.61254 |
|  | 14 | 39 | 52.923 | 306 | 4 | 0.261 | 6.48123 |
|  | 15 | 41 | 47.634 | 259 | 4 | 0.27 | 6.44142 |
|  | 16 | 45 | 41.511 | 259 | 4 | 0.273 | 6.3843 |
| **JA** | 1 | 29 | 10.655 | 56 | 3 | 0.071 | 5.51311 |
|  | 2 | 38 | 7.079 | 29 | 3 | 0.083 | 5.36913 |
|  | **3** | **42** | **5.738** | **16** | **3** | **0.079** | **5.24152** |
|  | 4 | 47 | 5.319 | 16 | 3 | 0.08 | 5.29871 |
|  | 5 | 29 | 61.966 | 285 | 4 | 0.167 | 6.40842 |
|  | 6 | 38 | 44.368 | 265 | 3 | 0.222 | 6.2864 |
|  | 7 | 42 | 34.69 | 265 | 4 | 0.209 | 6.14228 |
|  | 8 | 47 | 31.915 | 265 | 4 | 0.228 | 6.18335 |
|  | 9 | 29 | 16.414 | 141 | 3 | 0.069 | 5.87226 |
|  | 10 | 38 | 10.237 | 48 | 3 | 0.093 | 5.6695 |
|  | 11 | 42 | 9.048 | 52 | 3 | 0.088 | 5.61422 |
|  | 12 | 47 | 7.787 | 51 | 3 | 0.088 | 5.60606 |
|  | 13 | 29 | 80.172 | 417 | 4 | 0.173 | 6.65263 |
|  | 14 | 38 | 56.763 | 290 | 4 | 0.264 | 6.51973 |
|  | 15 | 42 | 52.857 | 265 | 4 | 0.271 | 6.51411 |
|  | 16 | 47 | 46.298 | 265 | 4 | 0.288 | 6.49106 |
| **Vehicle** | 1 | 37 | 8.973 | 61 | 3 | 0.065 | 5.57459 |
|  | 2 | 48 | 6.708 | 40 | 3 | 0.078 | 5.54359 |
|  | **3** | **51** | **5.863** | **25** | **3** | **0.078** | **5.44995** |
|  | 4 | 49 | 6.143 | 23 | 3 | 0.078 | 5.48124 |
|  | 5 | 37 | 50.784 | 417 | 4 | 0.148 | 6.47533 |
|  | 6 | 48 | 39.479 | 360 | 4 | 0.201 | 6.42668 |
|  | 7 | 51 | 35.902 | 328 | 4 | 0.232 | 6.38271 |
|  | 8 | 49 | 35.816 | 330 | 4 | 0.209 | 6.36695 |
|  | 9 | 37 | 13.784 | 82 | 3 | 0.073 | 5.93583 |
|  | 10 | 48 | 11.083 | 45 | 3 | 0.097 | 5.97695 |
|  | 11 | 51 | 9.059 | 34 | 3 | 0.093 | 5.81893 |
|  | 12 | 49 | 8.694 | 51 | 3 | 0.085 | 5.75644 |
|  | 13 | 37 | 66.541 | 417 | 4 | 0.173 | 6.70974 |
|  | 14 | 48 | 55.479 | 373 | 4 | 0.27 | 6.74213 |
|  | 15 | 51 | 49.255 | 328 | 4 | 0.303 | 6.67981 |
|  | 16 | 49 | 48.837 | 330 | 4 | 0.255 | 6.61472 |

^a^ In the MCODE algorithm, we tried all possible combinations of the key parameters:

- Parameters 1: Include Loops: false; Degree Cutoff: 3; Node Score Cutoff: 0.2; Haircut: true; Fluff: false; K-Core: 2; Max. Depth from Seed: 100
- Parameters 2: Include Loops: false; Degree Cutoff: 3; Node Score Cutoff: 0.2; Haircut: true; Fluff: false; K-Core: 2; Max. Depth from Seed: 5
- Parameters 3: Include Loops: false; Degree Cutoff: 3; Node Score Cutoff: 0.2; Haircut: true; Fluff: false; K-Core: 2; Max. Depth from Seed: 4
- Parameters 4: Include Loops: false; Degree Cutoff: 3; Node Score Cutoff: 0.2; Haircut: true; Fluff: false; K-Core: 2; Max. Depth from Seed: 3
- Parameters 5: Include Loops: false; Degree Cutoff: 3; Node Score Cutoff: 0.2; Haircut: true; Fluff: true; Fluff Density Cutoff 0.1; K-Core: 2; Max. Depth from Seed: 100
- Parameters 6: Include Loops: false; Degree Cutoff: 3; Node Score Cutoff: 0.2; Haircut: true; Fluff: true; Fluff Density Cutoff 0.1; K-Core: 2; Max. Depth from Seed: 5
- Parameters 7: Include Loops: false; Degree Cutoff: 3; Node Score Cutoff: 0.2; Haircut: true; Fluff: true; Fluff Density Cutoff 0.1; K-Core: 2; Max. Depth from Seed: 4
- Parameters 8: Include Loops: false; Degree Cutoff: 3; Node Score Cutoff: 0.2; Haircut: true; Fluff: true; Fluff Density Cutoff 0.1; K-Core: 2; Max. Depth from Seed: 3
- Parameters 9: Include Loops: false; Degree Cutoff: 3; Node Score Cutoff: 0.2; Haircut: false; Fluff: false; K-Core: 2; Max. Depth from Seed: 100
- Parameters 10: Include Loops: false; Degree Cutoff: 3; Node Score Cutoff: 0.2; Haircut: false; Fluff: false; K-Core: 2; Max. Depth from Seed: 5
- Parameters 11: Include Loops: false; Degree Cutoff: 3; Node Score Cutoff: 0.2; Haircut: false; Fluff: false; K-Core: 2; Max. Depth from Seed: 4
- Parameters 12: Include Loops: false; Degree Cutoff: 3; Node Score Cutoff: 0.2; Haircut: false; Fluff: false; K-Core: 2; Max. Depth from Seed: 3
- Parameters 13: Include Loops: false; Degree Cutoff: 3; Node Score Cutoff: 0.2; Haircut: false; Fluff: true; K-Core: 2; Max. Depth from Seed: 100
- Parameters 14: Include Loops: false; Degree Cutoff: 3; Node Score Cutoff: 0.2; Haircut: false; Fluff: true; K-Core: 2; Max. Depth from Seed: 5
- Parameters 15: Include Loops: false; Degree Cutoff: 3; Node Score Cutoff: 0.2; Haircut: false; Fluff: true; K-Core: 2; Max. Depth from Seed: 4
- Parameters 16: Include Loops: false; Degree Cutoff: 3; Node Score Cutoff: 0.2; Haircut: false; Fluff: true; K-Core: 2; Max. Depth from Seed: 3
