# Supplementary material for: Quantitative Determination of Flexible Pharmacological Mechanisms Based On Topological Variation in Mice Anti-Ischemic Modular Networks
Source: PLoS One. 2016 Jul 6;11(7):e0158379. doi: 10.1371/journal.pone.0158379 (PMC4934924; doi:10.1371/journal.pone.0158379)
Supplement: S5 Table — (DOCX) [file pone.0158379.s006.docx]

**S5 Table. Functional modules identified by MCODE in different groups.**

| **Groups** | **Clusters** | **Average size** | **Maximum size** | **Minimum size** | **Modularity** | **Entropy** |
| --- | --- | --- | --- | --- | --- | --- |
| **Vehicle** | 50 | 5.863 | 25 | 3 | 0.078 | 5.44995 |
| **BA** | 49 | 5.327 | 19 | 3 | 0.077 | 5.33077 |
| **CA** | 41 | 6.171 | 24 | 3 | 0.079 | 5.3029 |
| **JA** | 42 | 5.738 | 16 | 3 | 0.079 | 5.24152 |
