# Supplementary material for: Quantitative Determination of Flexible Pharmacological Mechanisms Based On Topological Variation in Mice Anti-Ischemic Modular Networks
Source: PLoS One. 2016 Jul 6;11(7):e0158379. doi: 10.1371/journal.pone.0158379 (PMC4934924; doi:10.1371/journal.pone.0158379)
Supplement: S6 Table — (DOCX) [file pone.0158379.s007.docx]

**S6 Table. 218 significantly enriched GO biological processes.**

| **GO biological processes** | **BA^⊙^** | **CA^⊙^** | **JA^⊙^** | **Vehicle^⊙^** |
| --- | --- | --- | --- | --- |
| GO:0000184~nuclear-transcribed mRNA catabolic process, nonsense-mediated decay | 1 | 1 | 1 | 0 |
| GO:0000375~RNA splicing, via transesterification reactions | 1 | 1 | 1 | 1 |
| GO:0000377~RNA splicing, via transesterification reactions with bulged adenosine as nucleophile | 1 | 1 | 1 | 1 |
| GO:0000398~nuclear mRNA splicing, via spliceosome | 1 | 1 | 1 | 1 |
| GO:0000956~nuclear-transcribed mRNA catabolic process | 1 | 1 | 1 | 0 |
| GO:0001505~regulation of neurotransmitter levels | 1 | 1 | 1 | 0 |
| GO:0001835~blastocyst hatching | 0 | 1 | 1 | 0 |
| GO:0002250~adaptive immune response | 0 | 0 | 1 | 1 |
| GO:0002252~immune effector process | 0 | 0 | 1 | 1 |
| GO:0002253~activation of immune response | 0 | 0 | 1 | 1 |
| GO:0002376~immune system process | 0 | 0 | 2 | 2 |
| GO:0002443~leukocyte mediated immunity | 0 | 0 | 1 | 1 |
| GO:0002449~lymphocyte mediated immunity | 0 | 0 | 1 | 1 |
| GO:0002455~humoral immune response mediated by circulating immunoglobulin | 0 | 0 | 1 | 1 |
| GO:0002460~adaptive immune response based on somatic recombination of immune receptors built from immunoglobulin superfamily domains | 0 | 0 | 1 | 1 |
| GO:0002526~acute inflammatory response | 0 | 0 | 1 | 1 |
| GO:0002541~activation of plasma proteins involved in acute inflammatory response | 0 | 0 | 1 | 1 |
| GO:0002682~regulation of immune system process | 0 | 0 | 1 | 1 |
| GO:0002684~positive regulation of immune system process | 0 | 0 | 1 | 1 |
| GO:0003001~generation of a signal involved in cell-cell signaling | 1 | 1 | 1 | 0 |
| GO:0006084~acetyl-CoA metabolic process | 1 | 1 | 1 | 1 |
| GO:0006091~generation of precursor metabolites and energy | 3 | 3 | 2 | 2 |
| GO:0006099~tricarboxylic acid cycle | 1 | 1 | 1 | 1 |
| GO:0006119~oxidative phosphorylation | 1 | 1 | 1 | 1 |
| GO:0006139~nucleobase, nucleoside, nucleotide and nucleic acid metabolic process | 4 | 3 | 4 | 5 |
| GO:0006163~purine nucleotide metabolic process | 1 | 1 | 1 | 2 |
| GO:0006164~purine nucleotide biosynthetic process | 1 | 1 | 1 | 1 |
| GO:0006259~DNA metabolic process | 1 | 1 | 1 | 1 |
| GO:0006281~DNA repair | 1 | 1 | 1 | 1 |
| GO:0006289~nucleotide-excision repair | 1 | 1 | 1 | 1 |
| GO:0006325~chromatin organization | 0 | 1 | 1 | 1 |
| GO:0006350~transcription | 2 | 2 | 2 | 2 |
| GO:0006351~transcription, DNA-dependent | 2 | 2 | 2 | 1 |
| GO:0006364~rRNA processing | 0 | 0 | 2 | 3 |
| GO:0006366~transcription from RNA polymerase II promoter | 1 | 1 | 1 | 1 |
| GO:0006396~RNA processing | 3 | 2 | 3 | 4 |
| GO:0006397~mRNA processing | 2 | 2 | 2 | 1 |
| GO:0006399~tRNA metabolic process | 1 | 0 | 1 | 1 |
| GO:0006401~RNA catabolic process | 1 | 1 | 1 | 0 |
| GO:0006402~mRNA catabolic process | 1 | 1 | 1 | 0 |
| GO:0006403~RNA localization | 1 | 1 | 1 | 1 |
| GO:0006412~translation | 1 | 1 | 1 | 1 |
| GO:0006457~protein folding | 1 | 1 | 1 | 1 |
| GO:0006473~protein amino acid acetylation | 0 | 0 | 0 | 1 |
| GO:0006508~proteolysis | 1 | 0 | 1 | 1 |
| GO:0006732~coenzyme metabolic process | 1 | 1 | 1 | 1 |
| GO:0006753~nucleoside phosphate metabolic process | 1 | 1 | 1 | 2 |
| GO:0006754~ATP biosynthetic process | 1 | 1 | 1 | 1 |
| GO:0006807~nitrogen compound metabolic process | 4 | 3 | 4 | 5 |
| GO:0006810~transport | 2 | 2 | 3 | 4 |
| GO:0006811~ion transport | 1 | 1 | 1 | 1 |
| GO:0006812~cation transport | 0 | 1 | 0 | 0 |
| GO:0006818~hydrogen transport | 1 | 1 | 1 | 1 |
| GO:0006836~neurotransmitter transport | 1 | 1 | 1 | 0 |
| GO:0006886~intracellular protein transport | 0 | 2 | 1 | 2 |
| GO:0006887~exocytosis | 1 | 1 | 1 | 1 |
| GO:0006904~vesicle docking during exocytosis | 0 | 1 | 1 | 1 |
| GO:0006950~response to stress | 0 | 1 | 3 | 3 |
| GO:0006952~defense response | 0 | 0 | 2 | 2 |
| GO:0006954~inflammatory response | 0 | 0 | 1 | 1 |
| GO:0006955~immune response | 0 | 0 | 2 | 2 |
| GO:0006956~complement activation | 0 | 0 | 1 | 1 |
| GO:0006958~complement activation, classical pathway | 0 | 0 | 1 | 1 |
| GO:0006959~humoral immune response | 0 | 0 | 1 | 1 |
| GO:0006974~response to DNA damage stimulus | 1 | 1 | 1 | 1 |
| GO:0007034~vacuolar transport | 1 | 1 | 1 | 1 |
| GO:0007154~cell communication | 1 | 1 | 1 | 0 |
| GO:0007167~enzyme linked receptor protein signaling pathway | 0 | 0 | 0 | 1 |
| GO:0007267~cell-cell signaling | 1 | 1 | 1 | 0 |
| GO:0007268~synaptic transmission | 1 | 1 | 1 | 0 |
| GO:0007269~neurotransmitter secretion | 1 | 1 | 1 | 0 |
| GO:0007623~circadian rhythm | 1 | 0 | 0 | 0 |
| GO:0008033~tRNA processing | 1 | 0 | 1 | 1 |
| GO:0008104~protein localization | 2 | 3 | 3 | 6 |
| GO:0008152~metabolic process | 2 | 3 | 2 | 3 |
| GO:0008380~RNA splicing | 2 | 2 | 2 | 1 |
| GO:0009056~catabolic process | 2 | 1 | 1 | 0 |
| GO:0009057~macromolecule catabolic process | 1 | 0 | 0 | 0 |
| GO:0009058~biosynthetic process | 3 | 4 | 2 | 2 |
| GO:0009059~macromolecule biosynthetic process | 2 | 3 | 3 | 2 |
| GO:0009060~aerobic respiration | 1 | 1 | 1 | 1 |
| GO:0009109~coenzyme catabolic process | 1 | 1 | 1 | 1 |
| GO:0009117~nucleotide metabolic process | 1 | 1 | 1 | 2 |
| GO:0009141~nucleoside triphosphate metabolic process | 1 | 1 | 1 | 1 |
| GO:0009142~nucleoside triphosphate biosynthetic process | 1 | 1 | 1 | 1 |
| GO:0009144~purine nucleoside triphosphate metabolic process | 1 | 1 | 1 | 1 |
| GO:0009145~purine nucleoside triphosphate biosynthetic process | 1 | 1 | 1 | 1 |
| GO:0009150~purine ribonucleotide metabolic process | 1 | 1 | 1 | 1 |
| GO:0009152~purine ribonucleotide biosynthetic process | 1 | 1 | 1 | 1 |
| GO:0009165~nucleotide biosynthetic process | 1 | 1 | 1 | 2 |
| GO:0009199~ribonucleoside triphosphate metabolic process | 1 | 1 | 1 | 1 |
| GO:0009201~ribonucleoside triphosphate biosynthetic process | 1 | 1 | 1 | 1 |
| GO:0009205~purine ribonucleoside triphosphate metabolic process | 1 | 1 | 1 | 1 |
| GO:0009206~purine ribonucleoside triphosphate biosynthetic process | 1 | 1 | 1 | 1 |
| GO:0009259~ribonucleotide metabolic process | 1 | 1 | 1 | 1 |
| GO:0009260~ribonucleotide biosynthetic process | 1 | 1 | 1 | 1 |
| GO:0009605~response to external stimulus | 0 | 0 | 1 | 1 |
| GO:0009607~response to biotic stimulus | 0 | 0 | 1 | 1 |
| GO:0009611~response to wounding | 0 | 0 | 1 | 1 |
| GO:0009615~response to virus | 0 | 0 | 1 | 1 |
| GO:0009889~regulation of biosynthetic process | 1 | 2 | 1 | 1 |
| GO:0009987~cellular process | 1 | 2 | 1 | 2 |
| GO:0010467~gene expression | 4 | 4 | 6 | 5 |
| GO:0010468~regulation of gene expression | 1 | 2 | 1 | 1 |
| GO:0010556~regulation of macromolecule biosynthetic process | 1 | 2 | 1 | 1 |
| GO:0015031~protein transport | 2 | 3 | 3 | 6 |
| GO:0015672~monovalent inorganic cation transport | 1 | 1 | 1 | 1 |
| GO:0015882~L-ascorbic acid transport | 1 | 0 | 0 | 0 |
| GO:0015931~nucleobase, nucleoside, nucleotide and nucleic acid transport | 1 | 1 | 1 | 1 |
| GO:0015980~energy derivation by oxidation of organic compounds | 1 | 1 | 1 | 1 |
| GO:0015985~energy coupled proton transport, down electrochemical gradient | 1 | 1 | 1 | 1 |
| GO:0015986~ATP synthesis coupled proton transport | 1 | 1 | 1 | 1 |
| GO:0015992~proton transport | 1 | 1 | 1 | 1 |
| GO:0016050~vesicle organization | 0 | 0 | 0 | 1 |
| GO:0016064~immunoglobulin mediated immune response | 0 | 0 | 1 | 1 |
| GO:0016070~RNA metabolic process | 4 | 3 | 5 | 5 |
| GO:0016071~mRNA metabolic process | 2 | 2 | 2 | 1 |
| GO:0016072~rRNA metabolic process | 0 | 0 | 3 | 3 |
| GO:0016079~synaptic vesicle exocytosis | 1 | 1 | 1 | 0 |
| GO:0016192~vesicle-mediated transport | 2 | 0 | 1 | 1 |
| GO:0016485~protein processing | 0 | 0 | 1 | 1 |
| GO:0016568~chromatin modification | 0 | 1 | 1 | 1 |
| GO:0016569~covalent chromatin modification | 0 | 0 | 0 | 1 |
| GO:0016570~histone modification | 0 | 0 | 0 | 1 |
| GO:0016573~histone acetylation | 0 | 0 | 0 | 1 |
| GO:0017156~calcium ion-dependent exocytosis | 1 | 0 | 0 | 0 |
| GO:0017157~regulation of exocytosis | 1 | 0 | 0 | 0 |
| GO:0019219~regulation of nucleobase, nucleoside, nucleotide and nucleic acid metabolic process | 1 | 2 | 1 | 1 |
| GO:0019222~regulation of metabolic process | 1 | 2 | 1 | 1 |
| GO:0019226~transmission of nerve impulse | 1 | 1 | 1 | 0 |
| GO:0019538~protein metabolic process | 1 | 3 | 2 | 3 |
| GO:0019724~B cell mediated immunity | 0 | 0 | 1 | 1 |
| GO:0022008~neurogenesis | 1 | 0 | 0 | 0 |
| GO:0022406~membrane docking | 0 | 1 | 1 | 1 |
| GO:0022607~cellular component assembly | 1 | 0 | 0 | 1 |
| GO:0022613~ribonucleoprotein complex biogenesis | 2 | 2 | 4 | 2 |
| GO:0022900~electron transport chain | 2 | 2 | 1 | 1 |
| GO:0030030~cell projection organization | 0 | 0 | 0 | 1 |
| GO:0030031~cell projection assembly | 0 | 0 | 0 | 1 |
| GO:0030163~protein catabolic process | 1 | 0 | 0 | 0 |
| GO:0031323~regulation of cellular metabolic process | 1 | 2 | 1 | 1 |
| GO:0031326~regulation of cellular biosynthetic process | 1 | 2 | 1 | 1 |
| GO:0032774~RNA biosynthetic process | 2 | 2 | 2 | 1 |
| GO:0032940~secretion by cell | 1 | 1 | 1 | 1 |
| GO:0032970~regulation of actin filament-based process | 0 | 0 | 1 | 1 |
| GO:0033036~macromolecule localization | 3 | 4 | 4 | 5 |
| GO:0033554~cellular response to stress | 1 | 1 | 1 | 1 |
| GO:0034220~ion transmembrane transport | 1 | 1 | 1 | 1 |
| GO:0034404~nucleobase, nucleoside and nucleotide biosynthetic process | 1 | 1 | 1 | 2 |
| GO:0034470~ncRNA processing | 1 | 0 | 2 | 3 |
| GO:0034613~cellular protein localization | 0 | 2 | 1 | 2 |
| GO:0034641~cellular nitrogen compound metabolic process | 4 | 3 | 4 | 5 |
| GO:0034645~cellular macromolecule biosynthetic process | 2 | 3 | 3 | 2 |
| GO:0034654~nucleobase, nucleoside, nucleotide and nucleic acid biosynthetic process | 1 | 1 | 1 | 2 |
| GO:0034660~ncRNA metabolic process | 1 | 0 | 2 | 3 |
| GO:0035188~hatching | 0 | 1 | 1 | 0 |
| GO:0042254~ribosome biogenesis | 2 | 2 | 4 | 3 |
| GO:0043170~macromolecule metabolic process | 3 | 5 | 3 | 4 |
| GO:0043254~regulation of protein complex assembly | 0 | 0 | 1 | 1 |
| GO:0043543~protein amino acid acylation | 0 | 0 | 0 | 1 |
| GO:0043967~histone H4 acetylation | 0 | 0 | 0 | 1 |
| GO:0043968~histone H2A acetylation | 0 | 0 | 0 | 1 |
| GO:0044085~cellular component biogenesis | 0 | 0 | 1 | 3 |
| GO:0044237~cellular metabolic process | 3 | 5 | 3 | 5 |
| GO:0044238~primary metabolic process | 2 | 4 | 3 | 3 |
| GO:0044248~cellular catabolic process | 2 | 1 | 1 | 0 |
| GO:0044249~cellular biosynthetic process | 2 | 3 | 3 | 2 |
| GO:0044257~cellular protein catabolic process | 1 | 0 | 0 | 0 |
| GO:0044260~cellular macromolecule metabolic process | 3 | 5 | 3 | 5 |
| GO:0044265~cellular macromolecule catabolic process | 1 | 0 | 0 | 0 |
| GO:0044267~cellular protein metabolic process | 1 | 3 | 1 | 2 |
| GO:0044271~nitrogen compound biosynthetic process | 1 | 1 | 1 | 2 |
| GO:0045087~innate immune response | 0 | 0 | 2 | 2 |
| GO:0045184~establishment of protein localization | 2 | 3 | 3 | 6 |
| GO:0045333~cellular respiration | 1 | 1 | 1 | 1 |
| GO:0045449~regulation of transcription | 1 | 2 | 1 | 1 |
| GO:0046034~ATP metabolic process | 1 | 1 | 1 | 1 |
| GO:0046356~acetyl-CoA catabolic process | 1 | 1 | 1 | 1 |
| GO:0046483~heterocycle metabolic process | 1 | 1 | 1 | 2 |
| GO:0046903~secretion | 1 | 1 | 1 | 1 |
| GO:0046907~intracellular transport | 2 | 2 | 3 | 4 |
| GO:0048278~vesicle docking | 0 | 1 | 1 | 1 |
| GO:0048489~synaptic vesicle transport | 1 | 1 | 1 | 0 |
| GO:0048511~rhythmic process | 1 | 0 | 0 | 0 |
| GO:0048518~positive regulation of biological process | 0 | 0 | 1 | 1 |
| GO:0048583~regulation of response to stimulus | 0 | 0 | 1 | 1 |
| GO:0048584~positive regulation of response to stimulus | 0 | 0 | 1 | 1 |
| GO:0050657~nucleic acid transport | 1 | 1 | 1 | 1 |
| GO:0050658~RNA transport | 1 | 1 | 1 | 1 |
| GO:0050776~regulation of immune response | 0 | 0 | 1 | 1 |
| GO:0050778~positive regulation of immune response | 0 | 0 | 1 | 1 |
| GO:0050794~regulation of cellular process | 0 | 1 | 1 | 1 |
| GO:0050877~neurological system process | 1 | 0 | 0 | 0 |
| GO:0050896~response to stimulus | 1 | 1 | 2 | 2 |
| GO:0051028~mRNA transport | 1 | 1 | 1 | 1 |
| GO:0051171~regulation of nitrogen compound metabolic process | 1 | 2 | 1 | 1 |
| GO:0051179~localization | 2 | 2 | 3 | 4 |
| GO:0051186~cofactor metabolic process | 1 | 1 | 1 | 1 |
| GO:0051187~cofactor catabolic process | 1 | 1 | 1 | 1 |
| GO:0051234~establishment of localization | 2 | 2 | 3 | 4 |
| GO:0051236~establishment of RNA localization | 1 | 1 | 1 | 1 |
| GO:0051276~chromosome organization | 0 | 1 | 1 | 1 |
| GO:0051603~proteolysis involved in cellular protein catabolic process | 1 | 0 | 0 | 0 |
| GO:0051604~protein maturation | 0 | 0 | 1 | 1 |
| GO:0051605~protein maturation by peptide bond cleavage | 0 | 0 | 1 | 1 |
| GO:0051641~cellular localization | 3 | 4 | 4 | 5 |
| GO:0051649~establishment of localization in cell | 3 | 3 | 4 | 5 |
| GO:0051704~multi-organism process | 0 | 0 | 1 | 1 |
| GO:0051707~response to other organism | 0 | 0 | 1 | 1 |
| GO:0051716~cellular response to stimulus | 1 | 1 | 1 | 1 |
| GO:0055085~transmembrane transport | 1 | 1 | 1 | 3 |
| GO:0055086~nucleobase, nucleoside and nucleotide metabolic process | 1 | 1 | 1 | 2 |
| GO:0055114~oxidation reduction | 1 | 1 | 1 | 1 |
| GO:0060255~regulation of macromolecule metabolic process | 1 | 2 | 1 | 1 |
| GO:0060627~regulation of vesicle-mediated transport | 1 | 0 | 0 | 0 |
| GO:0065008~regulation of biological quality | 1 | 1 | 1 | 0 |
| GO:0070727~cellular macromolecule localization | 0 | 2 | 1 | 2 |
| GO:0080090~regulation of primary metabolic process | 1 | 2 | 1 | 1 |

**^⊙^** denotes the number of times that each GO term occurred in the BA, CA, JA or vehicle group.
