# Supplementary material for: Quantitative Determination of Flexible Pharmacological Mechanisms Based On Topological Variation in Mice Anti-Ischemic Modular Networks
Source: PLoS One. 2016 Jul 6;11(7):e0158379. doi: 10.1371/journal.pone.0158379 (PMC4934924; doi:10.1371/journal.pone.0158379)
Supplement: S7 Table — (DOCX) [file pone.0158379.s008.docx]

**S7 Table. Overlapping and non-overlapping GO biological processes.**

| **Groups** | **Overlapping biological processes** |
| --- | --- |
| BA vs. CA vs. JA | Nuclear-transcribed mRNA catabolic process, nonsense-mediated decay, nuclear-transcribed mRNA catabolic process, regulation of neurotransmitter levels, generation of a signal involved in cell-cell signaling, RNA catabolic process, mRNA catabolic process, neurotransmitter transport, cell communication, cell-cell signaling, synaptic transmission, neurotransmitter secretion, catabolic process, synaptic vesicle exocytosis, transmission of nerve impulse, cellular catabolic process, synaptic vesicle transport and regulation of biological quality |
| **Groups** | **Independent biological processes** |
| BA | Circadian rhythm, macromolecule catabolic process, L-ascorbic acid transport, calcium ion-dependent exocytosis, regulation of exocytosis, neurogenesis, protein catabolic process, cellular protein catabolic process, cellular macromolecule catabolic process, rhythmic process, neurological system process, proteolysis involved in cellular protein catabolic process, regulation of vesicle-mediated transport |
| CA | Cation transport |
| JA | / |
| Vehicle | Protein amino acid acetylation, enzyme linked receptor protein signaling pathway, vesicle organization, covalent chromatin modification, histone modification, histone acetylation, cell projection organization, cell projection assembly, protein amino acid acylation, histone H4 acetylation, histone H2A acetylation |
