# Supplementary material for: Quantitative Determination of Flexible Pharmacological Mechanisms Based On Topological Variation in Mice Anti-Ischemic Modular Networks
Source: PLoS One. 2016 Jul 6;11(7):e0158379. doi: 10.1371/journal.pone.0158379 (PMC4934924; doi:10.1371/journal.pone.0158379)
Supplement: S8 Table — (DOCX) [file pone.0158379.s009.docx]

**S8 Table. Overlapping and non-overlapping KEGG pathways.**

| **Groups** | **Overlapping KEGG** **pathways** |
| --- | --- |
| BA vs. CA vs. JA vs. V | Alzheimer's disease, Basal transcription factors, Carbon metabolism, Citrate cycle (TCA cycle), Collecting duct acid secretion, Epstein-Barr virus infection, Fc gamma R-mediated phagocytosis, Glyoxylate and dicarboxylate metabolism, Huntington's disease, Legionellosis, Lysosome, Metabolic pathways, mRNA surveillance pathway, Nucleotide excision repair, Oxidative phosphorylation, Parkinson's disease, Phagosome, Propanoate metabolism, Proteasome, Protein processing in endoplasmic reticulum, Purine metabolism, Pyrimidine metabolism, Renal cell carcinoma, Rheumatoid arthritis, Ribosome, Ribosome biogenesis in eukaryotes, RNA polymerase, RNA transport, SNARE interactions in vesicular transport, Spliceosome, Sulfur relay system, Synaptic vesicle cycle, Ubiquitin mediated proteolysis, Valine, leucine and isoleucine degradation |
| CA vs. JA vs. V | Biosynthesis of amino acids, Endocytosis, Pentose phosphate pathway |
| BA vs. JA vs. V | Cytosolic DNA-sensing pathway, Herpes simplex infection |
| BA vs. CA vs. JA | Amphetamine addiction, Insulin secretion, Regulation of autophagy, RNA degradation |
| BA vs. JA | Circadian rhythm, Dopaminergic synapse |
| CA vs. JA | Glutathione metabolism |
| BA vs. CA | Caffeine metabolism, Drug metabolism-other enzymes, Pancreatic cancer, Pathways in cancer, Peroxisome |
| BA vs. V | Alanine, aspartate and glutamate metabolism, Amino sugar and nucleotide sugar metabolism, Axon guidance, Butanoate metabolism, Influenza A, N-Glycan biosynthesis, Tight junction |
| JA vs. V | Bacterial invasion of epithelial cells, Chagas disease (American trypanosomiasis), Complement and coagulation cascades, Pertussis, Prion diseases, Regulation of actin cytoskeleton, Salmonella infection, Staphylococcus aureus infection, Systemic lupus erythematosus |
| CA vs. V | Base excision repair, NF-kappa B signaling pathway, Non-homologous end-joining, Tuberculosis, Wnt signaling pathway |
| **Groups** | **Independent KEGG pathways** |
| BA | Circadian entrainment, ErbB signaling pathway, GABAergic synapse, Galactose metabolism, MicroRNAs in cancer, Oocyte meiosis, Salivary secretion, Sphingolipid metabolism, Synthesis and degradation of ketone bodies, Vasopressin-regulated water reabsorption, and Vitamin digestion and absorption |
| CA | Aminoacyl-tRNA biosynthesis, Chemical carcinogenesis, Cyanoamino acid metabolism, Drug metabolism-cytochrome P450, Glycine, serine and threonine metabolism, HIF-1 signaling pathway, Metabolism of xenobiotics by cytochrome P450, mTOR signaling pathway, NOD-like receptor signaling pathway, One carbon pool by folate |
| JA | Adipocytokine signaling pathway, Biosynthesis of unsaturated fatty acids, Cocaine addiction, Estrogen signaling pathway, Fatty acid elongation, Hepatitis B, Hypertrophic cardiomyopathy (HCM), Insulin signaling pathway, Measles, Riboflavin metabolism, Steroid hormone biosynthesis, TNF signaling pathway, and Vitamin B6 metabolism |
| V | Adherens junction, Amoebiasis, Chronic myeloid leukemia, Focal adhesion, Glycerophospholipid metabolism, Inositol phosphate metabolism, Notch signaling pathway, Pantothenate and CoA biosynthesis, Phosphatidylinositol signaling system, Protein export, Steroid biosynthesis, Terpenoid backbone biosynthesis, Tyrosine metabolism, Viral carcinogenesis |

Note: V = the vehicle group.
