# Supplementary material for: Quantitative Determination of Flexible Pharmacological Mechanisms Based On Topological Variation in Mice Anti-Ischemic Modular Networks
Source: PLoS One. 2016 Jul 6;11(7):e0158379. doi: 10.1371/journal.pone.0158379 (PMC4934924; doi:10.1371/journal.pone.0158379)
Supplement: S9 Table — (DOCX) [file pone.0158379.s010.docx]

**S9 Table. Relationship between compounds and KEGG pathways in the watershed allosteric modules supported by previous literature.**

| **Compounds** | **Enriched KEGG pathways in the watershed modules** | **The relationships between compounds and pathways** | **References** |
| --- | --- | --- | --- |
| **BA** | Nucleotide excision repair | Our previous findings indicate that baicalin may accelerate CPD removal after UVB irradiation. We speculate that downregulation of miR-181b and miR-378 by baicalin may upregulate cyclobutane pyrimidine dimmers (CPD) removal via the NER signaling pathway. | [1] |
|  | Basal transcription factors | Nuclear factor-κB is a pivotal transcription factor in chronic inflammatory diseases. Baicalin inhibited nuclear factor κB (NF-κB) activation. | [2] |
|  | Viral carcinogenesis | Baicalin has been shown to have anti-oxidative, anti-bacterial, anti-viral, anti-carcinogenic and anti-allergic properties. | [3] |
|  | Oxidative phosphorylation | Not reported | — |
|  | Parkinson's disease | The preventive medication of baicalin shows a protective effect on C57 BL mouse with Parkinson’s disease induced by MPTP. | [4] |
|  | Alzheimer's disease | Baicalin may be a potential agent for inhibiting Aβ aggregation and may thereby delay, mitigate or modify the progression of neurodegenerative diseases such as Alzheimer’s disease (AD). | [5] |
|  | Huntington's disease | Not reported | — |
|  | Sulfur relay system | Not reported | — |
|  | RNA transport | Not reported | — |
|  | Ribosome biogenesis in eukaryotes | Not reported | — |
| **CA** | Nucleotide excision repair | Not reported | — |
|  | Basal transcription factors | The farnesoid X receptor (FXR) is a member of the steroid/thyroid hormone receptor family of ligand-activated transcription factors that is activated by bile acids, including cholic acid and chenodeoxycholic acid. | [6] |
|  |  | Bile acids are ligands of farnesoid X receptor (FXR), a nuclear receptor of ligand-activated transcription factor. Bile acids and FXR not only interact with each other but also regulate various downstream targets independently during liver regeneration. | [7] |
|  |  | Sp1 was initially identified as a basal transcription factor. In conclusion, Sp1 is a bile acid-responsive transcription factor that mediates DR5/TRAIL-R2 gene expression downstream of JNK. | [8] |
|  |  | The three significant pathways only activated in ursodeoxycholic acid-treated group (UA): Huntington’s disease, Fc epsilon RI signaling pathway, basal transcription factors. | [9] |
|  | Viral carcinogenesis | Bile acids activate Yes-associated protein (YAP) to promote liver carcinogenesis. | [10] |
|  |  | Ursodeoxycholic acid (UDCA) and gugglesterone are other candidates for chemoprevention of esophageal carcinogenesis. | [11] |
|  | Oxidative phosphorylation | Bile acids robustly increase mitochondrial activity and oxidative phosphorylation in brown adipose tissue (in rodents) and skeletal muscle cells (in humans). | [12] |
|  |  | One of the primary bile acids, chenodeoxycholic acid (CDCA) induces antioxidant and xenobiotic-metabolizing enzymes by activating C/EBPβ through phosphorylation. | [7] |
|  | Parkinson's disease | Tauroursodeoxycholic acid improves the survival and function of nigral transplants in a rat model of Parkinson’s disease. | [13] |
|  | Alzheimer's disease | These studies provide evidence for the anti-apoptotic role of bile acids in experimental Alzheimer’s disease (AD). Ursodeoxycholic acid and tauroursodeoxycholic acid are well-characterized, potent inhibitors of apoptosis in different cell types, and their effects have expanded to several experimental models of neurological disorders, including AD. | [14] |
|  | Huntington's disease | Tauroursodeoxycholic acid, a bile acid, is neuroprotective in a transgenic animal model of Huntington's disease. | [15] |
|  | Sulfur relay system | Not reported | — |
| **JA** | Nucleotide excision repair | Not reported | — |
|  | Basal transcription factors | Not reported | — |
|  | Viral carcinogenesis | Geniposide has been shown to possess anti-inflammatory, anti-oxidant, anti-carcinogenic and anti-angiogenic activities. | [16] |
|  | RNA transport | Not reported | — |
|  | Ribosome biogenesis in eukaryotes | Not reported | — |

**Supplemental References (S9 Table)**

1. Xu Y, Zhou B, Wu D., Yin Z, Luo D. Baicalin modulates microRNA expression in UVB irradiated mouse skin. J Biomed Res. 2012; 26: 125-134.

2. Zhao S, Li H, Liu Z, Wang J, Wang X, Qin M, Zhao B, Tang H, Wen Q. Baicalin inhibited nuclear factor κB (NF-κB) activation and attenuated sodium taurocholate of induced experimental pancreatitis in rats. Afr J Pharm Pharmaco. 2012; 6: 1176-1185.

3. Zhu ML, Liang XL, Zhao LJ, Liao ZG, Zhao GW, Cao YC., Zhang J, Luo Y. Elucidation of the transport mechanism of baicalin and the influence of a Radix Angelicae Dahuricae extract on the absorption of baicalin in a Caco-2 cell monolayer model. J Ethnopharmacol. 2013; 150: 553-559.

4. Chen X, Zhang N, Zou HY. (2007). Protective effect of baicalin on mouse with Parkinson’s disease induced by MPTP. Chinese journal of integrated traditional and western medicine. 2007; 27: 1010-1012.

5. Yin F, Liu J, Ji X, WangY, Zidichouski J, Zhang J. Baicalin prevents the production of hydrogen peroxide and oxidative stress induced by Abeta aggregation in SH-SY5Y cells. Neurosci Lett. 2011; 492: 76-79.

6. Inagaki T, Moschetta A, Lee YK, Peng L, Zhao G, Downes M, Yu RT, Shelton JM, Richardson JA, Repa JJ, Mangelsdorf DJ, Kliewer SA. Regulation of antibacterial defense in the small intestine by the nuclear bile acid receptor. Proc Natl Acad Sci USA. 2006; 103: 3920-3925.

7. Li G, Guo GL. Farnesoid X receptor, the bile acid sensing nuclear receptor, in liver regeneration. Acta Pharm Sin B. 2015; 5: 93-98.

8. Higuchi H, Grambihler A, Canbay A, Bronk SF, Gores GJ. Bile Acids Up-regulate Death Receptor 5/TRAIL-receptor 2 Expression via a c-Jun N-terminal Kinase-dependent Pathway Involving Sp1. J Biol Chem. 2004; 279: 51-60.

9. Liu J, Zhou CX, Zhang ZJ, Wang LY, Jing ZW, Wang Z. Synergistic Mechanism of Gene Expression and Pathways between Jasminoidin and Ursodeoxycholic Acid in Treating Focal Cerebral Ischemia-Reperfusion Injury. CNS Neurosci Ther. 2012; 18: 674-682.

10. Anakk S, Bhosale M, Schmidt VA, Johnson RL, Finegold MJ, Moore DD. Bile acids activate YAP to promote liver carcinogenesis. Cell Rep. 2013; 5: 1060-1069.

11. Fujimura T, Oyama K, Sasaki S, Nishijima K, Miyashita T, Ohta T, Miwa K, Hattori T. Inflammation-Related Carcinogenesis and Prevention in Esophageal Adenocarcinoma Using Rat Duodenoesophageal Reflux Models. Cancers. 2011; 3: 3206-3224.

12. Houten SM, Watanabe M, Auwerx J. Endocrine functions of bile acids. The EMBO Journal. 2006; 25: 1419-1425.

13.Duan WM, Rodrigures CMP, Zhao LR, Steer CJ, Low WC. Tauroursodeoxycholic Acid Improves the Survival and Function of Nigral Transplants in a Rat Model of Parkinson’s Disease. Cell Transplant. 2002; 11: 195-205.

14. Ramalho RM, Viana RJS, Low WC, Steer CJ, Rodrigues CMP. Bile acids and apoptosis modulation: an emerging role in experimental Alzheimer’s disease. Trends Mol Med. 2008; 14: 54-62.

15. Keene CD, Rodrigues CMP, Eich T, Chhabra MS, Steer CJ, Low WC. Tauroursodeoxycholic acid, a bile acid, is neuroprotective in a transgenic animal model of Huntington’s disease. Proc Natl Acad Sci USA. 2002; 99: 10671-10676.

16. Kim J, Kim HY, Lee SM. Protective Effects of Geniposide and Genipin against Hepatic Ischemia/Reperfusion Injury in Mice. Biomol Ther. 2013; 21: 132-137.
